# Supplementary material for: Seroprevalence of hepatitis E virus infection in the Americas: Estimates from a systematic review and meta-analysis
Source: PLoS One. 2022 Jun 1;17(6):e0269253. doi: 10.1371/journal.pone.0269253 (PMC9159553; doi:10.1371/journal.pone.0269253)
Supplement: S1 File — (PDF) [file pone.0269253.s001.pdf]

Supplementary Material

Table of Contents

Supplementary Material 1
1. Search strategy 1
1.1. Scielo 1
1.2. Lilacs 1
1.3. Ebscohost open dissertations 1
1.4. Cochrane Library 1
1.5. PubMed 2
2. Available Systematic Reviews 3
3. Risk of bias assessment 4
4. Characteristics of the included studies 9
5. Risk factors 37
6. Genotypes found in the literature 59
7. Publication bias 60
8. Overview of results obtained by different tests 62
9. Percentages of Total anti-HEV positive results 63
10. Overview of NHANES results 65
11. Percentages of IgG positive results 66
12. Percentages of IgM positive results in total sample 74
13. Percentages of IgM positive results among IgG or total anti-HEV positive samples 76
14. Percentages of RNA positive results in total sample 78
15. Percentages of RNA positive results among IgG and/or IgM positive samples 79
16. References 80

1. Search strategy

1.1. Scielo

25-05-2021= (ab:((ab:(((ab:(hepatitis e virus)) OR (ab:(hev)) OR (ab:(hepatitis e))) AND (ab:(seroprevalence)))))) = 168 results

1.2. Lilacs

25-05-2021= (Hepatitis e virus) OR (hev) OR (Hepatitis e) AND (seroprevalence)= 244 results

1.3. Ebscohost open dissertations

25-05-2021= hepatitis e AND seroprevalence = 8 (1 duplicate)

1.4. Cochrane Library

25-05-2021= MeSH descriptor: [Hepatitis E virus] explode all trees = 12

1.5. PubMed

Table 1. Search in PubMed May 2021

Search number	Query	Search Details	Results
5	((("Hepatitis E virus"[Mesh]) OR (((HEV[Title/Abstract]) OR (hepatitis e[Title/Abstract])) OR (hepatitis e virus[Title/Abstract]))) AND ((seroprevalence[Title/Abstract]) OR (prevalence[Title/Abstract]))) AND (((Human[Title/Abstract]) OR (Population[Title/Abstract])) OR (people[Title/Abstract]))	("Hepatitis E virus"[MeSH Terms] OR ("HEV"[Title/Abstract] OR "hepatitis e"[Title/Abstract] OR "Hepatitis E virus"[Title/Abstract])) AND ("seroprevalence"[Title/Abstract] OR "prevalence"[Title/Abstract]) AND ("Human"[Title/Abstract] OR "Population"[Title/Abstract] OR "people"[Title/Abstract])	780
4	((Human[Title/Abstract]) OR (Population[Title/Abstract])) OR (people[Title/Abstract])	"Human"[Title/Abstract] OR "Population"[Title/Abstract] OR "people"[Title/Abstract]	4,355,309
3	(seroprevalence[Title/Abstract]) OR (prevalence[Title/Abstract])	"seroprevalence"[Title/Abstract] OR "prevalence"[Title/Abstract]	682,232
2	((HEV[Title/Abstract]) OR (hepatitis e[Title/Abstract])) OR (hepatitis e virus[Title/Abstract])	"HEV"[Title/Abstract] OR "hepatitis e"[Title/Abstract] OR "hepatitis e virus"[Title/Abstract]	7,284
1	"Hepatitis E virus"[Mesh]	"Hepatitis E virus"[MeSH Terms]	3,420

2. Available Systematic Reviews

Table 2. Available Systematic Reviews

First Author	Journal	Year of publication	Focus	Countries from the Americas included	Number of studies from the Americas
Bigna, JJ et al [1].	BMC Pregnancy Childbirth	2020	Pregnancy and maternofetal outcomes	Brazil and Venezuela	2
Capai, L et al [2].	Viruses	2019	Industrialized Countries	United States and Canada	7
Carrilho, FJ et al [3].	Gastroenterol Hepatol	2005	Brazil	Brazil	10
Horvatits, T et al[4].	Liver Int	2018	America	United States, Mexico, Guatemala, Honduras, Nicaragua, Argentina, Bolivia, Brazil, Chile, Colombia, Peru, Uruguay, Venezuela, Carabean, Cuba, French Guyana, Haiti	68
Li, P et al [5].	Liver Int	2020	Global	Guyana, Brazil, Bolivia, Venezuela, Argentina, Chile, Nicaragua, Canada, United States, Mexico, Cuba	45
Remondegui, C et al [6].	Rev Argent Microbiol	2021	South America	Brazil, Venuezuela, Uruguay, Peru, Guyana Francesa, Colombia, Chile, Bolivia, Argentina	27
Tavakoli, A et al [7].	Ther Apher Dial	2020	Patients undergoing maintenance hemodialysis	Brazil and Argentina	7
Tengan, FM et al [8].	Infect Dis Poverty	2019	Adults in Brazil	Brazil	14
Verghese, VP et al [9].	Clin Infect Dis	2014	Children	Mexico, United States, Venezuela, Argentina, Bolivia, Cuba	7
Wilhelm, B et al [10].	PLoS One	2019	General population across non-endemic countries	Argentina, Canada, United States	42
Wilhelm, B et al [11].	Zoonoses Public Health	2020	Predictors of human exposure in non-endemic countries	Argentina, Canada, Chile, United States	23

3. Risk of bias assessment

Table 3. Reported HEV Genotypes

Studies	Bias due to confounding	Bias due to selection of participants and follow-up	Bias due to misclassification of exposure	Bias due to missing data	Bias in measurement of outcome	Bias in selection of the reported
	**low** if stratification/adjustment for age - **moderate** if age information - **high** if no age information	**low** if follow-up well documented for all risk groups up to intended follow-up time - **moderate** if not well reported for all groups - **high** if different follow-up time for different groups or follow-up not until endpoint for all groups	**low** if medical records - **moderate** if self-reported - **high** if unclear source	**low** if outcome data available for all and missing data reported - **moderate** if some missing data reported but not all - **high** if participants excluded on large scale for missing data	**low** if outcome definition reported and well classified , **moderate** if no report, but clear meaning, **high** if no report available	**low** if subgroup reporting seems adequate and not selective, **high** if selective subgroup reporting
Alvarado-Esquivel, C et al. (2021) JMV	Moderate	Moderate	Low	Moderate	Low	Moderate
Alvarado-Esquivel, C et al. (2021) GR	Low	Moderate	Low	Moderate	Low	Low
Alvarado-Esquivel, C et al. (2020)	Low	Moderate	Low	Moderate	Low	Low
Alvarado-Esquivel, C et al. (2014)	Low	Moderate	Low	Moderate	Low	Low
Alvarado-Esquivel, C et al. (2015)	Low	Moderate	Low	Moderate	Low	Low
Alvarez-Munoz, MT et al.	Low	Moderate	Low	Moderate	Moderate	Low
Andonov, A et al.	Moderate	Moderate	Low	Low	Low	Low
Anugwom, C et al.	Moderate	Moderate	Low	Moderate	Moderate	Moderate
Arce, L. P et al.	Moderate	Moderate	Low	Moderate	Low	Low
Assis, SB et al.	Moderate	Moderate	Low	Moderate	Low	Low
Atiq, M	Low	Moderate	Low	Low	Low	Low
Bangueses, F et al.	Low	Moderate	Low	Low	Low	Low
Baptista-González, H et al.	High	Moderate	Low	Moderate	Low	Moderate
Bartoloni, A et al	Low	Moderate	Low	Moderate	Low	Low
Betancur, CA et al.	Moderate	Moderate	Low	Low	Low	Low
Bezerra, LA et al.	Low	Low	Low	Low	Low	Low
Blitz-Dorfman, L et al.	High	Moderate	Low	Moderate	Low	Moderate
Bortoliero, AL et al	Low	Moderate	Low	Low	Low	Low

Brahm, J et al.	High	Moderate	Low	Low	Low	Low
Bricks, G et al. (2018)	Low	Moderate	Low	Moderate	Low	Low
Bricks, G et al. (2019)	Low	Moderate	Low	Moderate	Low	Low
Cabezas, C et al.	Moderate	Moderate	Low	Moderate	Low	Low
Caetano, K et al.	Moderate	Moderate	Low	Moderate	Low	Low
Campbell, C.	Low	Moderate	Low	Moderate	Low	Moderate
Campolmi, I et al.	Low	Moderate	Low	Moderate	Low	Low
Cangin, C et al.	Low	Moderate	Low	Moderate	Low	Low
Cordova, C et al.	High	Moderate	Low	Low	Low	Low
Cossaboom, CM et al.	Low	Moderate	Low	Low	Low	Low
Covarrubías, N et al. (2015)	Low	Moderate	Low	Low	Low	Low
Covarrubías, N et al. (2018)	Low	Moderate	Low	Low	Low	Moderate
de Almeida, E. Araujo DC et al.	Low	Moderate	Low	Moderate	Low	Low
de la Caridad Montalvo Villalba, M et al. (2013)	Low	Moderate	Low	Moderate	Low	Low
de la Caridad Montalvo Villalba, M et al. (2010)	Low	Moderate	Low	Low	Low	Low
de la Caridad Montalvo Villalba, M et al. (2008)	Moderate	Moderate	Low	Low	Low	Moderate
Montalvo-Villalba, MC et al. (2016)	High	Moderate	Low	Low	Low	Moderate
de Oliveira, Jmns et al.	Moderate	Low	Low	Low	Low	Low
de Paula, VS et al.	Moderate	Moderate	Low	Low	Low	Low
Debes, JD et al.	Moderate	Moderate	Low	Moderate	Low	Moderate
Dell'Amico, MC et al.	Moderate	Moderate	Low	Low	Low	Low
Di Lello, FA et al.	Moderate	Moderate	Low	Low	Low	Low
Diehl, TM et al.	Low	Moderate	Low	Moderate	Low	Low
Ditah, I et al.	Low	Moderate	Low	Moderate	Low	Low
Eick, A et al.	Moderate	Low	Low	Moderate	Low	Low
Engle, RE et al.	High	Moderate	Low	Low	Moderate	Moderate
Fainboim, H et al.	Moderate	Moderate	Low	Low	Low	Moderate
Fantilli, AC et al.	Moderate	Moderate	Low	Moderate	Low	Low
Fearon, MA et al.	Moderate	Moderate	Low	Low	Low	Low
Ferreira, AC et al.	Moderate	Moderate	Low	Moderate	Low	Low

Focaccia, R et al.	Moderate	Moderate	Low	Moderate	Moderate	Moderate
Fontana, RJ et al.	Low	Low	Low	Moderate	Low	Low
Freitas, NR et al. (2016)	Low	Moderate	Low	Moderate	Low	Low
Freitas, NR, et al (2017)	Moderate	Moderate	Low	Moderate	Low	Low
Gambel, JM et al.	Moderate	Moderate	Low	Moderate	Low	Moderate
Gandolfo, GM et al.	Moderate	Moderate	Low	Low	Moderate	Moderate
Garcia, CG et al.	Moderate	Moderate	Low	Low	Low	Low
Goncales, NS et al.	Moderate	Moderate	Low	Moderate	Low	Low
Gutiérrez-Vergara, CC et al.	Moderate	Moderate	Low	Moderate	Low	Low
Guzmán Rojas, P et al.	Low	Moderate	Low	Moderate	Low	Low
Halac, U et al.	Moderate	Low	Low	Low	Moderate	Moderate
Hardtke, S et al.	Low	Moderate	Low	Low	Low	Moderate
Hering, T et al.	Moderate	Moderate	Low	Low	Low	Low
Hurtado, C et al.	High	Moderate	Low	Low	Low	Moderate
Hyams, KC et al.	Moderate	Moderate	Low	Moderate	Low	Moderate
Ibarra V, H et al. (2001)	Moderate	Low	Low	Low	Low	Low
Ibarra, H et al. (1997)	Moderate	Moderate	Low	Low	Low	Low
Ibarra, H et al. (2006)	Moderate	Low	Low	Moderate	Low	Moderate
Karetnyi, YV et al.	High	Moderate	Low	Low	Moderate	Low
Kiesslich, D et al.	Moderate	Moderate	Low	Moderate	Moderate	Moderate
Koning, L et al.	Moderate	Low	Low	Low	Low	Low
Konomi, N et al.	Low	Moderate	Low	Low	Moderate	Moderate
Kuniholm, MH et al. (2009)	Low	Moderate	Low	Moderate	Moderate	Moderate
Kuniholm, M. H et al (2016)	Moderate	Low	Low	Low	Low	Low
Kyvernitakis, A et al.	Moderate	Moderate	Low	Moderate	Low	Low
Langer, BC et al.	Low	Moderate	Low	Low	Low	Low
Lemos, G et al.	High	Moderate	Low	Low	Low	Moderate
León, P et al.	Moderate	Moderate	Low	Moderate	Low	Low
Lopes Dos Santos, DR et al.	High	Moderate	Low	Moderate	Low	Moderate
Lopez-Santaella, T et al.	High	High	Low	Low	Low	Low
Lyra, AC et al.	Moderate	Moderate	Low	Low	Low	Low
Mahajan, R et al.	Moderate	Moderate	Low	Moderate	Low	Low

Martinez Wassaf, MG et al.	Low	Moderate	Low	Moderate	Low	Moderate
Martinez, AP et al.	Low	Moderate	Low	Low	Low	Low
Martins, RM et al.	Low	Moderate	Low	Moderate	Low	Low
Mast, EE	Moderate	Moderate	Low	Moderate	Moderate	Moderate
McGivern, DR et al.	Low	Low	Low	Low	Low	Low
Meng, XJ et al.	Low	Moderate	Low	Moderate	Moderate	Low
Miernyk, KM et al.	Moderate	Moderate	Low	Moderate	Low	Low
Minuk, GY et al.	Moderate	Moderate	Low	Low	Low	Moderate
Moraes dos Santos, DC et al.	Moderate	Moderate	Low	Low	Low	Low
Munne, MS et al. (2014)	Moderate	Moderate	Low	Low	Low	Low
Munne, MS et al. (2011)	Moderate	Moderate	Low	Moderate	Low	Moderate
Ooi, WW et al.	Moderate	Moderate	Low	Moderate	Moderate	Low
Pandolfi, R et al.	High	Moderate	Low	Low	Low	Moderate
Panduro, A et al.	High	Moderate	Low	Low	Low	Low
Pang, L et al.	Moderate	Moderate	Low	Low	Low	Moderate
Parana, R et al. (1997)	Moderate	Moderate	Low	Low	Low	Low
Parana, R et al. (1999)	Moderate	Low	Low	Low	Low	Moderate
Passos, AM et al.	Moderate	Moderate	Low	Low	Low	Moderate
Passos-Castilho, AM et al. (2016) BJID	Moderate	Moderate	Low	Low	Low	Low
Passos-Castilho, AM et al. (2016) JMV	Low	Moderate	Low	Low	Low	Low
Passos-Castilho, AM et al. (2017)	Low	Moderate	Low	Low	Low	Low
Passos-Castilho, AM et al. (2015)	Moderate	Moderate	Low	Moderate	Low	Low
Pelaez, D et al. (2014)	Moderate	Moderate	Low	Low	Low	Low
Pelaez, D et al. (2015)	Moderate	Moderate	Low	Low	Low	Low
Perez, OM et al.	Low	Moderate	Low	Low	Moderate	Low
Pisano, MB et al.	Moderate	Moderate	Low	Moderate	Low	Low
Pisano, MB et al (2018)	High	Moderate	Low	Moderate	Low	Low
Pujol, FH et al. (1994) GEN	High	Moderate	Low	Low	Moderate	Moderate
Pujol, FH et al. (1994) JMV	Moderate	Moderate	Low	Moderate	Moderate	Low
Quintana, A et al.	Moderate	Moderate	Low	Moderate	Moderate	Moderate

Realpe-Quintero, M et al.	Moderate	Moderate	Low	Low	Low	Low
Redlinger, T et al.	Low	Low	Low	Moderate	Low	Moderate
Remondegui, C et al.	Moderate	Moderate	Low	Moderate	Low	Low
Rendon, J et al.	Moderate	Moderate	Low	Moderate	Low	Low
Rey, JA et al.	Moderate	Moderate	Low	Low	Moderate	Moderate
Rodriguez Lay, L et al.	High	Moderate	Low	Low	Low	Moderate
Saraceni, CP.	Moderate	Moderate	Low	Low	Moderate	Low
Sherman, KE et al. (2014)	Moderate	Moderate	Low	Low	Low	Moderate
Sherman, K. E et al (2021)	Moderate	High	Low	High	Low	Low
Silva, SM et al.	Low	Moderate	Low	Moderate	Low	Low
Smalligan, RD et al.	Moderate	Low	Low	Low	Low	Low
Smith, HM et al.	Moderate	Moderate	Low	Low	Moderate	Moderate
Souto, FJ et al. (1998)	Moderate	Moderate	Low	Moderate	Low	Moderate
Souto, FJ et al. (1997)	Moderate	Moderate	Low	Moderate	Low	Moderate
Stramer, SL et al.	Low	Moderate	Low	Low	Low	Low
Sue, PK et al.	Moderate	Low	Low	Moderate	Low	Low
Talarmin, A et al.	Moderate	Moderate	Low	Low	Moderate	Low
Tejada-Strop, A et al.	Moderate	Moderate	Low	Low	Low	Low
Teshale, EH et al.	Low	Moderate	Low	Moderate	Low	Low
Thomas, DL et al.	Low	Moderate	Low	Moderate	Moderate	Moderate
Tissera, G et al.	Moderate	Moderate	Low	Low	Low	Low
Trinta, KS et al.	Moderate	Moderate	Low	Low	Low	Low
Unzueta, A et al.	Moderate	Moderate	Low	Low	Low	Low
Vildosola, H et al.	Moderate	Moderate	Low	Moderate	Low	Low
Vitral, CL et al. (2014)	Moderate	Moderate	Low	Moderate	Low	Low
Vitral, CL et al. (2005)	High	Moderate	Low	Low	Moderate	Moderate
Withers, MR et al.	Moderate	Moderate	Low	Low	Moderate	Low
Xu, C et al.	Moderate	Moderate	Low	Moderate	Low	Low
Zafrullah, M et al.	Moderate	Moderate	Low	Moderate	Low	Moderate
Zhang, L et al.	Moderate	Moderate	Low	Moderate	Moderate	Low

4. Characteristics of the included studies

Table 4. Characteristics of the included studies

First author	Journal	Year of publication	Region	Country	Study type	Start of study	End of study	Sample	Event	Population group	Population group Classified	Median age	Proportion of male	Studied Parameters	Assay	Meta-Analysis
Alvarado-Esquivel, C et al [12].	Hepat Mon	2014	North America	Mexico	Cross-sectional study	Dec-06	Aug-07	273	100	Adults in rural areas	Rural population	Mean age (SD): 39.85 (17.15) Range: 18 to 91 years	204 women 69 men	IgG	Diagnostic Automation Inc.	IgG-R
Alvarado-Esquivel, C et al [13].	Gastroenterol Res	2021	North America	Mexico	Age- and gender-matched case-control study	-	-	101	18	Butchers	Occupational group	Mean age (SD): 38.50 (12.52) Range: 16 to 71 years	17 women 84 men	IgG	Diagnostic Automation Inc.	IgG-OG
								101	14	People without an occupation of butcher	General population	Mean age (SD): 38.43 (14.13) Range: 18 to 71 years	17 women 84 men			IgG-GP
Alvarado-Esquivel, C et l [14].	J Med Virol	2021	North America	Mexico	Case-control study	-	-	86	8	Control subjects without waste picker occupation (controls)	General population	Mean age (SD): 36.05 (17.02) Range: 16 to 78 years	54 (62.8%) women 32 (37.2%) men	IgG	Diagnostic Automation Inc.	IgG-GP
								86	14	Waste pickers (cases)	Occupational group	Mean age (SD): 35.45 (17.15) Range: 14 to 76 years	54 (62.8%) women 32 (37.2%) men			IgG-OG
Alvarado-Esquivel, C et al [15].	J Clin Med Res	2015	North America	Mexico	Case-control study (population of toxoplasmosis study)	-	-	150	61	Age- and gender-matched non-Mennonites controls	Rural population	Mean age (SD): 40.83 (18.81) Range: 18 to 91 years.	71 women 79 men	IgG	Diagnostic Automation Inc.	Excluded Subsample of (reference 12)
								150	10	Mennonites	Occupational group	Mean age (SD): 38.40 (15.53) Range: 17 to 85 years	60 women 90 men			IgG-OG
Alvarado-Esquivel, C et al [16].	Ann Hepatol	2020	North America	Mexico	Case-control seroprevalence study	2006	2014	146	46	Age- and gender-matched control subjects of the	Rural population	Mean age (SD): 32.23 (13.76) Range: 18 to 78 years	84 women 62 men	IgG	Diagnostic Automation Inc.	IgG-R

										general population from rural settings.						
								146	5	Tepehuanos	Ethnic groups	Mean age (SD): 30.65 (16.38) Range: 15 to 89 years	84 women 62 men			IgG-EG
Alvarez-Munoz, MT et al [17].	Arch Med Res	1999	North America	Mexico	Community-based survey	1987	1988	3549	374	Young adults and children	General population	Range: 1 to 29 years	2200 women 1349 men	IgG	Abbott Laboratories	IgG-GP
Andonov, A et al [18].	Vox Sang	2014	North America	Canada	Retrospective study	2001	2003	38	0	Patients received either solvent–detergent-treated plasma prepared by pooling of 2500 single-donor or cryosupernatant plasma.	Immunodeficiency	Median age: 49 Range: 23 to 82 years	Male : female ratio was 1:2,1	IgG	MP Diagnostic	IgG-I
								38	0					IgM		IgMT-I
Anugwom, C et al [19].	J Viral Hepat	2021	North America	United States	-	2009	2016	-		NHANES population	General population	HEV IgG positives: Mean age: 59.3 years (95% CI 58.3–60.2). HEV IgG negatives: Mean age: 39.9 years (95% CI 39.3–40.4)	HEV IgG positives: 53.7% women. HEV IgG negatives : 51% women	IgG and IgM	Diagnostic System	Excluded Duplicate database-NHANES, no standard deviation or CI reported
Arce, L. P et al [20].	Front Microbiol	2019	South America	Argentina	-	2017	2017	197	30	Panel of serum samples for performance analysis	Multiple	-	-	IgG	In house	Excluded test evaluation
								813	75	Blood donors	Blood donors	Median age: 35 34.22% were between 26 and 35 years old	73.05% men			IgG-BD
Assis, SB et al [21].	Rev Soc Bras Med Trop	2002	South America	Brazil	Case - control study	1997	1998	487	22	School children (students of nurseries and public schools	Children	Range: 2 to 9 years	255 (52,4%) women	IgG	Abbott Laboratories	IgG-C

										Amazonian region)						
Atiq, M [22].	Emerg Infect Dis	2009	North America	United States	Observational cross-sectional study	1995	2006	38	4	Healthy controls	General population	Mean age (SD): 39.6 (10.9)	100 (59.9%) men	IgG	MP Biomedicals	IgG-GP
								38	0					IgM		IgMT-GP
								129	45	Persons with chronic liver disease	Immunodeficiency			IgG		IgG-I
								129	0					IgM		IgMT-I
Bangueses, F et al [23].	J Med Virol	2020	South America	Uruguay	Survey	2017	2018	400	40	Blood donors	Blood donors	-	-	Total anti-HEV	Dia.Pro Diagnostic	Tanti-BD
								40	19					IgM		IgM(+)-BD
								40	3					RNA	Nested PCR	RNA(+)-BD
Baptista-González, H et al [24].	J Med Virol	2017	North America	Mexico	Cross-sectional study	2014	2015	127	1	Women with high-risk pregnancy	Pregnant women	Mean age: 29 years Range: 16 to 42 years	NA	IgG	Euroimmun	IgG-PW
								97	9	Asymptomatic women with serological reactivity against HCV or Hepatitis B surface antigen (HBsAg) detected during blood donation screening	Viral hepatitis	-	NA			IgG-VH
								94		Clinical symptoms suggestive of HEV infection	Viral hepatitis	-	-			
								110	0	Healthy blood donors	Blood donors	-	-			IgG-BD
Bartoloni, A et al [25].	Trop Med Int Health	1999	South America	Bolivia	Cross-sectional study	Nov-97	Nov-97	490	36	The study populations consisted of 295 individuals from Camiri and 292 individuals from Villa Montes.	Rural population	Range: 1 to 85 years	Camiri: 162 women, 133 men Villa Montes: 147 women, 145 men	IgG	Abbott Laboratories	IgG-R
Bernal Reyes, R et al [26].	Rev Gastroenterol Mex	1996	North America	Mexico	-	-	-	363	23	General population	General population	-	-	IgG	Abbott Laboratories	IgG-GP

Betancur, CA et al [27].	Acta méd. colomb	2013	South America	Colombia	Descriptive study	Jul-11	Jan-12	98	11	Pig farmers	Pig related exposure	Range: 20 to 62 years Mean age in positives: 41.7 Mean age negatives: 35	9 women 89 men	IgG	Dia.Pro Diagnostic	IgG-PRE
Bezerra, LA et al [28].	Acta Trop	2019	South America	Brazil	Cross-sectional analytical study	2016	2017	366	15	People living with HIV/AIDS (PLHA) receiving antiretroviral therapy (ART)	Immunodeficiency	Positive cases: Mean age (SD): 47.2 (14.44).	Positive cases: 12 (80%) men	IgG and RNA	RecomWell, Mikrogen	IgG-I
									0					RNA	RT-PCR	RNAT-I
Blitz-Dorfman, L et al [29].	Ann Trop Med Parasitol	1996	South America	Venezuela	-	-	-	463	45	Yukpa-Aroy	Ethnic groups	-	-	Total anti-HEV	Abbott Laboratories	Tanti-EG
										Yukpa-Marewa						
										Yukpa-Maracaibo						
										Barí-Several						
Bortoliero, AL et al [30].	Rev. Inst. Med. trop. S. Paulo	2006	South America	Brazil	Cross-sectional study	May-99	Dec-99	996	23	Volunteer blood donors	Blood donors	Mean age (SD): 29.9 (9.8) Median: 26.9 Range: 18 to 60 years	605 (60.7%) men	IgG	Abbott Laboratories	IgG-BD
Brahm, J et al [31].	Rev Med Chil	1996	South America	Chile	-	1994	1994	40	1	Alcoholics	Exposed population	-	-	IgG	Abbott Laboratories	IgG-EP
								174	7	Blood donors	Blood donors					IgG-BD
								40	3	Hemophiliacs younger than 15 years	Immunodeficiency					IgG-I
								66	6	Subjects with acute hepatitis A	Viral hepatitis					IgG-VH
								36		Subjects with acute non A-non B-non C hepatitis	Viral hepatitis					
Bricks, G et al [32].	Braz J Infect Dis	2018	South America	Brazil	Cross-sectional study	Oct-15	Dec-16	618	63	Patients chronically infected with HCV	Immunodeficiency	Mean age (Min-Max): 53.8 (22-86)	53% men	IgG and IgM	Wantai Biological Pharmacy Enterprise	Excluded Duplicate database (reference 33)
Bricks, G et al [33].	Braz J Infect Dis	2019	South America	Brazil	Cross-sectional study	Oct-15	Dec-16	618	63	Patients chronically infected with hepatitis C virus	Immunodeficiency	Mean age (Min-Max): 53.8 (22-86)	53% men	IgG	Wantai Biological Pharmacy Enterprise	IgG-I
								66	0					IgM		IgM(+)-I

Cabezas, C et al [34].	Plos One	2020	South America	Peru	Cross-sectional, population-based study	Dec-14	Jun-15	5183	729	General Population	General Population	Range: 15–69 years. 54.3% were aged between 30 and 65 years	71.5% women	IgG	Wantai Biological Pharmacy Enterprise	IgG-GP
Caetano, K et al [35].	Trans R Soc Trop Med Hyg	2020	South America	Brazil	Cross-sectional study	2011	2012	920	36	Rural settlement projects in the states of Goiás and Mato Grosso do Sul	Rural population	>19 years (74.3%)	51% women	Total anti-HEV	RecomWell, Mikrogen	Tanti-R
Campbell, C [36].	University of Minnesota (Thesis)	2019	North America	United States	Seroprevalence	2009	2016	18695	-	NHANES study	General population	-	-	IgG	In House / Wantai / Western Blot / DS	IgG-GP
														IgM	In House / Wantai / Western Blot / DS	IgMT-GP
Campolmi, I et al [37].	Am J Trop Med Hyg	2018	South America	Bolivia	Cross-sectional study	Nov-13	Nov-13	257	87	Healthy volunteers from two rural communities	Rural population	Median age (IQR): 27 (12–46) Range: 1 to 87 years	131 (51%) women	IgG	Dia.Pro Diagnostic	IgG-R-Diapro We included borderline in the sample
									77						Wantai Biological Pharmacy Enterprise	Excluded Sensitive analysis
Cangin, C et al [38].	J Med Virol	2019	North America	United States	Survey	2013	2014	7656	-	Persons in the National Health and Nutrition Examination Survey [NHANES] 2013-2014	General population	Mean age of tested youth: 12.2 years Mean age of adults tested for HEV: 49.1 years The median age (IQR) of tested adults: 48 (34-63)	52.3% women	IgG and IgM	Diagnostic Systems	Excluded Duplicate database-NHANES
						2015	2016	7124	-	Persons in the National Health and Nutrition Examination Survey [NHANES] 2015-2016		Mean age of tested underage persons: 12.1 years. Mean age of adults tested	51.9% women			

												for HEV: 49.6 years Median age (IQR) of tested adults: 49 (34-64)				
Cordova, C et al [39].	Rev. bras. anal. Clin	2007	South America	Brazil	-	Mar-04	Jun-04	20	0	Pregnant	Pregnant women	-	NA	IgG	Genelabs Diagnostics	IgG-PW
									1					IgM		IgMT-PW
								20	1	Women blood donors	Blood donors			IgG		IgG-BD
									2					IgM		IgMT-BD
Cossaboom, CM et al [40].	J Med Virol	2016	North America	United States	-	2002	2009	335	21	Undergraduate and veterinary students at a major university	Occupational group	Median age: 23 years Range: 19 to 45 years	267 women 68 men	Total anti-HEV	Wantai Biological Pharmacy Enterprise	Tanti-OG
Covarrubías, N et al [41].	Rev. chil. Infectol	2018	South America	Chile	Seroprevalence study	Mar-14	Apr-14	186	56	Blood donors	Blood donors	Mean age: 37 Range: 18 to 68 years	80 (43%) women 106 (57%) men	IgG	Diagnostic Automation Inc	IgG-BD
Covarrubías, N et al [42].	Rev. chil. infectol.	2015	South America	Chile	Reevaluated retrospectively	2009	2012	178	58	Patients with previous analysis of anti-VHE IgG antibodies in the Laboratorio de Gastroenterología del Hospital Clínico de la Universidad de Chile	Viral hepatitis	Mean age: 40 Range: 2 to 86 years	90 women 88 men	IgG	Diagnostic Automation Inc	IgG-VH
									13						Genelabs Diagnostics	Excluded Sensitive analysis
Cruells, MR et al [43].	Gastroenterol Hepatol	1997	South America	Uruguay	Epidemiological study	-	-	252	5	Blood donors from the National Blood Service	Blood donors	-	-	Total anti-HEV	Abbott Laboratories	Tanti-BD
								214	6	Patients who consulted in the Policlínica de Nutrición y Digestivo were studied	General population					Tanti-GP

de Almeida, E. Araujo DC et al [44].	Int J Infect Dis	2020	South America	Brazil	Cross-sectional survey	2011	2013	169	37	Urban residents (18-year or older) during visits to the local health center and routine home visits	General population	-	155 women 93 men	IgG	Wantai Biological Pharmacy Enterprise	IgG-GP
								73	13	Rural residents (18-year or older) during visits to the local health center and routine home visits	Rural					IgG-R
								244	0	Rural and Urban residents	Combined			RNA	RT-PCR	RNAT-Comb
de la Caridad Montalvo Villalba, M et al [45].	Infect Genet Evol	2013	Central America and the Caribbean	Cuba	Survey	Feb-07	May-07	69	28	Employees with swine related occupations	Pig related exposure	-	-	Total anti-HEV	Genelabs Diagnostics	Tanti-PRE
								37	10	Workers without contact with pigs.	Occupational group	-	-	Total anti-HEV	Genelabs Diagnostics	Tanti-OG
								38	1	Persons working in swine farms	Combined	-	-	RNA	nRT-PCR	RNA(+)-Comb
de la Caridad Montalvo Villalba, M et al [46].	Rev Biomed	2016	Central America and the Caribbean	Cuba	Descriptive study	2013	2013	422	36	Patients with suspect of acute viral hepatitis	Viral hepatitis	-	-	RNA	RT-PCR	RNAT-VH
de la Caridad Montalvo Villalba, M et al [47].	Trans R Soc Trop Med Hyg	2010	Central America and the Caribbean	Cuba	Cross-sectional study	May-03	Aug-03	469	47	Healthy individuals	General population	Mean age (SD): 31.9 (14.3) Range: 5 to 60 years	267 (56.9%) women 202 (43.1%) men	IgG	Genelabs Diagnostics	IgG-GP
de la Caridad Montalvo Villalba, M et al [48].	Emerg Infect Dis	2008	Central America and the Caribbean	Cuba	Outbreak investigation	1999	2005	31	22	Outbreaks and sporadic cases. The first outbreak occurred in 1999 in a factory; 20 persons were affected. The second	Viral hepatitis	The first outbreak: median age 45 years, range 22–53 years. The second outbreak: median age 24 years, range 17–45 year	The first outbreak: 12 women, 8 men The second outbreak: 15	IgG	Genelabs Diagnostics	IgG-VH
								20+26+12	58					IgM		IgMT-VH Note: We assumed 58 as all cases
								22	2					RNA	PCR	RNA(+)-VH

										outbreak was in 2005 in a suburb of Havana and involved 26 persons. 12 sporadic clinical cases			women, 11 men			
de Oliveira, Jmns et al [49].	Int J Infect Dis	2018	South America	Brazil	Cohort study	2014	2014	316	8	Kidney transplant recipients	Immunodeficiency	Mean (SD): 46.4 (12.3)	174 (55.1) men	IgG	RecomWell, Mikrogen	IgG-I
									1					IgM		IgMT-I
									0					RNA	RT-PCR	RNAT-I
de Paula, VS et al [50].	Mem Inst Oswaldo Cruz	2001	South America	Brazil	-	1997	1997	349	14	Tributaries located in the States of Acre and Amazonas	Ethnic groups	Mean age: 19.4 Range: 3 to 73 years	53.2% men	IgG	Abbott Laboratories	IgG-EG
Debes, JD et al [51].	PLoS One	2016	South America	Argentina	-	2010	2010	433	-	Control group of HIV-negative individuals	General population	-	-	-	-	Excluded same control group for another publication (reference 94)
								204	15	HIV-infected individuals	Immunodeficiency	Mean age of HEV-positive: 44.9 Mean age of HEV-negative: 39.7 Median age HEV-positive (IQR): 39 (35–41)		IgG	Dia.Pro Diagnostic	IgG-I
								15	5					IgM		IgM(+)-I
								204	1					RNA	Nested PCR	RNAT-I
Dell'Amico, MC et al [52].	Emerg Infect Dis	2011	South America	Bolivia	Survey	Nov-06	Dec-06	236	15	Persons in 2 rural communities (172 in Bartolo and 64 in Casas Viejas)	Rural population	Range: 1 to 87 years	-	IgG	Dia.Pro Diagnostic	IgG-R
								122	4					RNA	RT-PCR	Excluded fecal samples
Di Lello, FA et al [53].	Eur J Gastroenterol Hepatol	2020	South America	Argentina	Seroprevalence study	Feb-19	May-19	391	44	Blood donors	Blood donors	Median (IQR): 36 (28-44)	196 (50.1%) women 195 (49.9%) men	IgG	Dia.Pro Diagnostic	IgG-BD
								44	8					IgM		IgM(+)-BD
								44	0					RNA	Cobas 4800 System	RNA(+)-BD

Diehl, TM et al [54].	Gastroenterol Res Pract	2018	North America	United States	Cross-sectional study	2009	2012	14951	-	National Health and Nutrition Examination Survey (NHANES). The NHANES sample is a stratified multistage probability cluster designed to represent the total civilian noninstitutionalized US population	General population	≥6 years	-	IgG and IgM	Diagnostics System	Excluded Duplicate database-NHANES
Ditah, I et al [55].	Hepatology	2014	North America	United States	Survey	2009	2010	7885	-	The NHANES is conducted by the National Center for Health Statistics (NCHS) of the Centers for Disease Control and Prevention (CDC). It collates nationally representative data on the health and nutritional status of the noninstitutionalized, civilian population of the U.S.	General population	Median age (IQR): 37 (17-58)	51.2% women	IgG and IgM	Diagnostics System	Excluded Duplicate database-NHANES
Eick, A et al [56].	J Infect Dis	2010	North America	United States	Cohort study	Jan-02	Dec-06	1500	16	United States (US) service members who were deployed to Afghanistan	Occupational group	-	1354 (90.3%) men	Total anti-HEV	In house	Tanti-OG
								16	0					IgM		IgM(+)-OG
Engle, RE et al [57].	J Clin Microbiol	2002	North America	United States	-	-	-	230	31	U.S. blood bank volunteers	Blood donors	-	-	IgG	Sar-55 (genotype 1)	Excluded (reference 58 cited as
									35						Meng	

															(genotype 3)	data source)
								603	58	U.S. pig handlers	Pig related exposure				Meng (genotype 3)	Excluded Sensitive analysis
									63						Sar-55 (genotype 1)	IgG-PRE
Fainboim, H et al [58].	J Viral Hepat	1999	South America	Argentina	Prospective study	Jul-94	May-95	91	6.6%	Anti-human immunodeficiency virus (HIV)-positive population	Immunodeficiency	Median age: 29 years Range: 16 to 67 years	125 women 359 men	IgG	Abbott Laboratories	Excluded Unclear denominator
								1500	27	Blood donors	Blood donors	-	-			IgG-BD
Fantilli, AC et al [59].	PLoS One	2019	South America	Argentina	Cross-sectional study	2017	2018	140	35	Individuals with cirrhosis	Immunodeficiency	Mean age: 61 years. Range: 23 to 88 years	The male-to-female ratio was 1.8/1.	IgG	Dia.Pro Diagnostic	IgG-I
									3					RNA	Nested-PCR	RNAT-I
								35	16					IgM	Dia.Pro Diagnostic	IgM(+)-I
								72	9	Patients with alcohol use disorder (AUD, without cirrhosis)	Exposed population	Median age: 51 years. Range: 27 to 67 years	The male-to-female ratio was 8/1.	IgG	Dia.Pro Diagnostic	IgG-EP
								9	1					IgM		IgM(+)-EP
								300	12	Healthy Controls	General population	Median age: 35 years. Range: 20 to 78 years	The male-to-female ratio was 0.3/1.	IgG	Dia.Pro Diagnostic	IgG-GP
Fearon, MA et al [60].	Transfusion	2017	North America	Canada	-	Jul-13	Dec-15	4102	241	Blood donors	Blood donors	-	-	IgG	Wantai Biological Pharmacy Enterprise	IgG-BD
								13993	0					RNA	PCR	RNAT-BD
Ferreira, AC et al [61].	Arch Virol	2018	South America	Brazil	-	-	-	354	38	HIV-infected patients	Immunodeficiency	Median age: 48 Range: 19 to 76 years	71.5% men	IgG	RecomWell, Mikrogen,	IgG-I
									5					IgM		IgMT-I
									0					RNA	qRT-PCR	RNAT-I
Focaccia, R et al [62].	Braz. j. infect. Dis	1998	South America	Brazil	Seroprevalence study	Feb-96	May-96	1012	18	Individuals older than 2 years	General population	≥ 2 years	-	Total Anti-HEV	Abbott Laboratories	Tanti-GP
Fontana, RJ et al [63].	Hepatology	2016	North America	United States	-	Jan-98	Jul-11	681	294+3	U.S. Acute Liver Failure Study Group	Immunodeficiency	Mean age: 41.8 years	32.9% men	IgG	In house	IgG-I
								699	3					IgM	Wantai Biological Pharmacy Enterprise	IgMT-I
								3	0					RNA	RT-PCR	RNA(+)-I

Freitas, NR et al [64].	Mem Inst Oswaldo Cruz	2016	South America	Brazil	Cross-sectional study	Apr-12	Oct-14	379	22	Patients with acute non-A, non-B, non-C hepatitis	Viral hepatitis	Mean age (SD): 36.9 (17.2)	56.5% women	IgG	RecomWell, Mikrogen,	IgG-VH
									1					IgM		IgMT-VH
								22	0					RNA	Roche Diagnostics	RNA(+)-VH
Freitas, NR, et al [65].	Rev Soc Bras Med Trop	2017	South America	Brazil	Cross-sectional study	May-11	Jul-11	464	19	Rural settlements in the southwest of the State of Goiás	Rural population	Mean age (SD): 37.7 (19.9)	52.2% men	IgG	RecomWell, Mikrogen	IgG-R
									16						RecomLine, Mikrogen after a positivenRecomWell, Mikrogen	Excluded Sensitive analysis
									3					IgM	RecomWell, Mikrogen	IgMT-R
									0						RecomLine, Mikrogen, after a positivenRecomWell, Mikrogen	Excluded Sensitive analysis
									0					RNA	RT-PCR	RNAT-R
Gambel, JM et al [66].	Am J Trop Med Hyg	1998	-	Haiti	Survey	1995	1995	105	3	Haitian civilian participants	General population	Mean age: 33.8 years	27 (26%) women	Total Anti-HEV	In house	Tanti-GP
				Guatemala				111	6	Guatemala soldiers	Occupational group	≤ 25 years	-			Tanti-OG
				Honduras				109	6	Honduras soldiers	Occupational group	-	-			Tanti-OG
				USA				109	2	USA soldiers	Occupational group	≤ 25 years	96% men			Tanti-OG
Gandolfo, GM et al [67].	Med Clin (Barc)	2003	South America	Bolivia	-	-	-	435	4	Children from two different schools: one attended by children belonging to a high social class of the town (group A), and the other school attended by children belonging to the poorest social class (group B).	Children	Range: 6 to 18 years	53.5% women 46.5% men	Total Anti-HEV	Abbott Laboratories	Tanti-C
Garcia, CG et al [68].	J Med Virol	2012	South America	Venezuela	-	Jan-08	Dec-08	74	22	Patients with clinical features	Viral hepatitis	Mean age: 32 Range: 1 to 55 years	27 women 47 men	IgM	DRG International	IgMT-VH

								22	3	suggestive of acute viral hepatitis				RNA	Nested RT-PCT	RNA(+)-VH
Goncales, NS et al [69].	Clin Diagn Lab Immunol	2000	South America	Brazil	Seroprevalence study	-	-	170	10	Hospital employees	Occupational group	Mean age (SD) 33.2 (8.2), range 20 to 53 years, median 31 years	135 (71.5%) women, 35 (20.5%) men	IgG	Abbott Laboratories	IgG-OG
								205	8	Volunteer blood donors (165 volunteer blood donors with alanine aminotransferase (ALT) levels ↕2 times the upper normal value (129 [78.2%] and 43 volunteer blood donors with ALT levels ¬2 times the upper normal value)	Blood donors	group IA: mean age (SD) 33.9 (10.1), range 18 to 61 years, median 32 years group IB: mean age (SD) 34.3 (7.9), range 21 to 54 years, median 34 years	group IA: 36 (21.8%) women, 129 (78.2%) men group IB: 4 [10%] women, 39 (90%) men			IgG-BD
								214	38	Women who attended a center for anonymous testing for human immunodeficiency virus (HIV) infection	Exposed population	Mean age (SD) 29.6 (10.2), range 14 to 71 years, median 26 years	NA			IgG-EP
Gutiérrez-Vergara, CC et al [70].	Iatreia	2015	South America	Colombia	Cross-sectional study	-	-	34	2	Cohabitant people of the exposed population	Exposed population	Mean age (SD): 42 (14) Median (Q1-Q3): 38 (34-51)	28 women 6 men	Total anti-HEV	Dia.Pro Diagnostic	Tanti-EP
								3	0					IgM		IgM(+)-EP
								983	71	General population	General population	Mean age (SD): 44 (15) Median (Q1-Q3): 44 (32-55)	791 women 192 men	Total anti-HEV	Dia.Pro Diagnostic	Tanti-GP
								79	8					IgM		IgM(+)-GP
								159	25			Mean age (SD): 35 (11)	29 women	Total anti-HEV	Dia.Pro Diagnostic	Tanti-PRE

								31	4	People exposed to pigs	Pig related exposure	Median (Q1-Q3): 33 (27-42)	130 men	IgM		IgM(+)-PRE
								13	0					RNA	RT-PCR	Excluded fecal samples
Guzmán Rojas, P et al. [71]	Diagnóstico (Perú)	2013	South America	Peru	Cross-sectional study	-	-	107	30	Slaughterhouses workers	Pig related exposure	Mean age (SD): 35 (11.93) Range: 18 to 67 years	92% men	IgG	In house	IgG-PRE
Halac, U et al [72].	Gut	2012	North America	Canada	-	1992	2010	14	4	Children with orthotropic liver transplantation (OLT) : group 2: with persistently increased serum aminotransferases and histological features of chronic hepatitis (cases)	Viral Hepatitis	Median age: 17.4 years Range: 5.9 to 19.8 years	8 women	IgG	In house	IgG-VH
								66	10	Children with orthotropic liver transplantation (OLT): group 1: with normal serum aminotransferases (control)	Immunodeficiency	Median age: 13.7 years Range: 1.8 to 25.5	37 women	IgG	In house	Excluded follow up samples
									0					IgM		Excluded follow up samples
Hardtke, S et al [73].	J Med Virol	2018	South America	Brazil	Cohort study	2002	2003	199	51	Female blood donors	Blood donors	Mean age (SD): 29.3 (7.7)	NA	IgG	Wantai Biological Pharmacy Enterprise	IgG-BD
								209	40	Pregnant women	Pregnant women	Mean age (SD): 28.7 (6.6)				IgG-PW
								51	0	Female blood donors	Blood donors	Mean age (SD): 29.3 (7.7)		RNA	In house PCR	RNA(+)-BD
								40	0	Pregnant women	Pregnant women	Mean age (SD): 28.7 (6.6)				RNA(+)-PW
Hering, T et al [74].	J Med Virol	2014	South America	Brazil	-	Jan-11	Oct-11	192	28		Immunodeficiency			IgG	RecomWell, Mikrogen	IgG-I

									20	Renal transplant patients		Mean age (SD): 42.6 (11.9)	124 (65%) men	RNA	Nested RT-PCR	RNAT-I
Hurtado, C et al [75].	Rev Med Chil	2005	South America	Chile	-	-	-	35	12	Positive serum samples for IgG antibodies against hepatitis virus E	Viral hepatitis	-	-	IgM	Genelabs Diagnostics	IgM(+)-VH
								25	5	Samples of HAV acute cases (HAV IgM+)						IgMT-VH
Hyams, KC et al [76].	Clin Infect Dis	1996	South America	Peru	-	1989	1989	179	25	Healthy male troops	Occupational group	Age range: 18 to 25 years	NA	IgG	Diagnostics Biotechnology	IgG-OG
								158	24	Patients with acute jaundice	Viral hepatitis	Age range: 10 to 83 years	-			IgG-VH
								24	4					IgM	In house	IgM(+)-VH
Ibarra V, H et al [77].	Rev Med Chil	2006	South America	Chile	Cohort study	Apr-99	Dec-00	168	2	Children for Programa de Control de Niño Sano	Children	4	71 (42.3%) women 97 (57.7%) men	IgG	Abbott Laboratories	IgG-C
Ibarra, H et al [78].	Rev Med Chil	1997	South America	Chile	Prospective study	1994	1994	100	17	Araucanian Indians	Ethnic groups	Mean age: 38.6	59 women 41 men	Total Anti-HEV	Abbott Laboratories	Tanti-EG
								1360	109	Blood donors	Blood donors	Valdivia: Mean age: 32.7 Osorno: Mean age: 30.8 Puerto Montt: Mean age: 31	Valdivia: 240 women, 697 men Osorno: 60 women, 171 men Puerto Montt: 48 women, 144 men			Tanti-BD
								72	9	Health care workers	Occupational group	Mean age: 39.3	70 women 2 males			Tanti-OG
								241	18	Inmates in state jails	Exposed population	Mean age: 26.6	5 women 236 men			Tanti-EP
Ibarra, H et al [79].	Rev Med Chil	2001	South America	Chile	Cross-sectional study	Jan-96	Sep-98	59	4	Patients with a clinical acute hepatitis	Viral hepatitis	Mean age: 27.1 Range: 15 to 58 years old	30 women 29 men	IgG	Abbott Laboratories	IgG-VH
									1					IgM		IgMT-VH
Karetnyi, YV et al [80].	J Clin Virol	1999	North America	United States	-	Jan-97	Oct-97	204	10	Patients with non-A, non-B,	Viral hepatitis	-	-	IgG	In house	IgG-VH
								10	5					IgM		IgM(+)-VH

								5	0	non-C hepatitis (non-A-C);				RNA	RT-PCR	RNA(+)-VH
						1997	1997	87	5	Staff members of the Department of Natural Resources (DRN)	Occupational group			IgG	In house	IgG-OG
								5	1					IgM		IgM(+)-OG
								1	0					RNA	RT-PCR	RNA(+)-OG
						1989	1989	332	11	Volunteer blood donors in 1989	Blood donors			IgG	In house	IgG-BD
						1998	1998	111		Volunteer blood donors in 1998						
						-	-	11	3	-				IgM		IgM(+)-BD
						-	-	3	0	-				RNA	RT-PCR	RNA(+)-BD
Kiesslich, D et al [81].	Trans R Soc Trop Med Hyg	2002	South America	Brazil	-	-	-	192	1	Hemodialysis patients	Immunodeficiency	Range: 14 to 87 years	76 women 116 men	Total Anti-HEV	Abbott Laboratories	Tanti-I
								100	0	Pregnant women	Pregnant women		NA			Tanti-PW
								227	1	Voluntary blood donors	Blood donors		43 women 184 men			Tanti-BD
Koning, L et al [82].	BMC Infect Dis	2015	North America	United States	Follow-up study	1997	2010	145	53	Patients who underwent liver transplantation for chronic Hepatitis C	Immunodeficiency	Positive: Mean age (SD): 53 (7) Negative: Mean age (SD): 51 (8)	Positive: 45 (85%) men Negative: 64 (70%) men	IgG	Wantai Biological Pharmacy Enterprise	IgG-I
									5					IgM		IgMT-I
									0					RNA	qRT-PCR	RNAT-I
Konomi, N et al [83].	J Clin Microbiol	1999	South America	Bolivia	-	1992	1998	574	93	Healthy blood donors	Blood donors	Range: 17 to 56 years	The male/female ratio was 2.5:1	IgG	In house	IgG-BD
									10					IgM		IgMT-BD
Kuniholm, MH et al [84].	Hepatology	2016	North America	United States	-	Apr-84	Mar-13	4	3	HIV+ people	Immunodeficiency	-	-	IgG	Wantai Biological Pharmacy Enterprise	Excluded only RNA positive
								4	1					IgM		
								1797	3					RNA	Procleix HEV assay and retest using TMA	RNAT-I

Kuniholm, MH et al [85].	J Infect Dis	2009	North America	United States	Cross-sectional study	1988	1994	18695	-	Serum samples collected in the Third National Health and Nutrition Examination Survey (NHANES III)	General population	≥6 years	10124 women 8571 men	IgG	In house	Excluded Duplicate database-NHANES
Kyvernitakis, A et al [86].	Hepatol Res	2015	North America	United States	Prospective observational study	Nov-12	Jul-14	115	13	HCV-infected cancer patients	Immunodeficiency	HEV IgG positive: mean age 66, range 52 to 77 years HEV IgG negative: mean age 60, range 31 to 87 years	HEV IgG positive: 9 (69%) men HEV IgG negative: 71 (70%) men	IgG	RecomWell, Mikrogen	IgG-I
								13	0					IgM		IgM(+)-I
Langer, B. C et al [87].	J Viral Hepat	1997	North America	Greenland	-	1994	1994	503	15	Inuits	Ethnic groups	Mean age: 35 years Range: 7-79 years	317 women 186 men	IgG	Abbott Laboratories	IgG-EG
									1					IgM	In house	IgMT-EG
									0					RNA	PCR	RNAT-EG
Lemos, G et al [88].	J Clin Virol	2000	Central America and the Caribbean	Cuba	-	-	-	461	6	Anti-HCV positive & HBsAg positive	Viral hepatitis	-	-	IgG	In house	IgG-VH
								6	1					IgM	In house	IgM(+)-VH
								146	24	Patients with sporadic acute viral hepatitis				IgM	In house and Genelabs Diagnostics	IgMT-VH
								1149	16	Healthy blood donors	Blood donors			IgG	In house	IgG-BD
								16	5					IgM	In house and Genelabs Diagnostics	IgM(+)-BD
								242	6	Elevated ALAT & Plasmapheresis	Immunodeficiency			IgG	In house	IgG-I
								6	6					IgM	In house and Genelabs Diagnostics	IgM(+)-I
León, P et al [89].		1999	South America	Bolivia		1992	1996	318	64	Amazonian population	Ethnic groups	-	-	IgG		IgG-EG

	Rev Panam Salud Publica				Seroepidemiological study			185	36	Rural Andina population	Rural				Abbott Laboratories	IgG-R
								98	94	Homeless children	Exposed population					IgG-EP
								95		Sexual workers			NA			
Lopes Dos Santos, DR et al [90].	J Clin Virol	2010	South America	Brazil	Retrospective study	2004	2008	64	1	Patients with acute non-A-C hepatitis	Viral hepatitis	-	-	IgG	Biokit SL	IgG-VH
									1					IgM		IgMT-VH
								1	1					RNA	RT-PCR	RNA(+)-VH
Lopez-Santaella, T et al [91].	Ann Hepatol	2020	North America	Mexico	Longitudinal/transversal clinical and virologic study	2012	2014	99	3	Pediatric patients	Immunodeficiency	-	42 women 57 men	IgG	LifeSpan BioSciences	IgG-I
									6					IgM		IgMT-I
									54					RNA	RT-PCR	RNAT-I
Lyra, AC et al [92].	Braz J Med Biol Res	2005	South America	Brazil	-	1995	1999	94	21	Patients with acute viral hepatitis A, B and non-A-C	Viral hepatitis	Mean age (SD): 24.50 (15.58))	35 women 59 men	IgG	Abbott Laboratories	IgG-VH
								21	5					IgM	Genelabs	IgM(+)-VH
Mahajan, R et al [93].	Emerg Infect Dis	2013	North America	United States	-	Mar-09	Jun-10	508	14	Persons who inject drugs	Exposed population	Mean age: 29 years Range: 18 to 40 years	72% men	IgG	Diagnostics System	IgG-EP
								14	0					IgM		IgM(+)-EP
								14	0					RNA	PCR	RNA(+)-EP
Martinez Wassaf, MG et al [94].	J Clin Virol	2014	South America	Argentina	Retrospective study	Sep-09	Sep-10	433	19	Healthy adult population	General population	Range: 18 to 78 years	-	IgG	Dia.Pro Diagnostic	IgG-GP
Martinez, AP et al [95].	J Med Virol	2021	South America	Argentina	Retrospective cross-sectional study	Jan-16	Dec-18	412	7	Young population under 18 years old, from rural areas of Chaco Province	Children	Median age (IQR): 14 (12-16)	209 (50.7%) women	IgG	Dia.Pro Diagnostic	IgG-C
								7	0					IgM		IgM(+)-C
Martins, RM et al [96].	J Clin Virol	2014	South America	Brazil	Cross-sectional study	Apr-10	May-11	431	24	Population of recyclable waste pickers	Occupational group	Mean age (SD): 36.9 (13.6)	269 (62.4%) women 162 (37.6%) men	IgG	RecomWell, Mikrogen	IgG-OG
									22						Recomline, Mikrogen after a positive RecomWell, Mikrogen	Excluded Sensitivity analysis
									4					IgM	RecomWell, Mikrogen	IgMT-OG
									3						Recomline, Mikrogen after a positive RecomWell, Mikrogen	Excluded Sensitivity analysis

								4	0					RNA	Nested RT-PCR	RNA(+)-OG
Mast, EE [97].	J Infect Dis	1997	North America	United States	Case-control study	Nov-93	Mar-94	5000	59	Blood donors	Blood donors	Respondents: mean age 42 years Non-respondents: mean age, 40 years	Respondents: 55% men Non-respondents: 54% men	anti-HEV	In house (MPr-EIA)	Excluded Sensitivity analysis
								5000	70						In house (RPr-EIA)	Tanti-BD
McGivern, DR et al [98].	Open Forum Infect Dis	2019	North America	Canada & United States	Cohort study	2016	2016	600	171	Persons with chronic HBV in the Hepatitis B Research Network Cohort Study	Immunodeficiency	Median age (IQR): 42.1 (32.8-53.0)	304 (50.7%) women 296 (49.3) men	IgG	Wantai Biological Pharmacy Enterprise	IgG-I
									10					IgM		IgMT-I
									0					RNA	RT-PCR	RNAT-I
Meng, XJ et al [99].	J Clin Microbiol	2002	North America	United States	-	1999	1999	400	73	Blood donors	Blood donors	-	-	IgG	In house (Sar-55)	IgG-BD
								400	66						In house (Swine HEV)	Excluded Sensitivity analysis
								388	93	Veterinarians working with swine	Pig related exposure				In house (Sar-55)	IgG-PRE
								388	83						In house (Swine HEV)	Excluded Sensitivity analysis
Miernyk, KM et al [100].	Vector Borne Zoonotic Dis	2019	North America	United States	Seroprevalence study	2007	2008	77	-	Avian wildlife biologists	Occupational group	Age ranged from 5 - 85 years	25 (32%) women	IgG	Diagnostics System	Excluded No crude number
								160		Sport bird hunters			11 (7%) women			
								196	-	Persons with no wild bird exposure	General Population		139 (71%) women			
								233	-	Subsistence bird hunters Alaska Native and lived in rural communities	Ethic groups		21 (9%) women			
								221		Subsistence family members Alaska Native and lived in rural communities			187 (85%) women			
Minuk, GY et al [101].	Can J Gastroenterol	2007	North America	Canada	-	1980	1980	393	11	Indigenous North American	Ethnic groups	IgG Positive: Mean age (SD) 29 (8)	IgG Positive: 3 men	IgG	Genelabs Diagnostics	IgG-EG
								11	2					IgM		IgM(+)-EG

								11	0	population (Canadian Inuit community)		IgG Negative: Mean age (SD) 22 (1)	IgG Negative: 194 (51%) men	RNA	RT-PCR	RNA(+)-EG
Moraes dos Santos, DC et al [102].	Mem Inst Oswaldo Cruz	2002	South America	Brazil	-	1999	1999	699	17	Residents from Manguinhos community assisted at the Health Unit Sinval Germano Faria/ National School of Public Health (ENSP-Fiocruz)	General population	-	525 women 174 men	IgG	Abbott Laboratories	IgG-GP
Munne, MS et al [103].	J Clin Virol	2011	South America	Argentina	Prospective study	Jan-05	Dec-10	155	2	Children with acute non A-C hepatitis.	Viral hepatitis	Median age: 7.3 years	55% women	IgG	Dia.pro and Abbott	Excluded unknown denominator
								15		Children with fulminant liver failure (FHF) and hepatitis A.		Median age: 7.5 years	8 women			
								76	2	Adults with acute non A-C hepatitis		Median age: 40 years Range: 18-74	52% women	IgG	Abbott Laboratories	
									6					IgM	Genelabs Diagnostics	
									5					Total Anti-HEV	Dia.Pro Diagnostic	
								15	9	Children and adults		-	-	RNA	Nested RT-PCR	RNA(+)-VH
Munne, MS et al [104].	Ann Hepatol	2014	South America	Argentina	-	Jan-11	Dec-13	24	4	Blood donors	Blood donors	Median age: 35 Range: 20 to 55 years	46% men	IgG	Wantai Biological Pharmacy Enterprise	IgG-BD
								27	4	Healthcare workers	Occupational group	Median age: 40 Range: 19 to 68 years	22% men		Wantai Biological Pharmacy Enterprise	IgG-OG
								28	10	HIV+	Immunodeficiency	Median age: 44 Range: 18 to 56 years	79% men		Wantai Biological Pharmacy Enterprise	IgG-I
								95	9	Volunteers	General Population	Median age: 50 Range: 21 to 84 years	48% men		Dia.Pro Diagnostic	IgG-GP

								28	4	Volunteers	General Population	Median age: 45 Range: 19 to 66 years	43% men		Wantai Biological Pharmacy Enterprise	IgG-GP
								143	9	Patients with acute non-A-C hepatitis	Viral Hepatitis	Median age: 45 Range: 18 to 84 years	49% men	RNA	Nested RT-PCR	RNAT-VH
Ooi, WW et al [105].	Am J Trop Med Hyg	1999	North America	United States	-	-	-	384	9	Travelers before and after going abroad	Exposed population	-	-	IgG	In house	IgG-EP
								236	0					IgM		Excluded not fully reported
Pandolfi, R et al [106].	PLoS One	2017	South America	Brazil	-	Mar-05	Oct-05	780	314	Blood donors	Blood donors	-	-	IgG	In house	IgG-BD
Panduro, A et al [107].	Salud Publica Mex	2011	North America	Mexico	Pilot study	2003	2007	311	31	Control subjects	General population	-	-	IgG	MP Diagnostics	IgG-GP
								34	23	Cirrhosis	Immunodeficiency	-	-			IgG-I
								83		Obesity and diabetes		-	-			
								160	6	Drug addicts	Exposed population	-	-			IgG-EP
Pang, L et al [108].	Am J Trop Med Hyg	1995	South America	Brazil	Community-based survey	Sep-93	Sep-93	97	6	Gold miners	Occupational group	Positive individuals: mean age: 27 years	Positive individuals: one woman	IgG	Genelabs Diagnostics	IgG-OG
Parana, R et al [109].	Am J Trop Med Hyg	1997	South America	Brazil	Seroprevalence study	1992	1994	200	4	Blood donors	Blood donors	Mean age: 39 years	19 women 181 men	IgG	Abbott Laboratories	IgG-BD
								392	3	Hemodialysis patients	Immunodeficiency	Mean age: 43 years	148 women 244 men			IgG-I
								30		Patients with hepatosplenic schistosomiasis		Mean age: 34 years	12 women 18 men			
								79	14	Patients with acute viral hepatitis (AVH)	Viral hepatitis	Mean age: 32 years	30 women 49 men			IgG-VH
Parana, R et al [110].	Hepatology	1999	South America	Brazil	Follow-up study	Aug-92	Dec-96	143	5	Patients with diagnosis of acute sporadic hepatitis	Viral hepatitis	Positive cases: Mean (SD): 28.6 (16.3) Median: 22.0	61 (43%) women 82 (57%) men	IgG	Abbott Laboratories	IgG-VH
								5	0					IgM		IgM(+)-VH
Passos, AM et al [111].	J Med Virol	2013	South America	Brazil	Retrospective study	1998	2007	3	0	Renal transplant recipients	Immunodeficiency	-	-	IgG	RecomWell, Mikrogen	Excluded another category (RNA +)

								96	3					RNA	Nested RT-PCR	RNAT-I
Passos-Castilho, AM et al [112].	Rev Soc Bras Med Trop	2015	South America	Brazil	Laboratory-based surveillance study	Jan-98	Dec-13	2271	47	Patients clinically suspected of being HEV carriers at clinics or hospitals	Viral hepatitis	Mean age (SD): 37.8 (16.0) Median age: 37 years Range: 0 to 94 years	50.5% women	IgG	In house	IgG-VH
								552	27					IgM		IgMT-VH
								6	1					RNA	RNeasy FFPE kit, Qiagen	RNA(+)-VH
Passos-Castilho, AM et al [113].	Braz J Infect Dis	2016	South America	Brazil	Cross-sectional study	-	-	80	15	Patients with Schistosoma mansoni	Immunodeficiency	Mean age (SD): 50.2 (13.7) Median age: 51 Range: 14 to 78 years	52 (65%) women	IgG	Wantai Biological Pharmacy Enterprise	IgG-I
								15	0					IgM		IgM(+)-I
								15	0					RNA	QIAgen, Hilden	RNA(+)-I
Passos-Castilho, AM et al [114].	J Med Virol	2016	South America	Brazil	Prospective, cross-sectional study	Dec-14	Dec-14	300	30	Blood donors	Blood donors	Mean age (SD): 33.2 (10.6) Median age: 31.5 Range: 18 to 64 years	188 (62.7%) men.	IgG	Wantai Biological Pharmacy Enterprise	IgG-BD
								30	1					IgM		IgM(+)-BD
								30	0					RNA	qRT-PCR	RNA(+)-BD
Passos-Castilho, AM et al [115].	Braz J Infect Dis	2017	South America	Brazil	-	Jul-14	Sep-14	500	49	Blood donors	Blood donors	Mean age (SD): 38.8 (13) Median age: 36 years old Range: 18 to 67 years	245 (49%) men	IgG	Wantai Biological Pharmacy Enterprise	IgG-BD
								49	1					IgM		IgM(+)-BD
								49	0					RNA	QIAgen, Hilden	RNA(+)-BD
Pelaez, D et al [116].	Biomedica	2016	South America	Colombia	Descriptive and retrospective study	2004	2014	1097	342	Serum from patients with positive diagnosis for viral hepatitis	Viral hepatitis	-	-	IgG	Dia.Pro Diagnostic	IgG-VH
									126					IgM		IgMT-VH
								181	52					RNA	Nested RT-PCR	RNA(+)-VH
Peláez, D et al [117].	Biomédica	2014	South America	Colombia	Retrospective study	2005	2010	344	26	Patients with clinical diagnosis of viral hepatitis	Viral hepatitis	Mean age: 25.2 Median age (IQR): 23 years (16-33)	41.8% women 54.8% men 9.8% not information	IgG	Dia.Pro Diagnostic	IgG-VH
									6					IgM		IgMT-VH
Perez, OM et al [118].	Am J Trop Med Hyg	1996	Central America and the Caribbean	Nicaragua	Cross-sectional survey	Jul-90	Mar-92	460	37	Healthy population	General population	-	-	IgG	Abbott Laboratories	IgG-GP
		2018						143	13					IgG		IgG-GP

Pisano, MB et al [119].	Trans R Soc Trop Med Hyg		South America	Argentin a	Retrospec tive, non-associated , anonymo us study	Jan-15	Jul-16	13	3	People who attended health care centres for routine control	General population	Mean age: 36 years Range: 4 to 91 years	91 women 52 men	IgM	Dia.Pro Diagnostic	IgM(+)-GP
Pisano, MB et al [120].	Arch Virol	2017	South America	Argentin a	-	-	-	120	7	Solid organ transplant recipients	Immunod eficiency	D: Mean age (SD): 60 (15.57) Range: 23 to 88 years S: Mean age (SD): 50 (15.62) Range: 18 to 77 years	D: The male-to-female ratio for patients on dialysis was 2.4/1. S: The male-to-female ratio in transplant recipients was 1.5/1.	IgG	Dia.Pro Diagnostic	IgG-I
								88	9	Patients on dialysis						
								8	5	Patients on dialysis				IgM		IgM(+)-I
								120	0	Transplant recipients				IgM		IgMT-I
								208	0	Patients on dialysis & Solid organ transplant recipients				RNA	Nested PCR	RNAT-I
Pujol, FH et al [121].	J Med Virol	1994	South America	Venezuel a	-	May -92	May -92	223	12	Amerindians (Padamo, Edo Amazonas).	Ethnic Groups	-	NA	anti-HEV	In house	Tanti-EG
						Jul-92	Jul-92	204	8	Rural populations (San Camilo, Edo Apure)	Rural population					Tanti-R
						1991	1992	184	3	Urban pregnant woman (Caracas)	Pregnant women					Tanti-PW
						-	-	21	3	Not clear population	Combined			IgM	Western blot	IgM(+)-Comb
Pujol, FH et al [122].	GEN	1994	South America	Venezuel a	Seroepide miologica l survey	1991	1992	106	3	Pregnant women: a maternity unit at the moment of delivery (106 sera, Low-income population)	Pregnant women	-	NA	anti-HEV	In house	Excluded, results from reference 121
								80		Pregnant women: private clinic during the third trimester of pregnancy						

										(105 sera, medium-high economic class population)						
Quintana, A et al [123].	J Med Virol	2005	Central America and the Caribbean	Cuba	Seroepidemiological study	-	-	209	11	General population	General population	Range: 5 to 55 years	132 women 77 men	IgG	In house	IgG-GP
Realpe-Quintero, M et al [124].	Intervirology	2018	North America	Mexico	Retrospective study	2015	2016	75	13	Pediatric Patients with a Low Socioeconomic Status with acute hepatitis	Viral hepatitis	HEV positive: Means (SD): 7.8 (3.1), Range: 1 to 16 years HEV negative: Means (SD): 7.5 (3.6), Range: 1 to 16 years	Women/ men ratio (%) RNA -: 33/29 (53.2–46.8) RNA +: 6/7 (46.1-53.9)	RNA	Nested RT-PCR	RNA(+)-VH
Redlinger, T et al [125].	Tex Med	1998	North America	United States	Cross-sectional study	Sep-95	Nov-95	557	2	Pregnant women El Paso (low income population)	Pregnant women	-	NA	Total Anti-HEV	Abbott Laboratories	Tanti-PW
				Mexico		Jun-96	Jul-96	307	5	Pregnant women Ciudad de Juarez						Tanti-PW
Remondegui, C et al [6].	Rev Argent Microbiol	2021	South America	Argentina	-	-	-	126	7	Guarani ethnic group	Ethnic groups	Mean age: 20.87 Range: 1 to 65 years	79 women 47 men Sex ratio (m/f): 0.59	IgG	RecomWell, Mikrogen	IgG-EG
									5						RecomLine, Mikrogen after a positive RecomWell, Mikrogen	Excluded Sensitivity analysis
									11					IgM	RecomWell, Mikrogen	IgMT-EG
									4						RecomLine, Mikrogen after a positive RecomWell, Mikrogen	Excluded Sensitivity analysis
								17	0					RNA	RT-PCR	RNA(+)-EG
Rendon, J et al [126].	PLoS One	2016	South America	Colombia	Prospective cross-sectional study	Apr-08	Jul-09	40	9	Patients aged over 15 with a clinical diagnosis of viral hepatitis	Viral hepatitis	Median age (IQR): 27 years (18–34.5)	17 (42.5%) women	RNA	RT-PCR	Excluded fecal samples

													23 (57.5%) men			
Rey, JA et al [127].	J Travel Med	1997	South America	Argentina	Retrospective study	Nov-94	Apr-95	2157	39	Blood donors	Blood donors	Mean age: 34.2	461 women 1696 men	IgG	Abbott Laboratories	IgG-BD
								1304	2	Children	Children	Mean age: 6.4	601 women 703 men			IgG-C
								1735	54	Patients before they were subjected to invasive procedures	Immunodeficiency	Mean age: 46.7	946 women 789 men			IgG-I
Rodríguez Lay, L et al [128].	J Med Virol	2008	Central America and the Caribbean	Cuba	-	1998	2003	258	20+33	Outbreaks cases of acute viral hepatitis	Viral hepatitis	-	-	IgM	Center for Genetic Engineering and Biotechnology	IgMT-VH
								39	13+5	Sporadic clinical cases						
Saraceni, CP [129].	Universidade de São Paulo (Thesis)	2001	South America	Brazil	Surveillance	Apr-97	Sep-99	793	7	Pregnant women	Pregnant women	Mean age: 23.8 Median age: 23 Range: 13 to 43 years	NA	IgG	In house	IgG-PW
								125	3	Suspected cases of viral hepatitis	Viral hepatitis	Mean age: 25.1 Median age: 24 Range: 1 to 78 years	63 (50,3%) women 62 (49,6%) men	IgM		IgMT-VH
Sherman, KE et al [130].	AIDS Res Hum Retroviruses	2021	North America	United States	-	2009	2017	379	71	HCV Monoinfected and HCV/HIV coinfected	Immunodeficiency	Median age: 56 Range: 21 to 82 years	76% men	IgG	Wantai Biological Pharmacy Enterprise	IgG-I
								71	0					IgM		IgM(+)-I
								379	0					RNA	RT-qPCR	RNAT-I
Sherman, KE et al [131].	J Viral Hepat	2014	North America	United States	Cohort study	2003	2003	166	32	HIV-infected kidney transplant candidates	Immunodeficiency	Median (IQRa): 46 (41-51)	38 (72%) men	IgG	Wantai Biological Pharmacy Enterprise	IgG-I
										HIV-infected liver transplant candidates		Median (IQRa): 49 (44-54)	95 (84%) men			
									1	-		-	-	IgM	Adaltis	IgMT-I
									0	-		-	-	RNA	qPCR	RNAT-I
Silva, SM et al [132].		2012	South America	Brazil	-	Jul-09	Jan-10	110	4	Blood donors	Blood donors	-	-	IgG	MP Diagnostics	IgG-BD

	Mem Inst Oswaldo Cruz							310	26	Individuals exposed to swine in the rural areas	Pig related exposure	Mean age (SD): 39.8 (18.3) Range: 11 to 98 years	51% women			IgG-PRE
Smalligan, RD et al [133].	Am J Trop Med Hyg	1995	North America	United States	-	1967	1984	328	0	Banked sera from North American missionaries	Occupational group	Mean age: 39.7 Range: 5 to 73 years	65% women	IgG	Genelabs Technologies	IgG-OG
Smith, HM et al [134].	J Infect Dis	2002	North America	United States	Seroepidemiologic study	Jun-00	Aug-00	200	27	Patients who used a free clinic in downtown (Most of these patients had been homeless during the previous year)	Exposed population	Median age: 41 years Range: 21 to 66 years	The ratio of men to women in the study was 3:1	Total Anti-HEV	In house	Tanti-EP
Souto, FJ et al [135].	Am J Trop Med Hyg	1997	South America	Brazil	-	Apr-95	Apr-95	66	7	Asymptomatic relatives	Exposed population	Mean age: 28 Range: 2 to 63 years	47 women 35 men	IgG	Abbott Laboratories	IgG-EP
								16	2	Self-reporting hepatitis cases	Viral hepatitis					IgG-VH
Souto, FJ et al [136].	Ann Trop Med Parasitol	1998	South America	Brazil	Randomized survey	-	-	299	10	General population of Cotriguacu (village with rudimentary sanitary system)	Rural population	Mean age: 25.2 Range: 2 to 74 years	149 women 150 men	IgG	Abbott Laboratories	IgG-R
Stramer, SL et al [137].	Transfusion	2016	North America	United States	-	Feb-13	Jul-13	4499	329	Blood donors	Blood donors	-	-	IgG	MP Diagnostic	IgG-BD
									26					IgM		IgMT-BD
									428					Total Anti-HEV		Tanti-BD
								18829	9					RNA	Procleix plus TMA plus RT-PCR (the last two as confirmatory, at least one positive for positivity)	RNAT-BD
Sue, PK et al [138].	Open Forum Infect Dis	2016	North America	United States	Retrospective, cross-	1988	2012	311	57	Solid Organ Transplant Recipients	Immunodeficiency	Median age: 47.5 Range: 2 to 80 years	173 women 138 men	IgG	Wantai Biological Pharmacy Enterprise	IgG-I
									2					IgM		Excluded post-transplant

					sectional study				4	(Children and adults)				RNA	qRT-PCR	Excluded post-transplant
Talarmin, A et al [139].	J Med Virol	1997	South America	French Guiana	-	Jan-92	Apr-96	996	64	Banks of sera (collected for epidemiological studies on human T-cell leukemia/ lymphoma virus type I, during routine testing of pregnant women, or for the identification of arboviruses)	Ethnic groups	Mean age (SD): 35.1 (12.5). Range: 20 to 80 years	587 women 409 men	Total Anti-HEV	Abbott Laboratories	Tanti-EG
Tejada-Strop, A et al [140].	Am J Trop Med Hyg	2019	Central America and the Caribbean	Haiti	-	2012	2012	1279	123	Pregnant Women	Pregnant women	Range: 15 to 46 years	NA	IgG	Wantai Biological Pharmacy Enterprise	IgG-PW
									4					IgM		IgMT-PW
								4	0					RNA	RT-PCR	RNA(+)-PW
Teshale, EH et al [141].	J Infect Dis	2015	North America	United States	-	1988	1994	5966	-	Noninstitutionalized civilian population	General population	≥6 years	3164 women, 2802 men	IgG	Diagnostics System	IgG-GP
						2009	2010	7885	-	Noninstitutionalized civilian population	General population	≥6 years	3979 women, 3906 men			Excluded Duplicate database-NHANES
Thomas, DL et al [142].	J Clin Microbiol	1997	North America	United States	-	-	-	300+211+300	64+29+93	Blood donors	Blood donors	-	-	IgG	In house	IgG-BD
								295	115 (47+68)	Homosexual males	Exposed population		NA			IgG-EP
								295		Injection drug users (IDUs)			-			
Tissera, G et al [143].	BMC Infect Dis	2020	South America	Argentina	Anonymous, descriptive, retrospective, non-interventionist study	2015	2018	155	4	Non-pregnant women	General population	-	NA	IgG	Dia.Pro Diagnostic	IgG-GP
								202	17	Pregnant women	Pregnant women	Median age: 30 years Range: 18 to 43 years	NA			IgG-PW
								17	2					IgM		IgM(+)-PW
								17	0					RNA	Nested RT-PCR	RNA(+)-PW
Trinta, KS et al [144].	Mem Inst Oswaldo Cruz	2001	South America	Brazil	Retrospective study	Jan-94	Dec-98	93	4	Blood donors	Blood donors	Mean age: 34.6 years	100% men	IgG	Abbott Laboratories	IgG-BD
								65	4	Hemodialysis patients	Immunodeficiency	Mean age: 65.1 years	34 (52%) women,			IgG-I

													31 (48%) men			
								145	3	Individuals living in the rural area	Rural population	Mean age: 31.3 years	69 (47.6%) women, 76 (52.4%) men			IgG-R
								260	0	Individuals living in the urban area	General population	Mean age: 10.8 years	127 (48.8%) women, 133 (51.2%) men			IgG-GP
								102	12	Intravenous drug users (IVDUs)	Exposed population	Mean age: 33.7 years	15 (14.7%) women, 88 (86.3%) men			IgG-EP
								146	3	Patients with acute Non-A Non-B Non-C (NANBNC) viral hepatitis	Viral hepatitis	Mean age: 30.6 years	69 (47.3%) women, 77 (52.7%) men			IgG-VH
								304	3	Pregnant women	Pregnant women	Mean age: 23.5 years.	NA			IgG-PW
Unzueta, A et al [145].	Ann Hepatol	2016	North America	United States	-	Mar-11	Aug -13	333	32	Patients evaluated for heart and kidney transplantation	Immunod eficiency	-	111 women, 222 men	IgG	RecomWell , Mikrogen	IgG-I
									0					RNA	In house nucleic acid amplificatio n assay (Procleix HEV assay)	RNAT-I
Vildosola, H et al [146].	Rev. gastroentero l. Perú	2000	South America	Peru	Seropreva lence study	Jul-97	Mar-98	191	20	Workers in water and sewage supply companies	Occupatio nal group	Range: 25 to 66 years	100% men	IgG	Abbott Laboratorie s	IgG-OG
Vitral, CL et al [147].	BMC Infect Dis	2014	South America	Brazil	Communi ty-based cross-sectional survey	Mar-04	Oct-04	388	50	Agricultural settlements in the Amazon Basin	Ethnic groups	Median age: 23 Range: 5 to 90 years	184 women 204 men	IgG	Biokit	IgG-EG
								43	7					IgM		IgM(+)-EG
								6	4					IgM/IgG	RecomLine , Mikrogen	Excluded confirmator y analysis of the IgM positive samples

																(one not available for further testing)
Vitral, CL et al [148].	Mem Inst Oswaldo Cruz	2005	South America	Brazil	Serological study	-	-	32	2	Pig handlers	Pig related exposure	-	-	IgG	In house	IgG-PRE
Withers, MR et al [149].	Am J Trop Med Hyg	2002	North America	United States	Survey	Aug-99	Dec-00	127	3	Non-swine worker (Control)	General population	Mean age: 43.5 Median age: 43.0 Range: 18 to 75 years	59 (46.5%) women 66 (52.0%) men	Total Anti-HEV	In house	Tanti-GP
						Aug-99	Dec-00	165	18	Swine workers	Pig related exposure	Mean age: 36.1 Median age: 35.0 Range: 18 to 64 years	30 (18.2%) Women 135 (81.8%) Men			Tanti-PRE
Xu, C et al [150].	Transfusion	2013	North America	United States	-	2006	2012	1939	364	Blood donors	Blood donors	-	-	IgG	Wantai Biological Pharmacy Enterprise	IgG-BD
									8					IgM		IgMT-BD
									0					RNA	RT-PCR and Nested PCR	RNAT-BD
Zafrullah, M et al [151].	Transfusion	2018	North America	United States	-	Mar-15	Apr-15	5040	619	Blood donors (HEV RNA negative)	Blood donors	Range: 16 to 93 years	2358 women 2682 men	IgG	Wantai Biological Pharmacy Enterprise	IgG-BD
									34					IgM		IgMT-BD
									569					IgG	Diagnostic Systems	Excluded Sensitivity analysis
									146					IgM		
									537					Total Anti-HEV	MP Biomedicals	Tanti-BD
									93					IgM		Excluded Sensitivity analysis
Zhang, L et al [152].	Hepatology	2015	North America	United States	Survey	1991	1994	1919	-	Mexican Americans	Ethnic groups	-	-	IgG	In house	Excluded Duplicate database-NHANES
								1919	-	Non-Hispanic blacks						
								2434	-	Non-Hispanic whites						

Excluded studies from the quantitative analysis are highlighted in red

5. Risk factors

Table 5. Risk factors reported by the included studies

First author	Journal	Year of publication	Population*	Group classification	Unit of measure of effect (outcome= OR and RR)	Measure of association of risk factors*	List of risk factors*	Routes of transmission
Alvarado-Esquivel, C et al [16].	Ann Hepatol	2020	Tepehuanos & Age- and gender-matched control subjects of the general population from rural settings.	Ethnic groups & Rural population	-	-	HEV seropositivity was associated with age, consumption of meat from goat, sheep, boar, turkey and pigeon, and concrete flooring at home.	-
Alvarado-Esquivel, C et al [15].	J Clin Med Res	2015	Mennonites & Age- and gender-matched non-Mennonites controls	Occupational group & Rural population	OR	HEV seropositivity was associated with increasing age (OR = 1.05; 95% CI: 1.00 - 1.09; P = 0.03).	-	-
Alvarado-Esquivel, C et al [12]..	Hepat Mon	2014	Adults in rural areas	Rural population	OR	HEV exposure was associated with: Increasing age (OR = 1.04; 95% CI: 1.04-1.05; P < 0.001) Consumption of untreated water (OR = 1.92; 95% CI: 1.06-3.46; P = 0.03) Availability of water at home (OR = 1.87; 95% CI: 1.07-3.27; P = 0.02).	Subjects with blood transfusion history had a higher (20/40: 50%) seroprevalence of HEV exposure than those without such clinical characteristic (80/233: 34.3%) (Borderline significance: P = 0.05). Subjects with history of transplantation had a similar seroprevalence (P = 0.62) of HEV infection than those without such clinical characteristic (2/4: 50% vs. 98/269: 36.4%, respectively).	
Alvarado-Esquivel, C et al [14]..	J Med Virol	2021	Waste pickers (cases) and controls	Occupational group & General population	OR	HEV seroprevalence increased with age OR 6.52, CI 1.95–21.78 and with Pig raising OR 12.01, CI 1.48–97.26	-	-
Alvarado-Esquivel, C et al [13].	Gastroenterol Res	2021	Butchers and people without an occupation of butcher	Occupational group & General population	OR	National trips was associated with HEV exposure (OR: 5.38; 95% CI: 1.02 - 28.16; P = 0.04)	HEV seroprevalence was higher in butchers with low education (up to 6 years) than those with higher education (more than 6 years) (P = 0.03)	
Alvarez-Munoz, MT et al [17].	Arch Med Res	1999	Young adults and children	General population	OR	Seroprevalence increased with age from 1.1% in children younger than 5 years to 14.2% in persons 26 to 29 years of age (OR 15.50	-	-

						(2.17–110.90), p = 0.006). Risk factors for infection included: Living in rural communities (OR 1.63 (1.16–2.30) p=0.005) and Low educational level (OR 2.08 (1.04–4.17) p=0.04).		
Andonov, A et al [18].	Vox Sang	2014	Patients received either solvent–detergent-treated plasma prepared by pooling of 2500 single-donor or cryosupernatant plasma.	Immunodeficiency	-	-	A distinct rise of anti-HEV IgG level was detected in two other TTP patients with weak pre-existing immunity to HEV; this observation is indicative of a possible immune response boost due to a breakthrough infection.	-
Atiq, M [22].	Emerg Infect Dis	2009	Persons with and without chronic liver disease	Immunodeficiency & General population	OR	The chance of being HEV positive increases as age increases (adjusted odds ratio [AOR] 1.05, p = 0.002, CI 1.02–1.09). Probability of HEV positivity is significantly greater if other liver disease is present (AOR 7.78, p<0.001, CI 3.43–17.64). Male gender was significantly associated with HEV positivity (AOR 2.07, p = 0.009, CI 1.17–3.66).	-	-
Bangueses, F et al [23].	J Med Virol	2020	Blood donors	Blood donors	-	-	A statistically significant difference in the anti-HEV Ig seroprevalence rate was observed between 18 to 30 and greater than 51 groups (P = .0065).	-
Baptista-González, H et al [24].	J Med Virol	2017	Pregnant women, Blood donors, acute viral patients, Healthy people	Pregnant women, Blood donors, & Viral hepatitis	-	-	The prevalence of HEV antibodies showed a higher prevalence in patients with acute viral hepatitis, with statistically significant difference (X2 8.2, P < 0.01).	-
Bartoloni, A et al [25].	Trop Med Int Health	1999	The study populations consisted of 295 individuals from Camiri and 292 individuals from Villa Montes.	Rural population	-	-	The prevalence of HEV antibodies in the population of the Camiri area (10.4%) was significantly higher than in the Villa Montes area (4.4%). In the population </= 30 years of age, the HEV seropositivity	-

							rate (4.4%) was significantly lower than in the >/= 31 year-old group.	
Bernal Reyes, R et al [26].	Rev Gastroenterol Mex	1996	General population	General population	OR	In the seropositives were predominance of males, older than 50 years, and low socioeconomic level (p = 0.013) (OR = 5).		-
Betancur, CA et al [27].	Acta méd. colomb	2013	Pig farmers	Pig related exposure	-	-	The contact with pigs was between one and 36 years with a mean of 7.87, in the positive cases was 10.36 years and in the negatives was 7 years. There was an association between more time with contact with pigs and IgG positive, but not statistically significant (p=0.171).	-
Bezerra, LA et al [28].	Acta Trop	2019	People living with HIV/AIDS (PLHA) receiving antiretroviral therapy (ART)	Immunodeficiency	OR	Piped water availability could be associated with the HEV infection in PLHA (OR: 0.08; CI 95%: 0.01-0.66; p = 0.0182).	-	-
Bricks, G et al [33].	Braz J Infect Dis	2018	Patients chronically infected with hepatitis C virus	Immunodeficiency	OR	Higher seroprevalence was found independently associated with: Age over 60 years (OR = 2.04; p = 0.02) and Previous contact with pigs (OR = 1.99; p = 0.03).	-	-
Bricks, G et al [32].	Braz J Infect Dis	2019	Patients chronically infected with HCV	Immunodeficiency	-	-	The independent variables age, HCV genotype, harmful alcohol use, and insulin resistance were found to be independently associated with hepatic cirrhosis.	-
Cabezas, C et al [34].	Plos One	2020	General Population	General Population	-	-	The prevalence of anti-HEV IgG was similar between men and women (P = 0.722) and increased significantly with age (P<0.0001).<br>There was a higher prevalence among Afro-Peruvians (23.5%), and significantly higher prevalence rates among people who had not completed primary education (22.5%; P<0.0001) as well as among housewives (16.2%; P = 0.007).	-

							Anti-HEV IgG prevalence was higher in the coastal region (14%, 95% CI 13.1–15.0), compared to the highlands and the jungle.	
Campbell, C [36].	University of Minnesota (Thesis)	2019	General population	General population	-	-	The IgG (lifetime seroprevalence) increases with age. Females had slightly higher seroprevalence than males. With respect to race, non-Hispanic Asians had significantly higher seroprevalence than any other ethnicity (12.8%) followed by non-Hispanic whites (6.8%) the other racial groups – non-Hispanic black, Mexican-American, other Hispanic, and other all had between 2% and 5% seroprevalence. Those born outside the U.S. had a higher seroprevalence than those born inside the U.S. (9.4% to 5.5%).	-
Campolmi, I et al [37].	Am J Trop Med Hyg	2018	Healthy volunteers from two rural communities	Rural population	OR	The seroprevalence of HEV increased with age (OR: 1.05 for each 1-year increase, 95% CI: 1.04–1.07, P < 0.001), exceeding 50% by the fifth decade of life	-	Fecal-oral transmission
Cangin, C et al [38].	J Med Virol	2019	General population	General population	OR	HEV (IgG/IgM) seropositivity was significantly associated with: "Non-Hispanic Asian" ethnicity (odds ratio [OR] = 1.69; CI = 1.12-2.56) Female (OR = 1.2, CI = 1.06-1.38) Increasing age (OR = 1.058, CI = 1.05-1.06).	Children and teenagers were less likely to be tested for HEV.	-
Cossaboom, CM et al [40].	J Med Virol	2016	Undergraduate and veterinary students at a major university	Occupational group	OR	The odds of subjects who answered that they “always” or “occasionally” consumed undercooked meat being positive for anti-HEV antibodies were 12.9 (95% CI, 1.707–97.19) times the odds of those who answered that they “never” consumed undercooked meat.	Consumption of undercooked meat was associated, age tended to be associated	-

Covarrubías, N et al [41].	Rev. chil. Infectol	2018	Blood donors	Blood donors	-	-	The seroprevalence increased with the age. The lowest rate was in people between 18 and 29 years (17,2%) in comparison with 30-39 years, 40-49 years and ≥ 50 years groups (21, 42,5 and 46,6%; respectively, p < 0,001)	-
Covarrubías, N et al [42].	Rev. chil. infectol.	2015	Pacients with previous analysis of anti-VHE IgG antibodies in the Laboratorio de Gastroenterología del Hospital Clínico de la Universidad de Chile	Viral hepatitis	-	-	A significant association between age and HEV IgG seropositivity was found (p < 0.001)	-
de Almeida, E. Araujo DC et al [44].	Int J Infect Dis	2020	Urban and rural residents (18-year or older) during visits to the local health center and routine home visits by family health professionals.	General population & Rural population	PR= prevalence ratio	Low education was significantly associated with HEV infection, and the prevalence ratio of subjects with low education compared to the other groups was 1.758 (95% CI, 1.015–3.044).	Gender, age, education level, household contact with hepatitis patients, and regular raw-meat consumption were included in the multivariate analysis model, and none of these variables was associated with the risk of HEV infection	-
de la Caridad Montalvo Villalba, M et al [45].	Infect Genet Evol	2013	Individuals who work in pig farms & workers without contact with pigs.	Pig related exposure & Occupational group	-	-	Serological anti-HEV prevalence showed a linear trend associated with age >= 41 years.<br>The prevalence of anti-HEV was higher in workers with an age range of 60-70 years old and timework 10-13years.	-
de la Caridad Montalvo Villalba, M et al [47].	Trans R Soc Trop Med Hyg	2010	Healthy individuals	General population	OR	Individuals included in this group had an OR 3.2 (1.09–9.7) times of being more likely to have been exposed to HEV than those included in the rest of the age groups; this finding was statistically significant (P= 0.01).	The prevalence of anti-HEV increased with age in both municipalities. Total anti-HEV was lower, 4.2% (5/117), in persons younger than 21 years; this variable increased to 13.1% (8/61) in age group 51–60 years old. The highest prevalence was found in persons aged 41–60 years (13.8%).	-
Debes, JD et al [51].	PLoS One	2016	HIV-infected individuals & Control group of HIV-negative individuals	Immunodeficiency & General population	OR	Based on logistic regression analysis the coefficient for CD4 count was significant, Z = -2.44, p = 0.01, with an odds ratio lower than 1: 0.967 (95% CI: 0.942–0.994) for a	-	-

						CD4 increase of 10 counts, indicating that individuals with low CD4 counts were more likely to be HEV seropositive. The higher risk for HEV seropositivity with decreasing CD4 count remained significant after adjusting for age (Z = -2.69, p = 0.006, OR = 0.969, 95% CI: 0.943–0.995).		
Dell'Amico, MC et al [52].	Emerg Infect Dis	2011	Persons in 2 rural communities (172 in Bartolo and 64 in Casas Viejas)	Rural population	-	-	The prevalence of IgG against HEV (7%) was higher in persons in Bartolo than in persons in Casas Viejas (4.7%) (p = 0.5). Seroprevalence did not show a linear trend associated with age; the highest seroprevalence was in found for persons 41–50 years of age in both communities (median age of 15 HEV seropositive persons 45 years, range 2–87 years). No sex-related differences in seroprevalence were observed in these communities (male participants 47%, female participants 53%).	-
Di Lello, FA et al [53].	Eur J Gastroenterol Hepatol	2020	Blood donors	Blood donors	-	-	A significant difference in blood donors' age was observed between anti-HEV IgG positive and negative individuals [44 (37-51) vs. 35 (27-43), P < 0.001, respectively].	-
Diehl, TM et al [54].	Gastroenterol Res Pract	2018	General population	General population	OR	Overall, 10.9% of participants who ingested self-grown foods had positive HEV antibodies versus 6.1% of participants who did not consume self-grown foods (P < 0.001; odds ratio (OR) 1.87; 95% CI 1.41-2.48). Approximately 9.7% (standard error of percent (SEP) 1.2) of participants who ingested self-grown foods had detectable HEV-specific IgG compared to	Approximately 7.3% (95% CI 6.2%–8.5%) of participants reported eating self-grown food, which represents an estimated 24,091,324 (95% CI 19,339,036–28,843,612) people nationally. Those who reported eating self-grown foods were older (median age 52.0; interquartile range (IQR) 36.5–66.0) than the remainder of the population (median 39.1; IQR 22.3–55.1; P < 0.001). In the age-stratified multivariable analysis, the	--

						5.4% (SEP 0.2) of participants who did not consume self-grown food (P < 0.001; OR 1.89; 95% CI 1.46–2.44).<br>HEV-specific IgM was detected in 1.7% (SEP 0.52) of those who reported consuming self-grown food, versus 1.0% (SEP 0.2) in those who did not (P = 0.21; OR 1.66; 95% CI 0.76–3.66).<br>When evaluating for any HEV antibodies (IgG or IgM), participants who reported eating self-grown foods had 10.9% seropositivity versus 6.1% in participants who did not consume self-grown foods (P < 0.001; OR 1.87; 95% CI 1.41–2.48).<br>In the age-stratified multivariable analysis, however, the correlation between ingesting self-grown foods and HEV seropositivity was significant for participants 40–59 years old (OR 1.70; 95% CI 1.06–2.74), but not overall, or for those < 40 years or ≥60 years.<br>Birth country was a significant associative factor, as the seroprevalence of participants born outside of the Unites States (9.3%; SEP 0.88; OR 1.78; 95% CI 1.23–2.58) was higher than that of US-born participants (6.0%; SEP 0.45).<br>In gender comparisons, young females (6–39 years) were found to have higher HEV seropositivity compared to males of the same age group (OR 1.44, 95% CI 1.03–2.02).<br>Non-Hispanic Black participants had lower rates	correlation between ingesting self-grown foods and HEV seropositivity was significant for participants 40-59 years old, but not overall, or for those < 40 years or ≥60 years. HEV-seropositive individuals were found to be older, with a median age of 57.6 years (IQR 45.0–69.0), compared to a median age of 40.7 years (IQR 23.7–56.3; P < 0.001) in the overall serum-tested population.	

						of HEV infection than White participants did (OR 0.60, 95% CI 0.46–0.79).		
Ditah, I et al [55].	Hepatology	2014	General population	General population	-	-	In the univariate analyses, factors associated with HEV seropositivity were increasing age (P-trend<0.001), birth outside of the U.S., Hispanic race, and "meat" consumption (>10 times/month). No significant association was observed with low socioeconomic status, water source, or level of education. In the multivariate analysis, only older age remained predictive of HEV seropositivity.	-
Eick, A et al [56].	J Infect Dis	2010	United States (US) servicemembers who were deployed to Afghanistan	Occupational group	OR	Age of more than 35 years was more frequent among seropositivesubjects (odds ratio, 2.9 [95% CI, 0.9–8.8]).	--	-
Fainboim, H et al [58]..	J Viral Hepat	1999	Blood donors & Anti-human immunodeficiency virus (HIV)-positive population	Immunodeficiency & Blood donors	-	-	Anti-HEV (6.6%) was significantly higher in anti HIV-positive patients than in control groups (1.8%), P = 0.0000	-
Fantilli, AC et al [59].	PLoS One	2019	Individuals with alcohol-related cirrhosis (140) vs. patients with alcohol use disorder (n=72, AUD, without cirrhosis) & controls (300)	Immunodeficiency, Exposed populations & General population	OR	The IgG anti-HEV seroprevalence in patients with cirrhosis was significantly higher than in healthy controls (25% vs. 4%, OR= 8,95% CI: 4–15.99, p<0.001). HEV seropositivity was significantly higher in alcoholic-related cirrhosis when compared to the general population (healthy control group)[OR= 15.7; (95% CI= 6.8–36.4); p<0.001] and to other causes of cirrhosis [39.5% vs. 12.4%; OR= 4.71; (95%CI= 1.9–11.6); p<0.05] Positive association between alcohol consumption and HEV seropositivity [12.5% vs. 4%; OR= 3.43; (95% CI= 1.39–8.49, p = 0.005)	-	-

						When comparing the HEV seroprevalence in individuals with alcoholic-related cirrhosis and AUD a statistically significant difference was found (39.5% vs. 12.5%; p = 0.0008), being more likely to be HEV seropositive in patients with alcoholic-related cirrhosis [OR= 4.58; (95% CI= 1.81–11.58)]		
Fearon, MA et al [60].	Transfusion	2017	Blood donors	Blood donors	OR	In case-control analysis: History of living outside Canada (odds ratio [OR], 2.9; 95% CI, 1.56-5.32), and Contact with farm animals (OR, 1.5; 95% CI, 1.01-2.28) were associated with HEV seropositivity.	Male sex (p 5 0.04) and increasing age (p < 0.0001) were associated with anti-HEV IgG antibody reactivity	-
Ferreira, AC et al [61].	Arch Virol	2018	HIV-infected patients	Immunodeficiency	-	-	No significant differences were observed when sex, age, CD4+ T cell count, HIV viral load, antiretroviral therapy, and ALT/AST levels were evaluated with respect to presence or absence of the HEV serological markers anti-HEV IgG and/or IgM	-
Focaccia, R et al [62].	Braz. j. infect. Dis	1998	Individuals of 2 years or older	General population	-	-	HEV showed zero prevalence among the age group between 2 and 9 years. This was followed by a slighty ascending rate starting from age 10, with an estimated prevalence of 1.05 percent (0.94 percent - 3.04 percent CI) among those 10 to 14 years of age. This infection reached its peak of 3.00% (0.55%-6.74% CI) at the age of 60 years or older.	-
Fontana, RJ et al [63].	Hepatology	2016	U.S. Acute Liver Failure Study Group	Immunodeficiency	-	-	Anti-HEV IgG+ subjects were significantly older, less likely to have APAP overdose, and had a lower overall 3-week survival compared to anti-HEV IgG- subjects (63% vs. 70%; P = 0.018).	-

Freitas, NR, et al [65].	Rev Soc Bras Med Trop	2017	Rural settlements in the southwest of the State of Goiás	Rural population	OR	Following multivariate analysis, the variable dwelling in a rural settlement for >5 years [odds ratio (OR), 3.4; 95% CI, 1.2-9.6] was associated with HEV seropositivity. Male sex (OR, 2.9; 95% CI, 0.9-9.3) was also associated with HEV seropositivity, with a borderline p-value.	-	
Freitas, NR et al [64].	Mem Inst Oswaldo Cruz	2016	Patients with acute non-A, non-B, non-C hepatitis	Viral hepatitis	-	-	In the univariate analysis, age over 35 years, low education level, habit of bathing in the river and living in rural area were associated with anti-HEV positivity. After multivariate analysis, low education level was independently associated with HEV seropositivity (p = 0.005), and living in rural area was marginally associated (p = 0.056).	-
Garcia, CG et al [68].	J Med Virol	2012	Patients with clinical features suggestive of acute viral hepatitis	Viral hepatitis	-	-	Most of the anti-HEV IgM positive cases were detected in patients younger than 20 years old (16/22 total cases, 73%). However, no difference was found in anti-HEV positivity between age groups. Among the 74 cases with acute hepatitis, the anti-HEV IgM positivity according to the gender was: 37% (10/27) of female and 26% (12/47) of males, being this difference not significant.	-
Goncales, NS et al [69].	Clin Diagn Lab Immunol	2000	Hospital employees, Volunteer blood donors (165 volunteer blood donors with alanine aminotransferase (ALT) levels ↕2 times the upper normal value (129 [78.2%] and 43 volunteer blood donors with ALT levels ¬2 times the	Occupational group, Blood donors & Exposed population	-	-	The prevalence of anti-HEV antibodies in health care professionals was not significantly different from that in healthy blood donors (3.0%, 5 of 165) and blood donors with raised alanine aminotransferase levels (7.5%, 3 of 40). The prevalence of anti-HEV antibodies (13.2%, 7 of 53) in cleaning service workers at a University hospital was similar to that	-

			upper normal value) & Women who attended a center for anonymous testing for human immunodeficiency virus (HIV) infection				among women at risk for HIV infection.	
Gutiérrez-Vergara, CC et al [70].	Iatreia	2015	People exposed to pigs, Cohabitant people of the exposed population & General population	Pig related exposure, Exposed population, & General population	PR	People occupationally exposed to pigs have more risk (PR: 2,42) to have anti HEV antobodies that those without exposition (CI 95%: 1,66-3,53) (p < 0,001)	-	-
Guzmán Rojas, P et al [153]	Diagnóstico (Perú)	2013	Slaughterhouses workers	Pig related exposure	OR	Those workers with a working time more than 20 years are more likely to have HEV antibodies (OR 8.81, 1.28-60.34)	-	-
Hardtke, S et al [73].	J Med Virol	2018	Pregnant women & female blood donors	Pregnant women & Blood donors	OR	Number (>3) of pregnancies (OR, 1.69; 95% CI, 1.04-2.75) were related to anti-HEV positivity	-	-
Hering, T et al [74].	J Med Virol	2014	Renal transplant patients	Immunodeficiency	-	-	Patients infected with HBV and/or HCV showed lower frequency of anti-HEV IgG (P¼0.009).	-
Ibarra, H et al [78].	Rev Med Chil	1997	Araucanian Indians, Blood donors, Health care workers & Inmates in state jails	Ethnic groups, Blood donors, Occupational group, & Exposed population	-	-	Prevalence of anti-HEV was not related to age and sex.	-
Koning, L et al [82].	BMC Infect Dis	2015	Patients who underwent liver transplantation for chronic Hepatitis C	Immunodeficiency	-	-	Seroprevalence of HEV at baseline was higher in men compared to women, however, overall seroprevalence from baseline up to last follow up did not differ between sexes: N = 60 (40 %) for men and N = 18 (33 %) for women (p = 0.418).	-
Kuniholm, MH et al [85].	J Infect Dis	2009	General population	General population	OR	Associated with significantly lower odds of HEV seropositivity:<br>Having a well as the source of tap water (OR, 0.78 [95% CI, 0.63–0.97]), and Having hepatitis A virus (HAV) seropositivity (OR, 0.80 [95% CI, 0.70–0.92]).	Among US-born individuals, males, non-Hispanic whites, and individuals residing in the Midwest and/or in metropolitan areas had the highest seroprevalence estimates.	-

						Associated with significantly higher odds of HEV seropositivity: Having a pet in the household (OR, 1.19 [95% CI, 1.01–1.40]), Having a dog in the household (OR, 1.22 [95% CI, 1.04–1.43]), Consuming liver or other organ meats more than once per month (OR, 1.38 [95% CI, 1.01–1.88]), and Having hepatitis C virus seropositivity (OR, 1.71 [95% CI, 1.07–2.74])		
Kyvernitakis, A et al [86].	Hepatol Res	2015	HCV-infected cancer patients	Immunodeficiency	OR	The factors independently associated with cirrhosis were: Male sex (odds ratio [OR], 2.8; 95% confidence interval [CI], 1.1–6.8; P = 0.028), and HEV seropositivity (OR, 4.1; 95% CI, 1.1–14.7; P = 0.032).	HEV seropositivity was associated with advanced age (P = 0.004), race (P = 0.02), place of birth outside the USA (P = 0.021), cirrhosis (P = 0.027), history of reused needles/syringes during massive vaccination campaigns (P = 0.015) and coronary artery disease (P = 0.039).	-
Lemos, G et al [88]	J Clin Virol	2000	Anti-HCV positive, Elevated ALAT, HBsAg positive, Healthy blood donors, Patients with sporadic acute viral hepatitis & Plasmapheresis	Viral hepatitis, Immunodeficiency, & Blood donors	-	-	The majority of HEV positives cases (16/24) were associated with the presence of fecal–oral HAV infection.	Fecal-oral transmission
Lopez-Santaella, T et al [91].	Ann Hepatol	2020	Pediatric patients	Children	-	-	Malnutrition has almost all cases PCR positive and the differences were significant only in comparison between malnutrition and overweight/obesity group (p < 0.005).	70% of blood transfusion patients were positive to HEV by PCR
Mahajan, R et al [93].	Emerg Infect Dis	2013	Persons who inject drugs	Exposed population	OR	Relative to participants <30 years of age, persons ≥30 years of age were more likely to be positive for IgG against HEV (odds ratio 3.61, 95% CI 1.31–9.94).	Prevalence was associated with age but not with homelessness, incarceration, or high-risk sexual behavior.	-
Martinez Wassaf, MG et al [94].	J Clin Virol	2014	Healthy adult population	General population	OR	Seroprevalence increased with age (OR: 3.50; 95% CI 1.39–8.87; p = 0.0065): the higher prevalence was found in older than 46 years old	-	-

						group (8.1%), while in young adults (<30 years old) prevalence was low (0.7%)		
Martinez, AP et al [95]	J Med Virol	2020	Young population under 18 years old, from rural areas of Chaco Province	Children	-	-	In whole population, 3 (1.4%) of the girls and 4 (2%) of the boys showed positive IgG anti-HEV antibodies serum, P = .674. When considering the at home availability of drinking water, six subjects (1.7%) lacked tap water, and only 1 (2%) had access to it, P = .877. Other analyzed conditions possibly related to the presence of HEV were crowding (Yes: 1.1%, No: 1.9%; P = .645), adobe house (Yes: 0%, No: 2.1%; P = .219), and presence of animals at home (Yes: 1.9%, No: 1.1%; P = .616).	-
Martins, RM et al [96].	J Clin Virol	2014	Population of recyclable waste pickers	Occupational group	OR	Age >40 years was independently associated with the presence of anti-HEV (OR 5.2, 95% CI 1.5-17.5).	-	-
Mast, EE [97].	J Infect Dis	1997	Blood donors	Blood donors	OR	Seroreactive persons were more likely than seronegative persons to have traveled to countries in which HEV is endemic (odds ratio [OR] for MPr-EIA = 4.3, P < .001; OR for RPr-EIA = 2.5, P = .005), but 31% of MPr-EIA anti-HEV-reactive persons and 38% of RPr-EIA anti-HEV-reactive persons had no history of international travel.	-	-
McGivern, DR et al [98].	Open Forum Infect Dis	2019	Persons with chronic HBV in the Hepatitis B Research Network Cohort Study	Immunodeficiency	OR	The odds of anti-HEV seropositivity (IgG+ or IgM+) were higher in: Older participants (1.05 (1.03–1.07)), Males (2.10 (1.39–3.19)), Asians and less educated people (2.92 (1.42–6.01)), and those born outside the United States and Canada (2.09 (1.03–4.25)).	-	-

Meng, XJ et al [99].	J Clin Microbiol	2002	Blood donors, Swine veterinarians from other countries, Swine veterinarians from the eight U.S. states & Veterinarians working with swine	Pig related exposure & Blood donors	OR	Swine veterinarians in these eight states with blood donor controls were 1.51 times more likely to be anti-HEV positive than were normal blood donors when tested with swine HEV antigen (95% confidence interval, 1.03 to 2.20) and 1.46 times more likely to be anti-HEV positive when tested with Sar-55 antigen (95% confidence interval, 0.99 to 2.17).	Anti-HEV prevalence in swine veterinarians and normal blood donors was age specific and paralleled increasing age. There was an association between age and prevalence of anti-HEV both in swine veterinarians and in blood donors	-
Miernyk, KM et al [100].	Vector Borne Zoonotic Dis	2019	Avian wildlife biologists, Persons with no wild bird exposure, Sport bird hunters, Subsistence bird hunters & Subsistence family members	Occupational group, General population & Ethnic groups	-	-	Compared to non-Native persons, Alaska Native persons they were significantly less likely to be seropositive to HEV (4.1% vs. 0.4%; > = 0.01). Seropositivity to HEV was associated with increasing age ($p < 0.01$)	-
Minuk, GY et al [101].	Can J Gastroenterol	2007	Indigenous North American population (Canadian Inuit community)	Ethnic groups	-	-	Seven of 11 (64%) were also positive for anti-hepatitis A virus, five (46%) were hepatitis B virus seropositive and none (0%) were positive for anti-hepatitis C virus. There were no associations between infections with HEV and other hepatropic viruses.	-
Moraes dos Santos, DC et al [102].	Mem Inst Oswaldo Cruz	2002	Residents from Manguinhos community assisted at the Health Unit Sinval Germano Faria/ National School of Public Health (ENSP-Fiocruz)	General population	-	-	The highest prevalence rate was observed in the group ranging from 41-50 years old and over 60 years ($P = 0.002$). The male group had a higher anti-HEV IgG prevalence rate (5.7%) than the female group (1.3%) ($P = 0.002$)	-
Munne, MS et al [103].	J Clin Virol	2011	A. Adults with acute non A-C hepatitis. B. Children with acute non A-C hepatitis. C. Children with fulminant liver failure (FHF) and hepatitis A.	Viral hepatitis	-	-	Trip to India or Cuba or Santo Domingo or Miami, work with children in low sanitary condition, work in a health center, low sanitary condition, fishing and wash hands and stuff in river, contact with sick people	-

Ooi, WW et al [105].	Am J Trop Med Hyg	1999	Travelers abroad	Exposed population	-	-	Seroconverters were statistically different (P , 0.01) from nonconverters with respect to a greater number of developing countries previously visited; more frequent consumption of uncooked foods while traveling; more frequent close contact with natives, including shared meals; and more exposures to human secretions (saliva, stool, and blood).	-
Pang, L et al [108].	Am J Trop Med Hyg	1995	Gold miners	Occupational group	-	-	The mining camps have poor sanitation with a great potential for fecal-oral transmission of disease.	Fecal-oral transmission
Passos-Castilho, AM et al [115].	Braz J Infect Dis	2017	Blood donors	Blood donors	OR	A 4-fold increase of the risk to undergo a HEV infection or present with positive anti-HEV IgG antibodies at an advanced age (45–59) than at an early age (OR 3.96 (1.54–10.22)). Anti-HEV IgG prevalence varied significantly among the demographic zones in the city of Sao Paulo. Highest rates were observed in the Central (OR 6.93 (1.15–41.61)) and South Central (6.28 (1.22–32.22)) zones.	-	-
Passos-Castilho, AM et al [114].	Journal of Medical Virology	2016	Blood Donors	Blood Donors	OR	A threefold increase of the risk of being infected with HEV at an advanced age (>50) than at an early age (OR=3.33, (1.11–9.95)).	Categorized age groups revealed an age-dependent increase of HEV seroprevalence.	-
Passos-Castilho, AM et al [112]	Rev Soc Bras Med Trop	2015	Patients clinically suspected of being HEV carriers at clinics or hospitals	Viral hepatitis	-	-	It was significantly associated with age group, with the highest frequencies in persons older than 40 years (median age of 47 HEV seropositive patients: 46 years, range: 10-94 years; median age of seronegative patients: 36 years, range: 0-91 years; $p < 0.0001$). The highest IgM frequencies were observed in people aged 10-19 years (12.5%; 7/56) and 0-9 years (5.3%; 2/38) (median	-

							age of 27 HEV IgM-positive patients: 36 years, range: 1-59 years; median age of IgM-negative patients: 36 years, range: 0-94 year	
Pelaez, D et al [116].	Biomedica	2016	Serum from patients with positive diagnosis for viral hepatitis	Viral hepatitis	-	-	According to the age range, 48% of the coinfection with HAV and HEV was in the group younger than 16 years old and, 9%, in the group of 16 to 30 years old. The majority of those infections was detected in regions with poor sanitation practices and drinkable water. 28% of the cases of coinfection between HBV and HEV was in the group of 2 to 15 years old, 32%, in the 16 to 30 years, and 8% in the one of 46 to 70 years.	-
Perez, OM et al [118].	Am J Trop Med Hyg	1996	Healthy population	General population	OR	The anti-HEV prevalence was significantly higher in individuals having their water supply outside rather than inside their home (OR 3. 15, 95% CI =1.38-7.17, P <0.01).	HEV seropositivity was observed in adults, the seroprevalence in > 40-year-old individuals being 17.6%, respectively.	-
Pisano, MB et al [120].	Arch Virol	2017	Patients on dialysis & Solid organ transplant recipients	Immunodeficiency	OR	In univariate and multivariate analyses, consumption of fish was associated with higher seroprevalence of HEV (OR = 9.33; 95% CI: 2.07-42.2; p = 0.04).	Significantly higher seroprevalence of HEV IgG were found in those undergoing dialysis compared with healthy controls (10.2% and 4.3% respectively, p = 0.03). No difference in HEV seroprevalence was observed between healthy controls and transplant recipients (5.8%). No association between previously identified risk factors for HEV was found, such as pork consumption or use of tacrolimus, and HEV seroprevalence.	-
Pisano, MB et al [119].	Trans R Soc Trop Med Hyg	2018	People who attended health care centres near the Arias–Arenales River for routine control.	General population	-	-	A trend of increasing prevalence with age was observed (25% in adults >46 y of age, 7.4% in adults 31–45 y of age and 2.4% in young adults [<30 y of age] and children).	-

Pujol, FH et al [121].	J Med Virol	1994	Rural Amerindians (Padamo, Edo Amazonas), Rural populations (San Camilo, Edo Apure) & Urban pregnant woman (Caracas)	Ethnic groups, Rural population & Pregnant women	-	-	The prevalence of antibodies in the Amerindian group was significantly higher than in urban pregnant women. No relation was found between age and HEV prevalence in rural populations.	-
Quintana, A et al [123].	J Med Virol	2005	General population	General population	-	-	Associations between the presence of anti-HEV antibodies and some risk factors for HEV infection (sex, race, age, acupuncture, tattoo, history of animals in the house, history of surgery, transfusion, and history of hepatitis) were not observed ($P<0.05$).	-
Remondegui, C et al [6].	Rev Argent Microbiol	2021	Guarani ethnic group	Ethnic groups	-	-	In the three age groups (0-20, 21-40 and older than 41) the seroprevalences were 5.3%, 14.7% and 11.7%, respectively.	-
Rendon, J et al [126]	PLoS One	2016	Patients aged over 15 with a clinical diagnosis of viral hepatitis	Viral hepatitis	-	-	HEV infection was more frequent in patients aged 34–40 (66%, 6/9) and in male patients (66%, 6/9). The variables local water supply, garbage collection system not available, pigs in the house and type of household flooring were not significantly associated with HEV cases	-
Rey, JA et al [127].	J Travel Med	1997	Blood donors, Children & Patients before they were subjected to invasive procedures	Blood donors, Children, & Immunodeficiency	-	-	The difference in prevalence of anti-HEV between GA and GB was highly significant (p=.0080), as well as the difference between GA and GB versus the -prevalence in GC (p=.0000).The mean age between GA and GB was significantly higher than the mean age in GC (p=.0000).There were no significant differences in distribution according to sex.	-
Rodriguez Lay, L et al [128].	J Med Virol	2008	Outbreaks cases of acute viral hepatitis & Sporadic clinical cases	Viral hepatitis	-	-	People under 15 years old were also susceptible to HEV infection. In co-infected HAV/HEV people there were positive results in all age groups, ranging from 25.8% in individuals under 15 years of	-

							age to 15.4% in individuals of 15 years of age and older.	
Sherman, KE et al [130].	AIDS Res Hum Retroviruses	2021	HCV Monoinfected and HCV/HIV coinfected	Immunodeficiency	-	-	Age was a significant factor, with older (>60 years) subjects having higher prevalence than those younger (p=0.0097).	-
Sherman, KE et al [131].	J Viral Hepat	2014	HIV-infected kidney transplant candidates & HIV-infected liver transplant candidates	Immunodeficiency	-	-	Kidney transplant candidates with a positive IgG were significantly older than those without an IgG response (median age 51 vs. 45 years; p=0.049). Among kidney cases, the proportion of subjects with baseline anti-HEV IgG response was significantly higher in HCV-infected group (5/12 (42%)) compared to HCV non-infected group (5/41 (12%)) (Fisher's exact; p=0.04). Overall levels of IgG and IgM were higher among liver transplant candidates than kidney transplant candidates.	-
Silva, SM et al [132].	Mem Inst Oswaldo Cruz	2012	Blood donors & Individuals exposed to swine in the rural areas	Pig related exposure & Blood donors	-	-	When comparing the anti-HEV-positive participants who had been exposed to swine with the anti-HEV-negative participants, the authors noticed associations between the presence of anti-HEV and increased age, a history of blood transfusions and contact with other farm animals. However, after a multivariate analysis was performed, this association was not confirmed.	-
Smith, HM et al [134].	J Infect Dis	2002	Patients who used a free clinic in downtown (Most of these patients had been homeless during the previous year)	Exposed population	-	-	Patients who had antibody to HEV were significantly older (mean age, 48 years; p=.0005) than those who did not (mean age, 40 years). This association did not reach significance for the other agents (p>10).	-
Stramer, SL et al [137].	Transfusion	2016	Blood donors	Blood donors	OR	Donors in the Midwest (12.5%; 95% CI, 10.8%-14.4%) versus all other regions combined (5.3%; 95% CI, 4.5%-6.2%) were more likely to be antibody	-	-

						positive (OR, 2.23; 95% CI, 1.92-2.88; p<0.0001). Similar results occurred when the Midwest was compared to each of the remaining five regions individually except for the Northeast (Midwest vs. the Northeast: OR, 1.22; 95% CI, 0.89-1.67; p50.2161).		
Sue, PK et al [138].	Open Forum Infect Dis	2016	Solid Organ Transplant Recipients (Children and adults)	Immunodeficiency	OR	Multivariate conditional logistic regression also demonstrated an association between risk of post-transplant HEV infection and graft rejection (OR, 14.2; P = .03; 95% CI, 1.26–160)	-	-
Talarmin, A et al [139].	J Med Virol	1997	Banks of sera (collected for epidemiological studies on human T-cell leukemia/ lymphoma virus type I, during routine testing of pregnant women, or for the identification of arboviruses)	Ethnic groups	OR	The seroprevalence rates differed significantly between ethnic groups (trend test, P < 0.001), with higher rates among Brazilians (OR, 4.1; CI, 1.8–9.4), Chinese and Hmongs (OR, 4.4; CI, 1.8–10.7), and Haitians (OR, 3.1; CI, 1.1– 8.7). These differences remained after adjustment for age.	Seropositivity to HEV did not increase with age, but differences were observed between places of residence (trend test, P < 0.05).	-
Tejada-Strop, A et al [140].	Am J Trop Med Hyg	2019	Pregnant Women	Pregnant women	-	-	The prevalence of IgG anti-HEV in the non-West region (12.3%) was significantly greater than that in the West region (5.3%) (P < 0.0001). The non-West region had a higher prevalence of HEV. HEV infection prevalence increased with increasing age. Compared with pregnant women aged 15–19 years, women aged ≥ 35 years had a higher prevalence of anti-HEV IgG (16% versus 6%).	-

Teshale, EH et al [141].	J Infect Dis	2015	Noninstitutionalized civilian population	General population	-	-	For the 1988–1994 participants, risk factors associated with higher antibody prevalence in bivariate ($\chi 2$) analyses were military service, never having used cocaine or crack, less frequent consumption of processed meats, and hepatitis A virus (HAV) antibody positivity. After adjustment for age, sex, race/ethnicity, and region of residence, only anti-HAV positivity remained significantly associated with anti-HEV positivity. For the 2009–2010 participants, higher antibody prevalence was significantly associated in bivariate analyses with having a well, cistern, or spring as the source of tap water for drinking; with less frequent consumption of processed meats; and with more-frequent consumption of green leafy vegetables or lettuce salad. Not drinking tap water was significantly associated with a lower antibody prevalence. After adjustment for age, sex, race/ethnicity, and region of residence, the only risk factor significantly associated with seropositivity was more-frequent consumption of processed meats, but this factor was protective.	-
Thomas, DL et al [142].	J Clin Microbiol	1997	Blood donors, Homosexual males & Injection drug users (IDUs)	Blood donors, & Exposed population	OR	More than 45 years: OR 3.2 (1.1, 10.7).	Among homosexual men, anti-HEV was not significantly correlated with a history of hepatitis, high-risk sexual practices, or sexually transmitted infections, in contrast to hepatitis A and B antibodies. Among IDUs, anti-HEV was not significantly associated with a history of hepatitis or	-

							high-risk drug-using practices, as was found with hepatitis C antibodies.	
Tissera, G et al [143].	BMC Infect Dis	2020	Pregnant women & non-pregnant women	General population & Pregnant women	OR	Association between pregnancy and HEV infection (p = 0.023, OR = 3.5, CI95% = 1.1-10.5). Statistical association between low age of pregnancy (≤25 years old) and HEV seroprevalence (OR = 3.2, CI95%: 1.2–8.9).	When dividing the samples into group ages, the ≤25 years old group showed the higher seropositivity value (16.7%, p = 0.032). No significant difference was found in the HEV seroprevalence when analyzing the samples according to the studied years.	-
Unzueta, A et al [145].	Ann Hepatol	2016	Patients evaluated for heart and kidney transplantation	Immunodeficiency	OR	On multivariable analysis, age older than 60 years was associated with HEV infection (adjusted odds ratio, 3.34; 95% CI, 1.54-7.24; P = 0.002).	-	-
Vildosola, H et al [146].	Rev. gastroenterol. Perú	2000	Workers in water and sewage supply companies	Occupational group	-	-	Working time in the company was between 13 to 20 years.	-
Vitral, CL et al [147].	BMC Infect Dis	2014	Agricultural settlements in the Amazon Basin	Ethnic groups	OR	Increasing age was significant determinant of HEV seropositivity (OR, 1.033; 95% CI, 1.016-1.050; P < 0.001).	The overall HEV positivity rate was 12.9% (95% CI, 9.5-16.2%), with the highest seroprevalence (19.4%) in young adults (21–30 years of age).	-
Withers, MR et al [149].	Am J Trop Med Hyg	2002	Swine workers & Non-swine worker (Control)	Pig related exposure & General population	-	-	Non-U.S.-born workers were more likely to be seropositive (9 of 32 [28.1%]) than were U.S.-born workers (9 of 133 [6.8%]; P 0.001). Country of birth was a statistically significant risk factor (P 0.003).	-
Xu, C et al [150].	Transfusion	2013	Blood donors	Blood donors	-	-	A significant (p < 0.001) stepwise increase in anti-HEV seroprevalence was seen with increasing age.	-
Zafrullah, M et al [151].	Transfusion	2018	Blood donors	Blood donors	-	-	Anti-HEV IgG/total anti-HEV detection rates increased with age. Interassay agreement was higher among the IgG anti-HEV/total anti-HEV assays (84%) than the IgM assays (22%). Regression analyses of signal-to-cutoff ratios from IgG/total antibody assay were heteroskedastic, indicating no constant variance among these	-

							assays, suggesting they may detect different epitopes or were affected by waning or less avid antibodies in the US donor population.	
Zhang, L et al [152].	Hepatology	2015	Racial/ethnic populations from the Third National Health and Nutrition Examination Survey	Ethnic groups	-	-	Seropositivity was associated with age, rising significantly with increasing age across all three subpopulations and peaking in the >65-year age group. Seropositivity was significantly associated with region of residence, poverty income ratio, education attained, and country of birth	-

*Direct reference from the studies

6. Genotypes found in the literature

Table 6. Reported HEV Genotypes

Country	First author	Journal	Year of publication	Region	Start of study	End of study	Genotype & Sup
Argentina	Pisano, MB et al [119].	Trans R Soc Trop Med Hyg	2018	South America	2015	2016	3
Argentina	Martinez Wassaf, MG et al [94].	J Clin Virol	2014	South America	2009	2010	3
Argentina	Munne, MS et al [104].	Ann Hepatol	2014	South America	2011	2013	3a & 3i
Argentina	Munne, MS et al [103].	J Clin Virol	2011	South America	2005	2010	1a, 3a, 3b, &3i
Bolivia	Dell'Amico, MC et al [52].	Emerg Infect Dis	2011	South America	2006	2006	3
Brazil	Passos, AM et al [111].	J Med Virol	2013	South America	1998	2007	3
Brazil	Lopes Dos Santos, DR et al [90].	J Clin Virol	2010	South America	2004	2008	3b
Canada	Halac, U et al [72].	Gut	2012	North America	1992	2010	3
Canada	Andonov, A et al [18].	Vox Sang	2014	North America	2001	2003	3a
Colombia	Rendon, J et al [126].	PLoS One	2016	South America	2008	2009	3
Colombia	Pelaez, D et al [116].	Biomedica	2015	South America	2004	2014	3a
Cuba	de la Caridad Montalvo Villalba, M et al [48].	Emerg Infect Dis	2008	Caribbean	1999	2005	1
Cuba	de la Caridad Montalvo Villalba, M et al [46].	Rev Biomed	2016	Caribbean	2013	2013	1d
Cuba	de la Caridad Montalvo Villalba, M et al [45].	Infect Genet Evol	2013	Caribbean	2007	2007	3a
Mexico	Lopez-Santaella, T et al [91].	Ann Hepatol	2020	North America	2012	2014	3f
Mexico	Realpe-Quintero, M et al [124].	Intervirology	2018	North America	2015	2016	1
United States	Engle, RE et al [57].	J Clin Microbiol	2002	North America	-	-	3 & 1
United States	Kuniholm, M. H et al [84].	Hepatology	2016	North America	1984	2013	3a
Uruguay	Bangueses, F et al [23].	J Med Virol	2020	South America	2017	2018	3
Venezuela	Garcia, CG et al [68].	J Med Virol	2012	South America	2008	2008	3 & 1

7. Publication bias

Fig 1 shows funnel plots for IgG seroprevalences included in the meta-analysis and related to blood donors, general population, viral hepatitis, and immunodeficiency subgroups. In all cases, the plots reveal a considerable heterogeneity in the estimated seroprevalences.

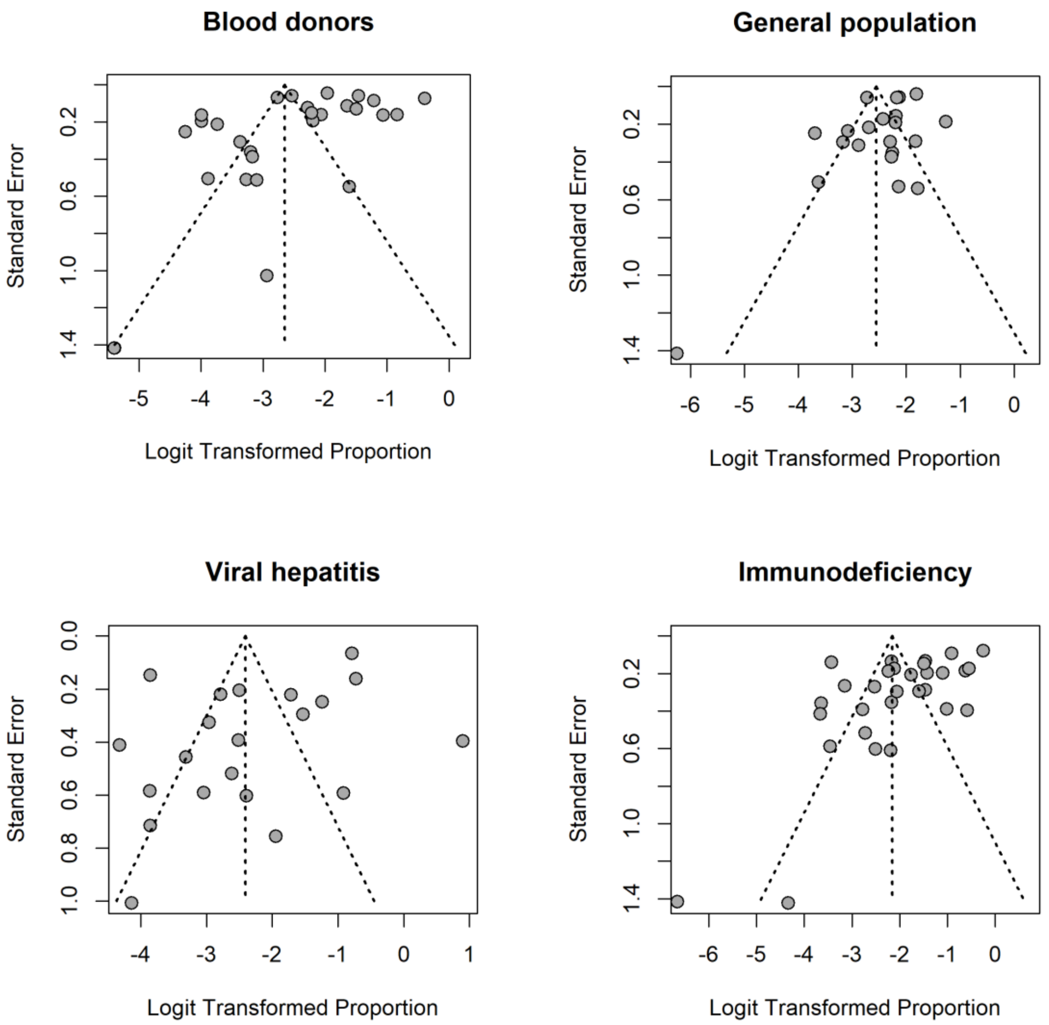

Fig 1. Funnel plots of anti-HEV IgG studies related to blood donors, general population, patients with viral hepatitis and with immunodeficiency included in the meta-analysis. The region in which 95% of studies are expected to lie is constructed based on the random-effects GLMM.

Fig 2 shows the number of available results per country and population subgroup reporting on IgG. It shows that we have abundant evidence for Argentina, Mexico, Brazil and the USA. For other countries, the number of available studies is low, and in some cases only studies focused on special subpopulations are available, which can distort the general picture of HEV seroprevalence in the respective country (for example only studies in people with pig exposure and viral hepatitis in Colombia).

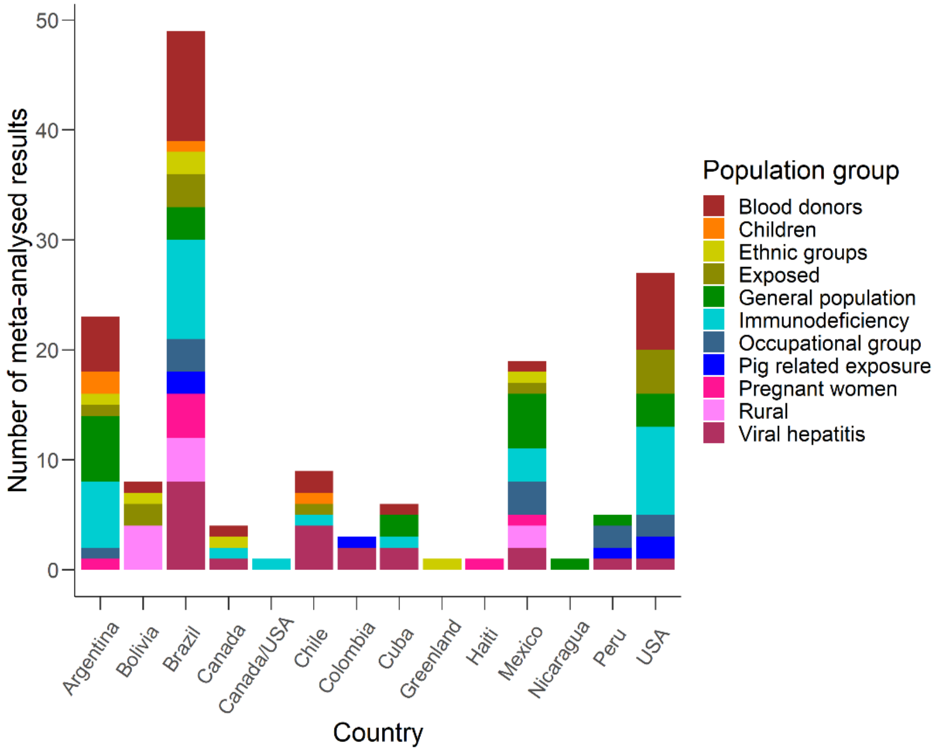

Fig 2. Number of available results per country and considered population subgroups reporting on IgG seroprevalence.

8. Overview of results obtained by different tests

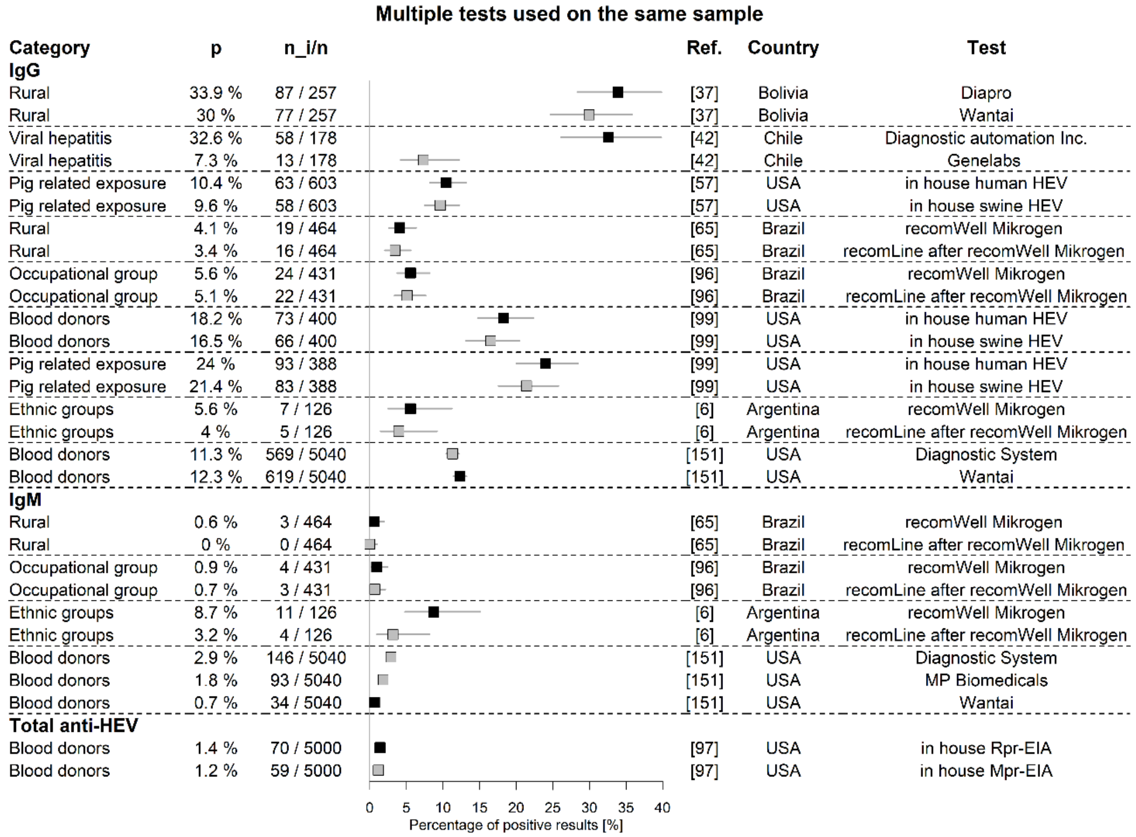

Fig 3. Overview of positivity obtained by analyzing a given sample using different tests. The test strategy "recomLine after recomWell Mikrogen" refers to a procedure when a positive sample by recomWell had to be confirmed by recomLine to count as positive. Black color denotes results included in our main analyses. A sensitivity analysis (replacing the chosen values by their alternatives one at a time, and all at once) was carried out to quantify the impact of the choice on our results. Note that except for two cases (IgG in viral hepatitis [42] and IgM in blood donors [151]) the different tests yielded very similar results.

9. Percentages of Total anti-HEV positive results

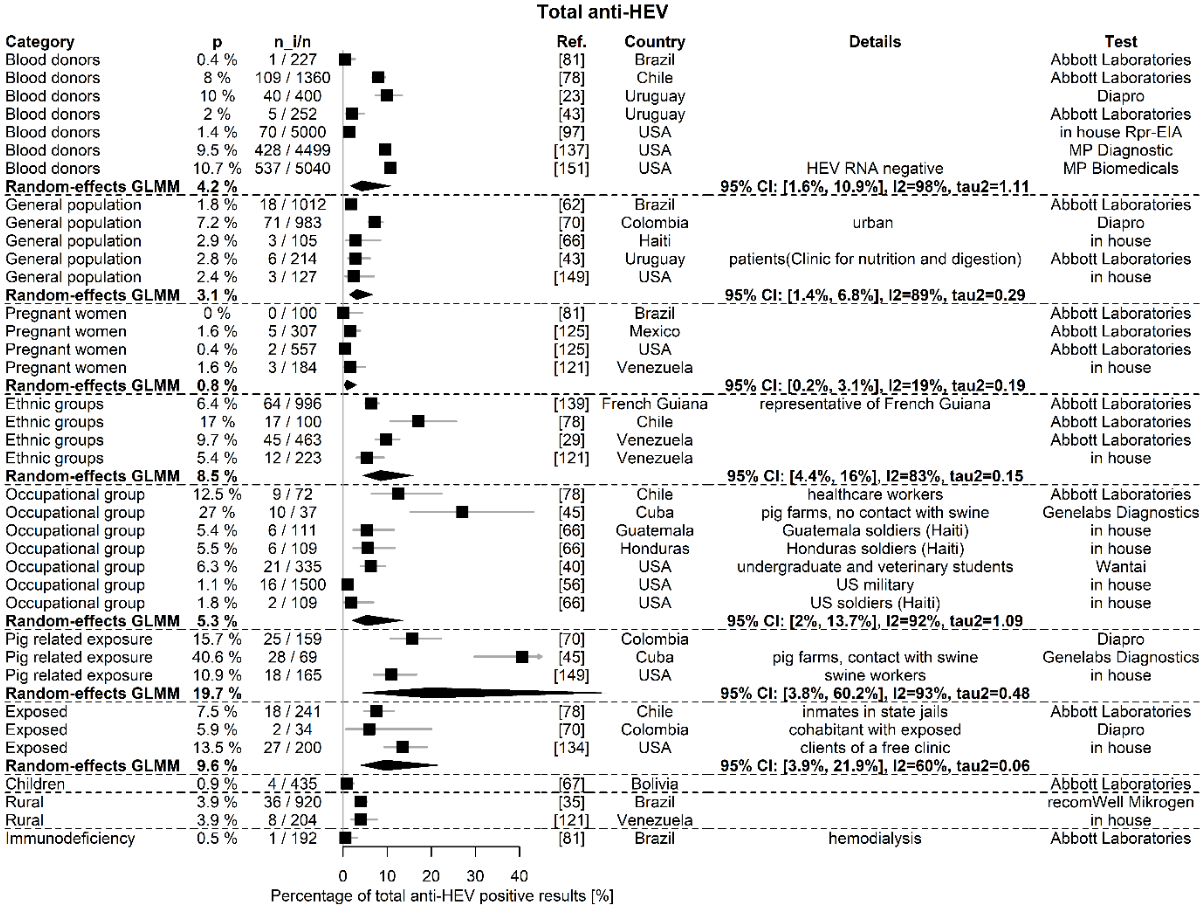

Fig 4. Percentages of total anti-HEV positive samples with 95% Agresti-Coull confidence intervals (truncated to the permissible range 0-100%) and the pooled estimates in the subgroups obtained from the random-effects generalized linear mixed model. The 95% confidence intervals for the pooled seroprevalences were calculated using a t-quantile. In a sensitivity analysis, we checked that using the alternative value for the result for blood donors in [98] (see also **Fig 3**) in the pooling does not have any considerable effect on the results (pooled estimate for blood donors 4.1%, 95% CI: 1.5%-10.9%, I^2=98%, tau^2=1.19).

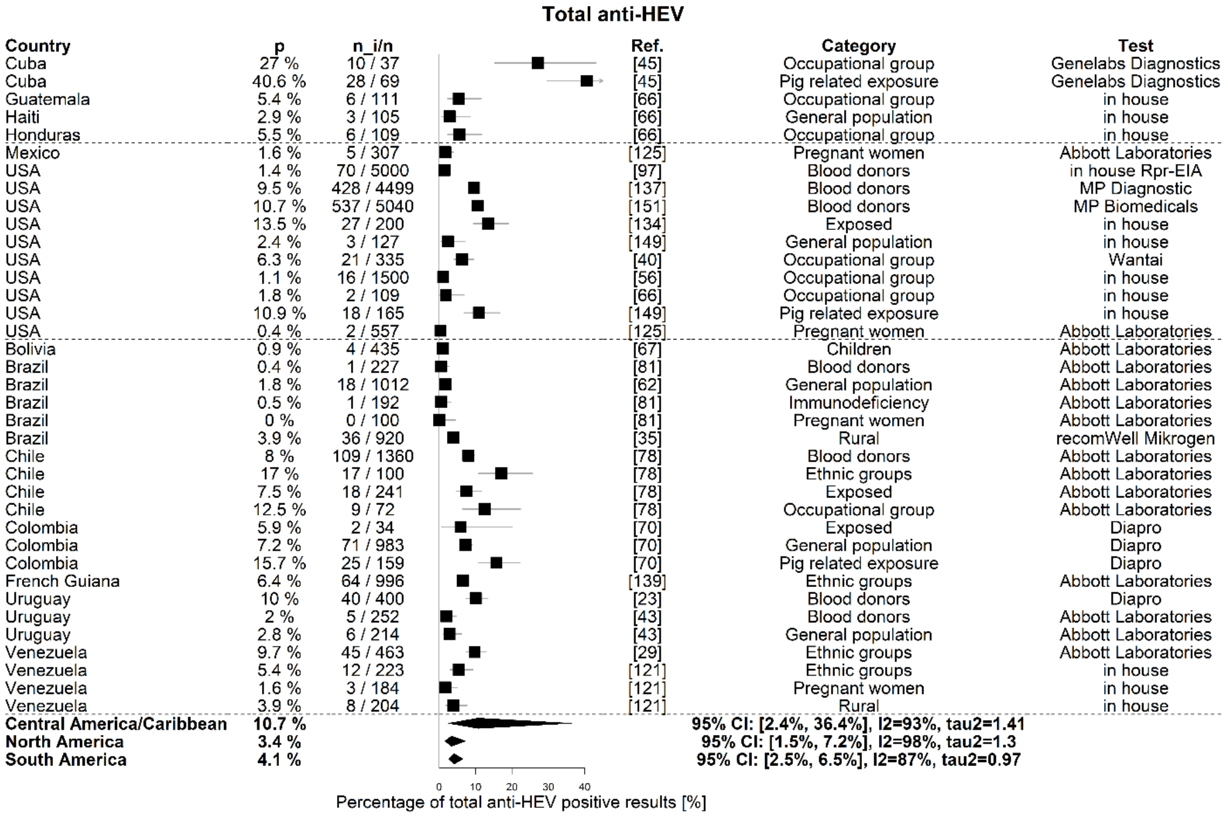

Fig 5. Percentages of total anti-HEV positive samples with 95% Agresti-Coull confidence intervals (truncated to 0-100%) sorted by country and region and the pooled estimates in the regions obtained from the random-effects generalized linear mixed model. The 95% confidence intervals for the pooled seroprevalences were calculated using a t-quantile. Since individual studies contribute several results into the pooling and these may be correlated, we also considered a model with an additional random effect for results stated in one paper; however, with no practical changes to the pooled estimates: South America: 3.8%, 95% CI: 2.1%-7.1%; North America: 3.5%, 95% CI: 1.5%-7.8%; Central America and the Caribbean: 13.8%, 95% CI: 1.4% – 64.1%.

10. Overview of NHANES results

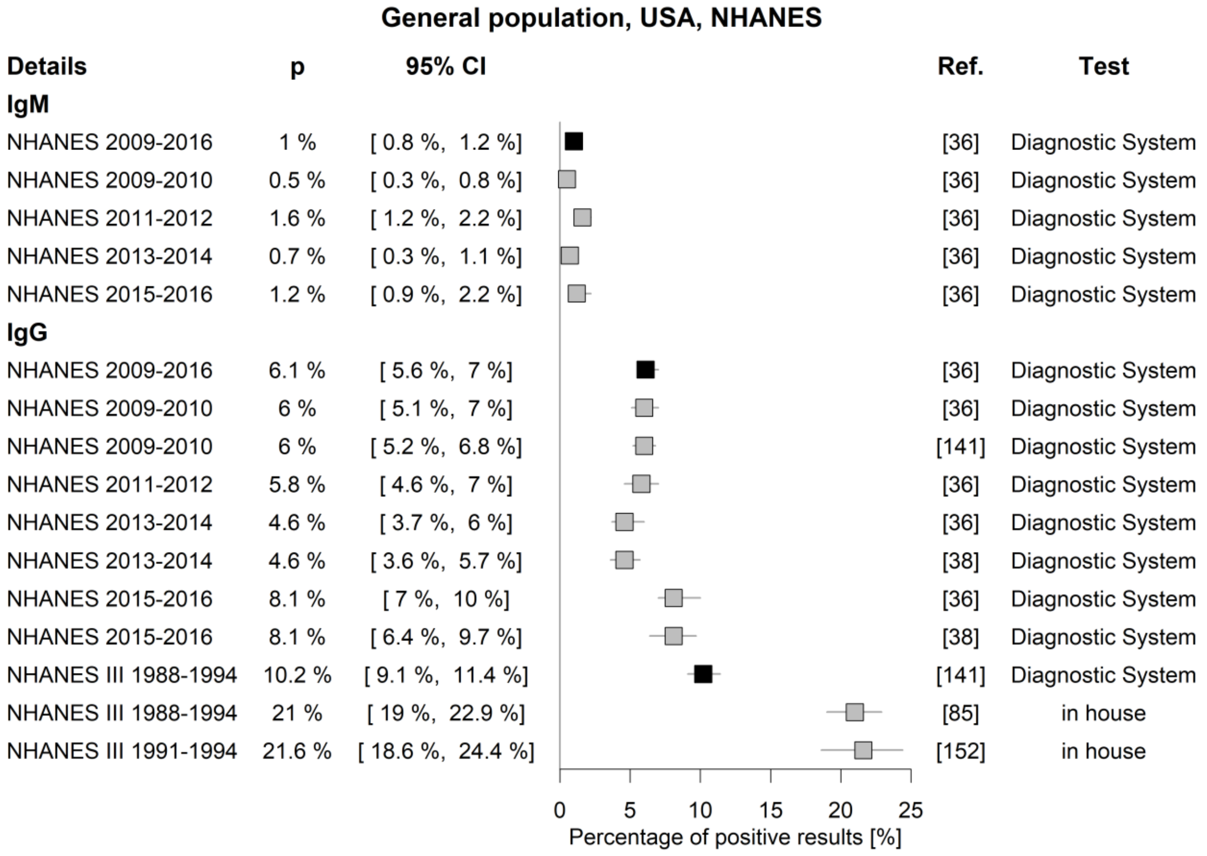

Fig 6. Seroprevalences and their 95% confidence intervals as reported by studies analyzing NHANES data. Black color denotes results included in our main analyses. They estimate the seroprevalence over a larger time interval and are based on the same test. Results in gray show the fluctuation of seroprevalence estimates when considered over shorter periods and/or when obtained by an (previously applied) in-house test.

11. Percentages of IgG positive results

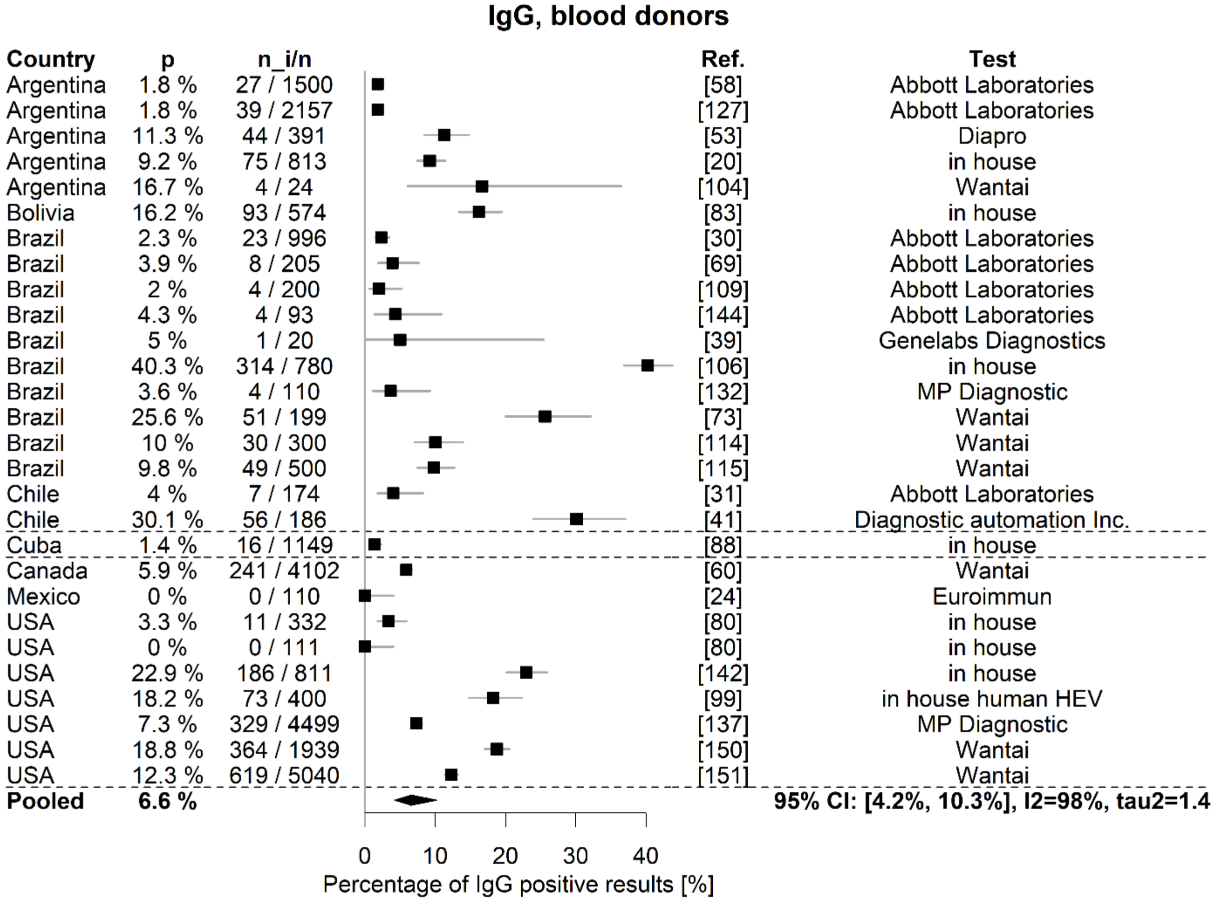

Fig 7. Seroprevalences reported for blood donors ordered according to the region: South America, Central America and the Caribbean, and North America. Shown are 95% Agresti-Coull confidence intervals (truncated to 0-100%) and the pooled estimate from the random-effects GLMM with its 95% confidence interval obtained using t-quantiles. Pooling within each region (with at least three seroprevalences) separately yielded: 7.3%, 95% CI: 4.3% - 12.3%, tau^2=1.20, I^2=98% (South America), and 6.1%, 95% CI: 2.0% - 17.1%, tau^2=1.90, I^2=98% (North America), without a significant difference between the regions (Q test, p=0.7317). In a sensitivity analysis we checked that using the alternative values for [99] and [151] from **Fig 3**, does not have a practical influence on the results.

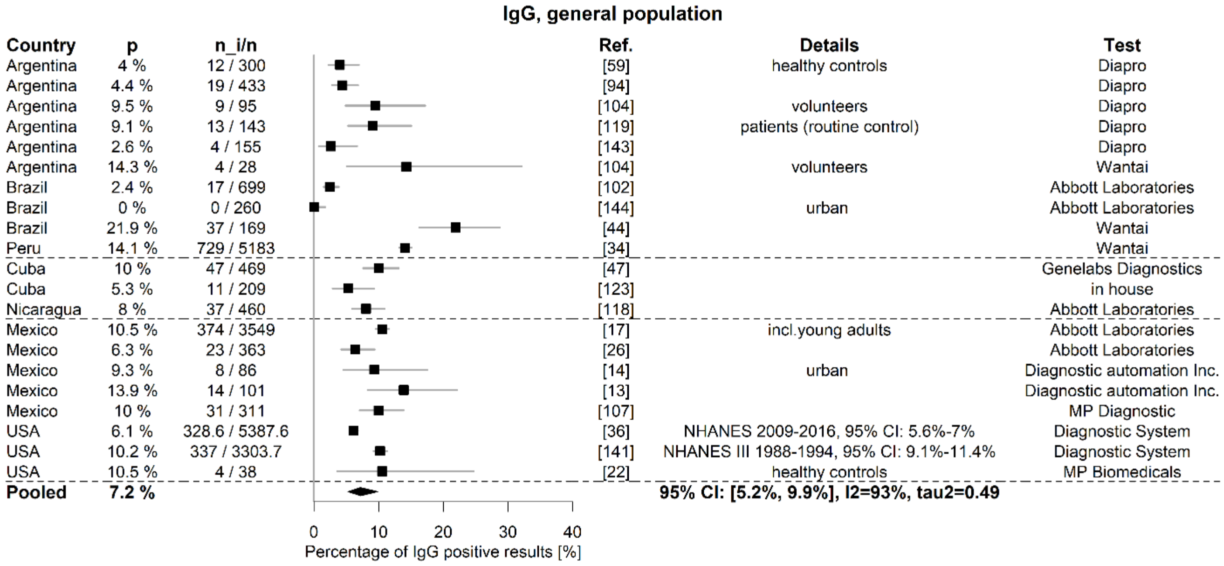

Fig 8. Seroprevalences reported for general (adult) population ordered according to the region: South America, Central America and the Caribbean, and North America. Shown are 95% Agresti-Coull confidence intervals (truncated to 0-100%) and the pooled estimate from the random-effects GLMM with its 95% confidence interval obtained using t-quantiles. Pooling within each region separately yielded: 5.4%, 95% CI: 2.4% - 11.8%, tau^2=1.24, I^2=93% (South America), 8.2%, 95% CI: 4.7% - 14.0%, tau^2=0.01, I^2=53% (Central America and the Caribbean), and 9.0%, 95% CI: 7.0% - 11.3%, tau^2=0.06, I^2=91% (North America), without a significant difference between the regions (Q test, p=0.3730). Please, note that the shown sample sizes for NHANES studies are only proxies as described in the Methods section.

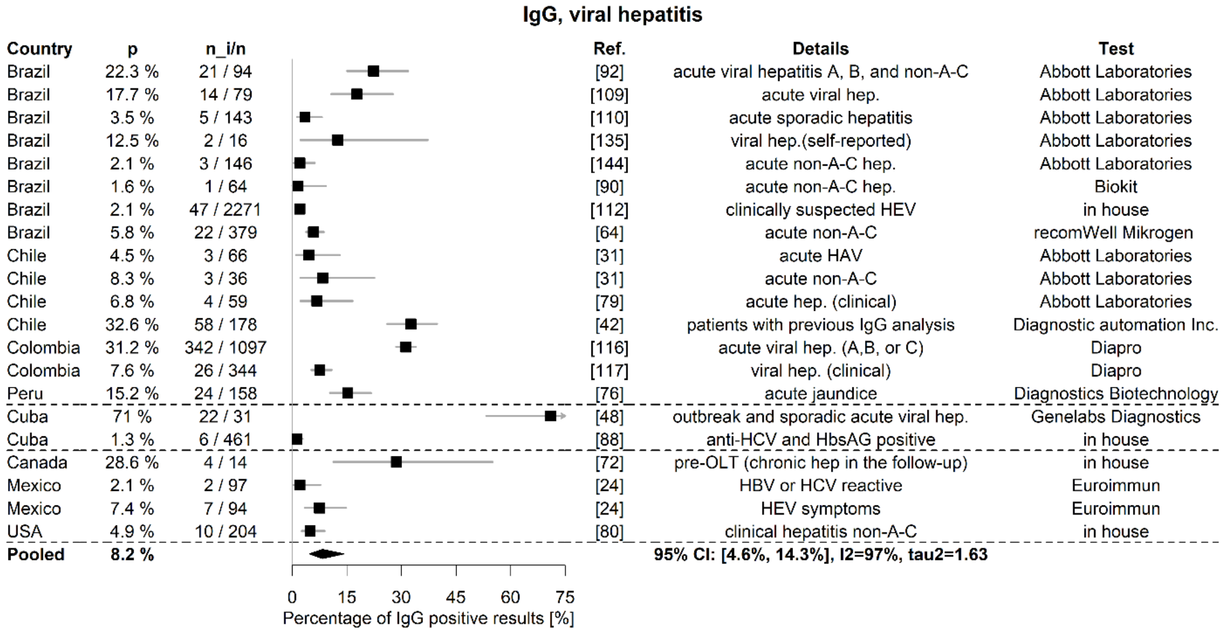

Fig 9. Seroprevalences reported for viral hepatitis ordered according to the region: South America, Central America and the Caribbean, and North America. Shown are 95% Agresti-Coull confidence intervals (truncated to 0-100%) and the pooled estimate from the random-effects GLMM with its 95% confidence interval obtained using t-quantiles. If the large seroprevalence reported from Cuba using outbreak samples is left out, the pooled result remains practically the same: 7.1%, 95% CI: [4.3% - 11.7%, I^2=97%, tau^2=1.14. Pooling within each region (with at least three reported seroprevalences) separately yielded: 8.2%, 95% CI: 4.6% - 14.2%, tau^2=1.05, I^2=97% (South America), and 6.5%, 95% CI: 1.6% - 23.0%, tau^2=0.58, I^2=76% (North America), without a significant difference between the regions (Q test, p=0.6320). In a sensitivity analysis, we checked that using the alternative value for [42] from **Fig 3** does not have a practical effect on the results.

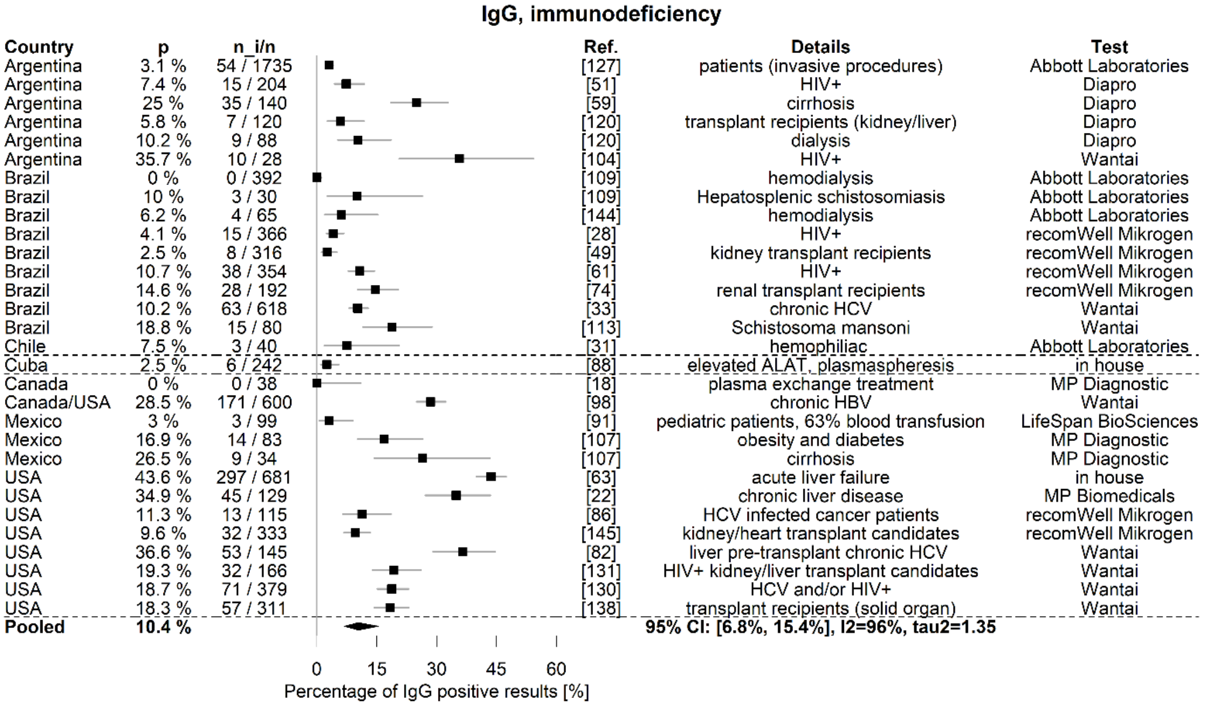

Fig 10. Seroprevalences reported for the immunodeficiency subgroup ordered according to the region: South America, Central America and the Caribbean, and North America. Shown are 95% Agresti-Coull confidence intervals (truncated to 0-100%) and the pooled estimate from the random-effects GLMM with its 95% confidence interval obtained using t-quantiles. Pooling within each region (with at least three reported seroprevalences) separately yielded: 7.4%, 95% CI: 4.2% - 12.8%, tau^2=1.15, I^2=91% (South America), and 17.7%, 95% CI: 10.9% - 27.5%, tau^2=0.77, I^2=94% (North America), with a significant difference between the regions (Q test, p=0.0099).

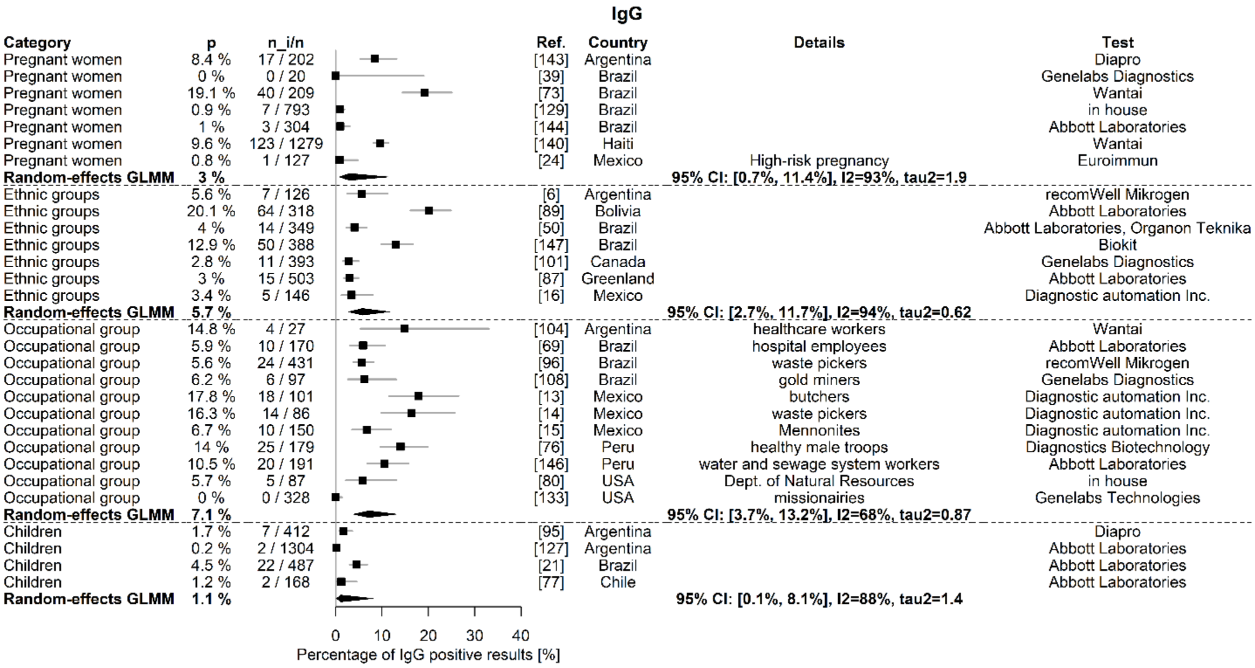

Fig 11. Seroprevalences reported for pregnant women, ethnic groups, occupational groups and children. Shown are 95% Agresti-Coull confidence intervals (truncated to 0-100%) and the pooled estimate from the random-effects GLMM with its 95% confidence interval obtained using t-quantiles. In a sensitivity analysis we checked that using the alternative values for [6] and [96] from **Fig 3** does not have a practical effect on the pooled results.

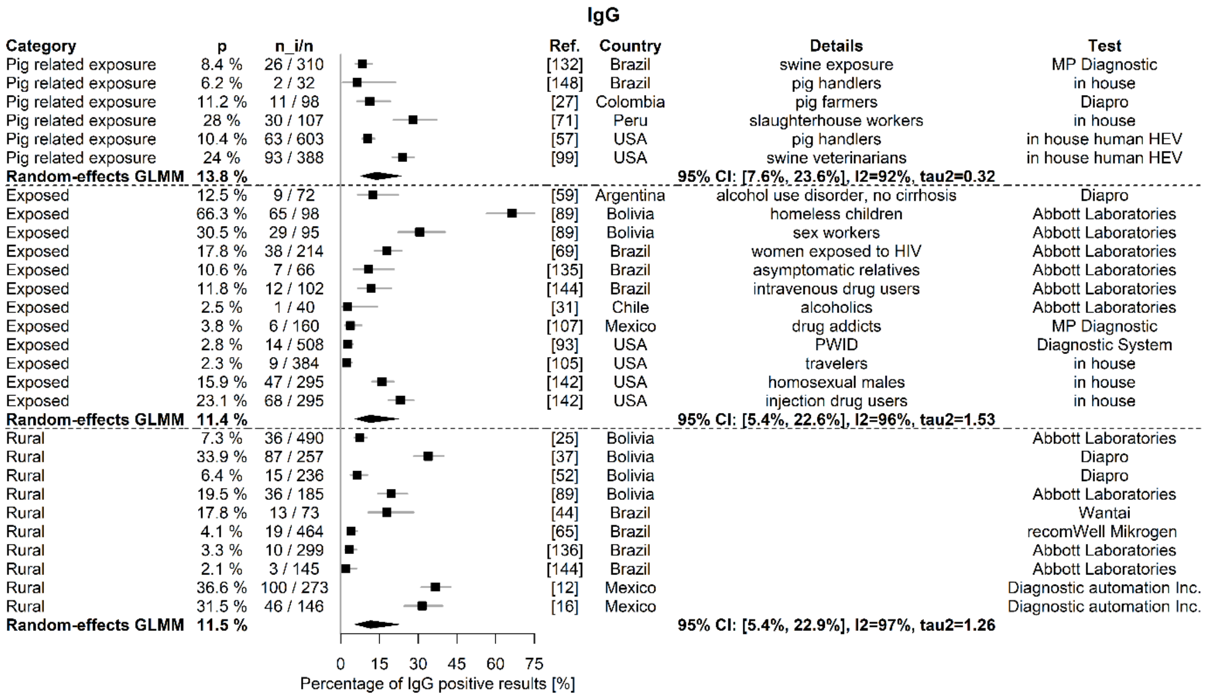

Fig 12. Seroprevalences reported for pig related exposure, exposed and rural populations. Shown are 95% Agresti-Coull confidence intervals (truncated to 0-100%) and the pooled estimate from the random-effects GLMM with its 95% confidence interval obtained using t-quantiles. In a sensitivity analysis, we checked that using the alternative values for [37, 57, 99, and 65] from **Fig 3** does not have a practical effect on the results.

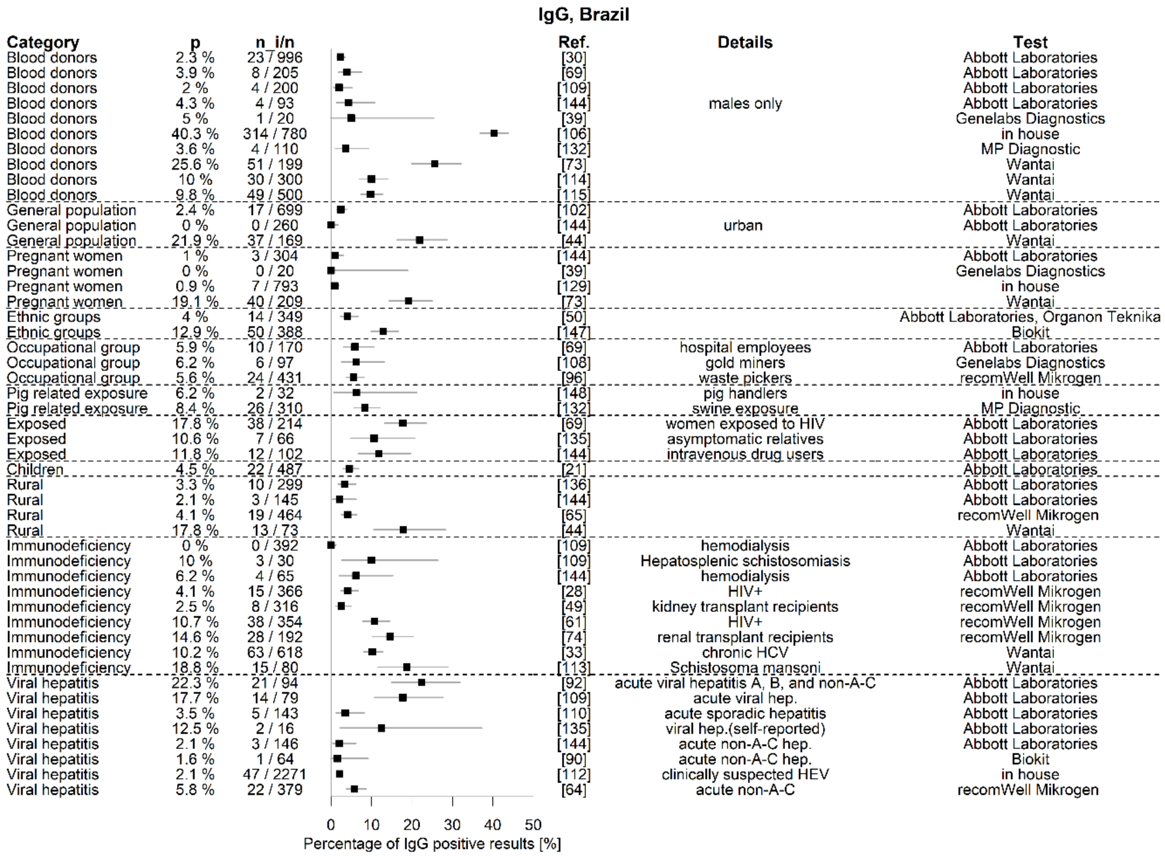

Fig 13. Seroprevalences reported in samples from Brazil together with 95% Agresti-Coull confidence intervals. The plot illustrates the considerable amount of variability in results reported from a single country.

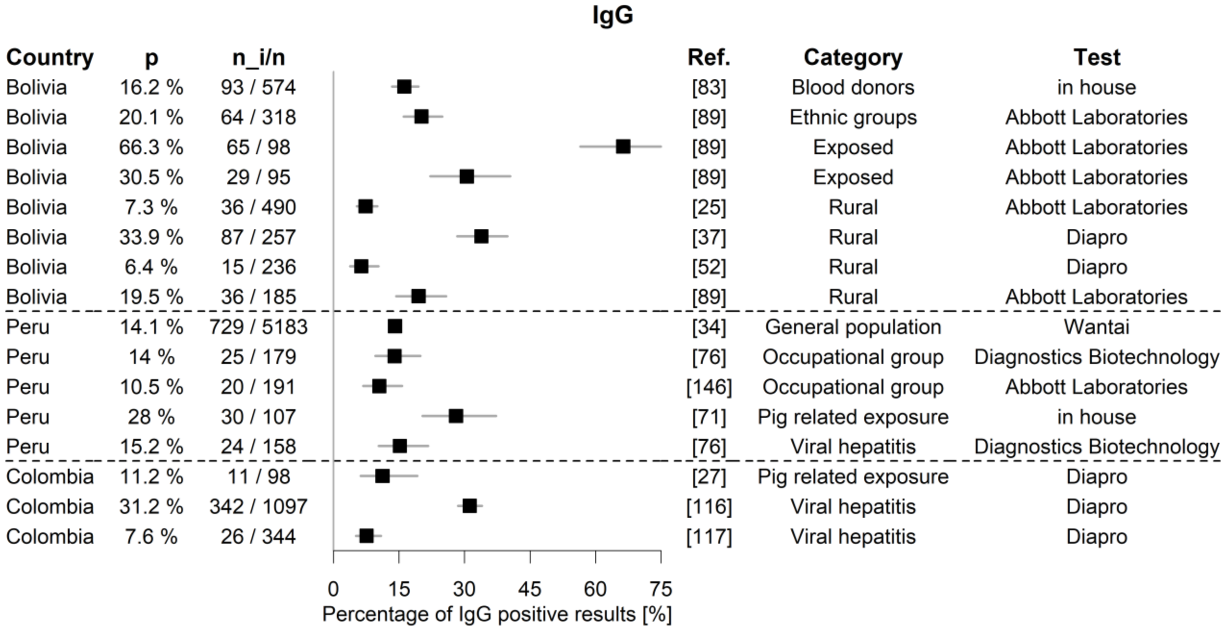

Fig 14. Seroprevalences reported from Peru, Bolivia and Colombia with 95% Agresti-Coull confidence intervals. These three countries tended to report higher seroprevalences than the rest. It is, however, to note that the overall number of the reported seroprevalences is rather low and not all subgroups are represented in the published studies. This may account for the apparent difference.

12. Percentages of IgM positive results in total sample

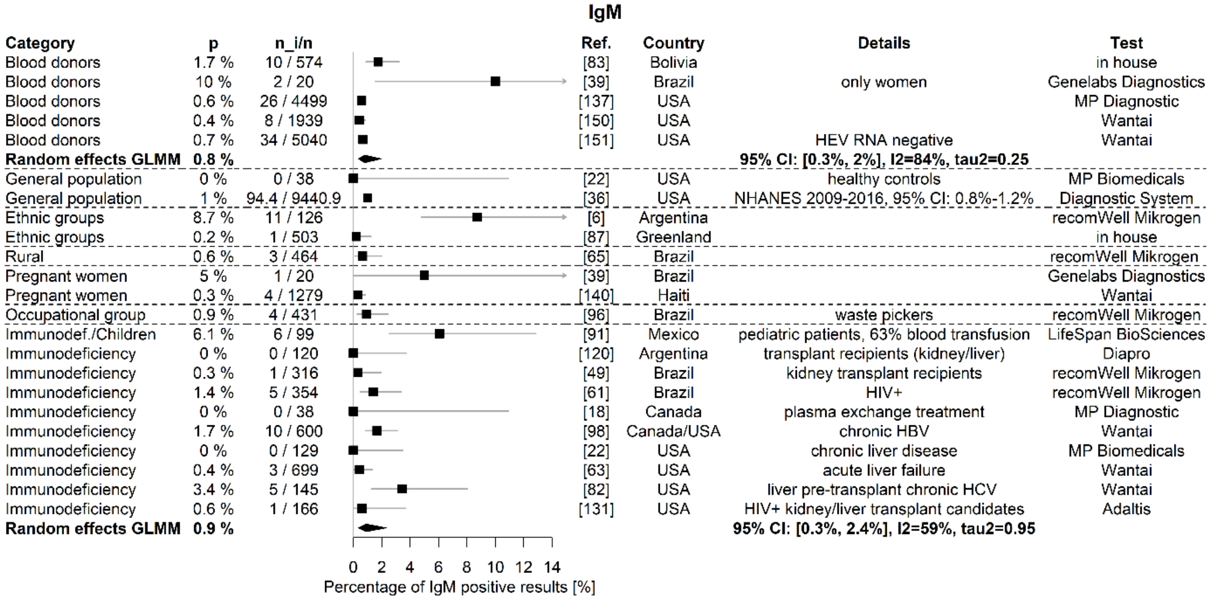

Fig 15. Observed IgM-seroprevalence without viral hepatitis patients. Depicted are 95% confidence intervals constructed by the Agresti-Coull method (truncated to 0-100%), and the pooled seroprevalence (in subgroups with at least three observations) coming from a random effects GLMM model. In a sensitivity analysis, we checked that the alternative values for [151] from **Fig 3** do not have any practical effect on our results (they resulted in an increased I^2 above 90%, larger tau^2, wider CIs and slightly higher pooled estimates: 1.2% and 1.3%). Please, note that the shown sample size and number of positive samples for NHANES study are only proxies as described in the Methods section.

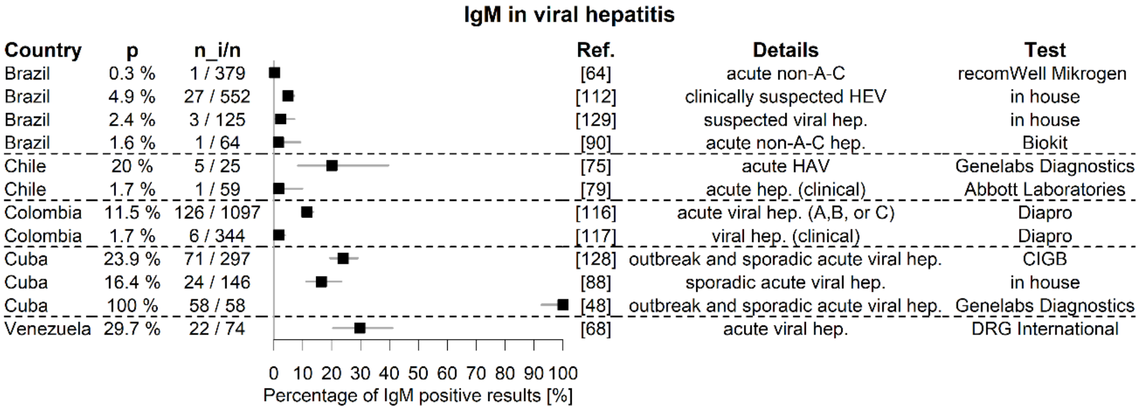

Fig 16. Observed IgM-seroprevalence in viral hepatitis patients. Depicted are 95% confidence intervals constructed by the Agresti-Coull method (truncated to 0-100%). Without the apparent HEV outbreak with a seroprevalence of 100%, pooling by random-effects GLMM would yield a seroprevalence of 5.5%, 95% CI: 2.0% - 14.1%, I^2=93%, tau^2=2.10. This pooled value is significantly (Q-test, p=0.0006) different from the pooled estimate in all other subgroups considered together (**Fig 15**).

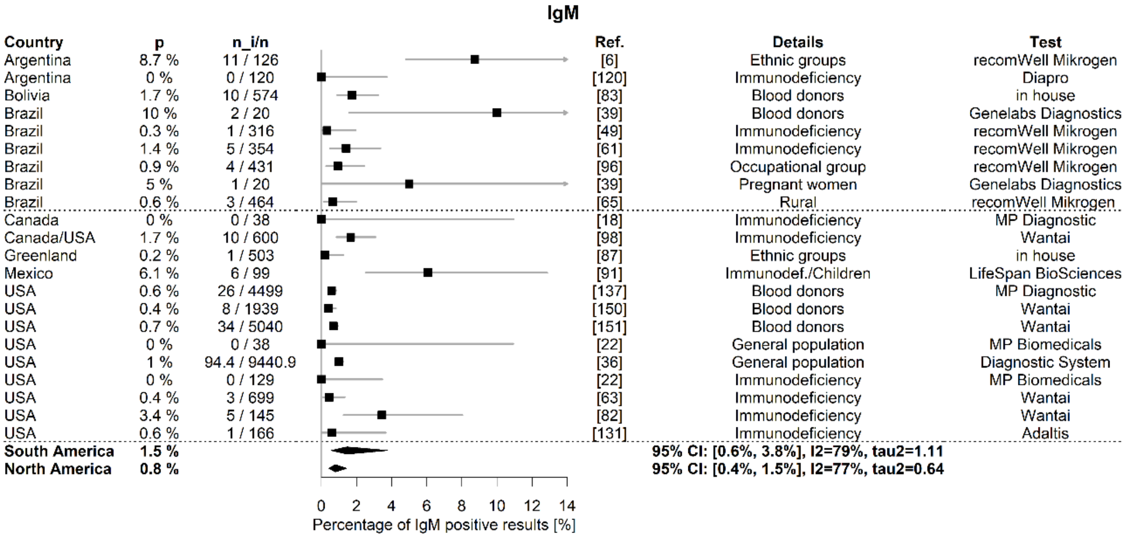

Fig 17. IgM-seroprevalence (without viral hepatitis) in South and North America with 95% Agresti-Coull confidence intervals (truncated to 0-100%) and the pooled seroprevalences in both regions coming from a random effects GLMM. The pooled results were not found to be significantly different (Q-test, p=0.2211). Please, note that the shown sample size and number of positive samples for NHANES study [36] are only proxies as described in the Methods section.

13. Percentages of IgM positive results among IgG or total anti-HEV positive samples

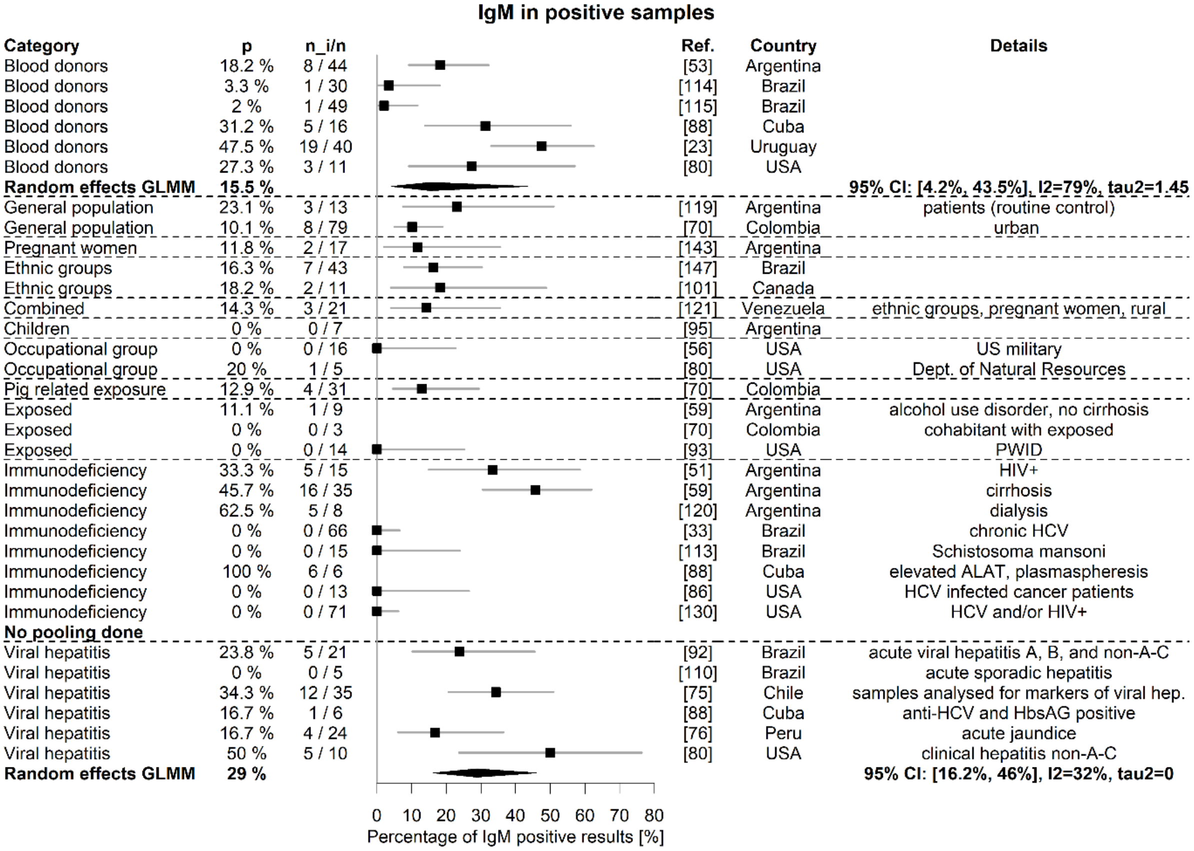

Fig 18. Percentages of IgM positive results in IgG or total anti HEV positive samples. Depicted are the 95% Agresti-Coull confidence intervals for proportions (truncated to 0-100%) and pooled seroprevalences with 95% confidence intervals (based on t-quantiles) in selected subsets of the results. The immunodeficiency subgroup with values either equal to zero or larger than 30% was considered too heterogeneous for the pooling. Note that results based on sample sizes less than 10 are only listed, but not graphically depicted, or meta-analyzed.

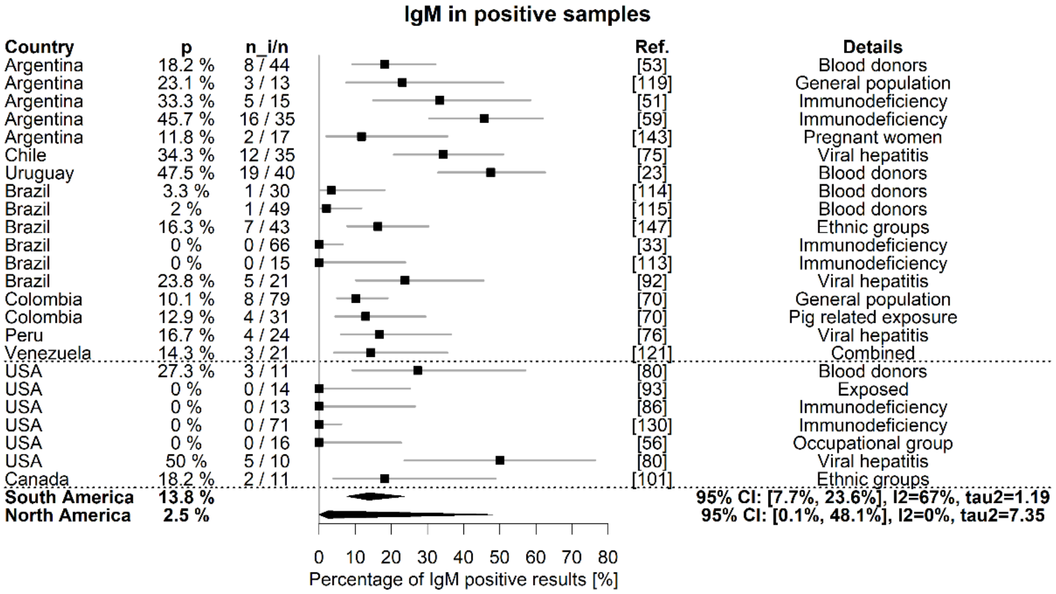

Fig 19. Pooled percentages of IgM positive results in IgG or total anti HEV positive samples for countries in South and North America together with the 95% confidence intervals (using t-quantiles). The apparent differences in the pooled estimates were not statistically significant at 5% level (Q-test, p=0.2180). The single results are depicted with the 95% Agresti-Coull confidence intervals for proportions (truncated to 0-100%). Note that we selected all reported results based on a sample size of at least 10.

14. Percentages of RNA positive results in total sample

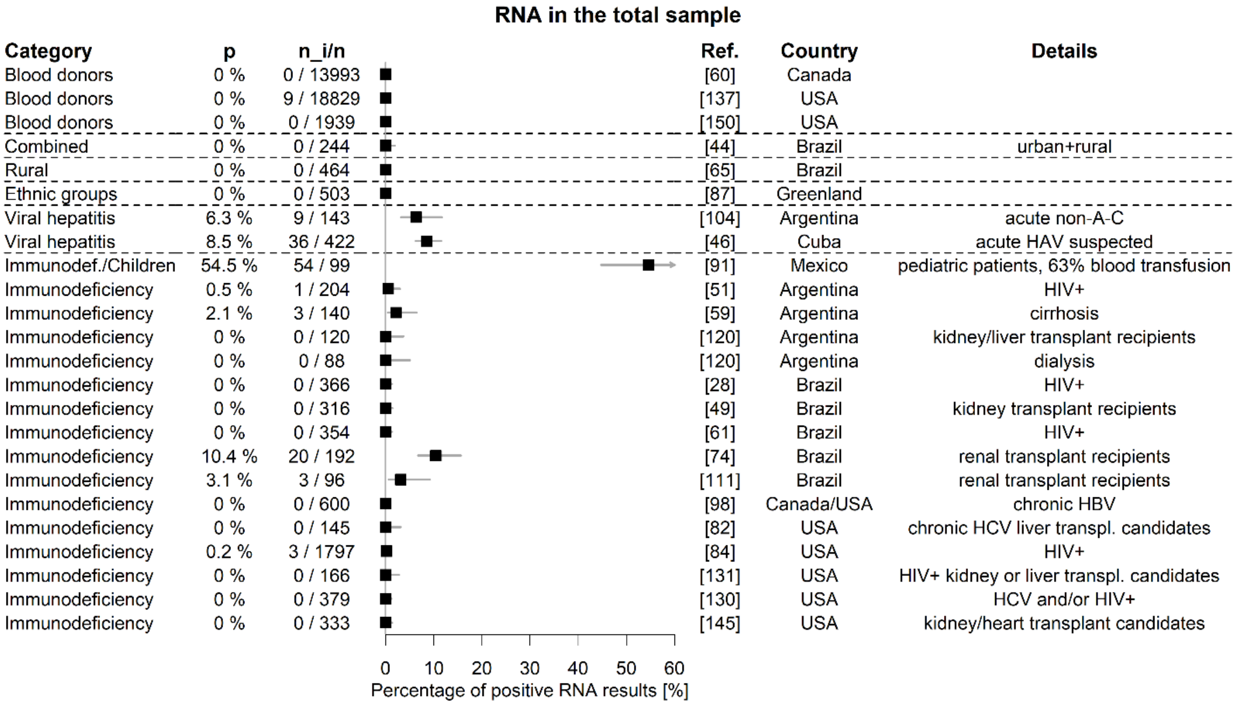

Fig 20. Percentages of RNA positive results in the total sample. Depicted are also the 95% Agresti-Coull confidence intervals for proportions truncated to the permissible range 0-100%.

15. Percentages of RNA positive results among IgG and/or IgM positive samples

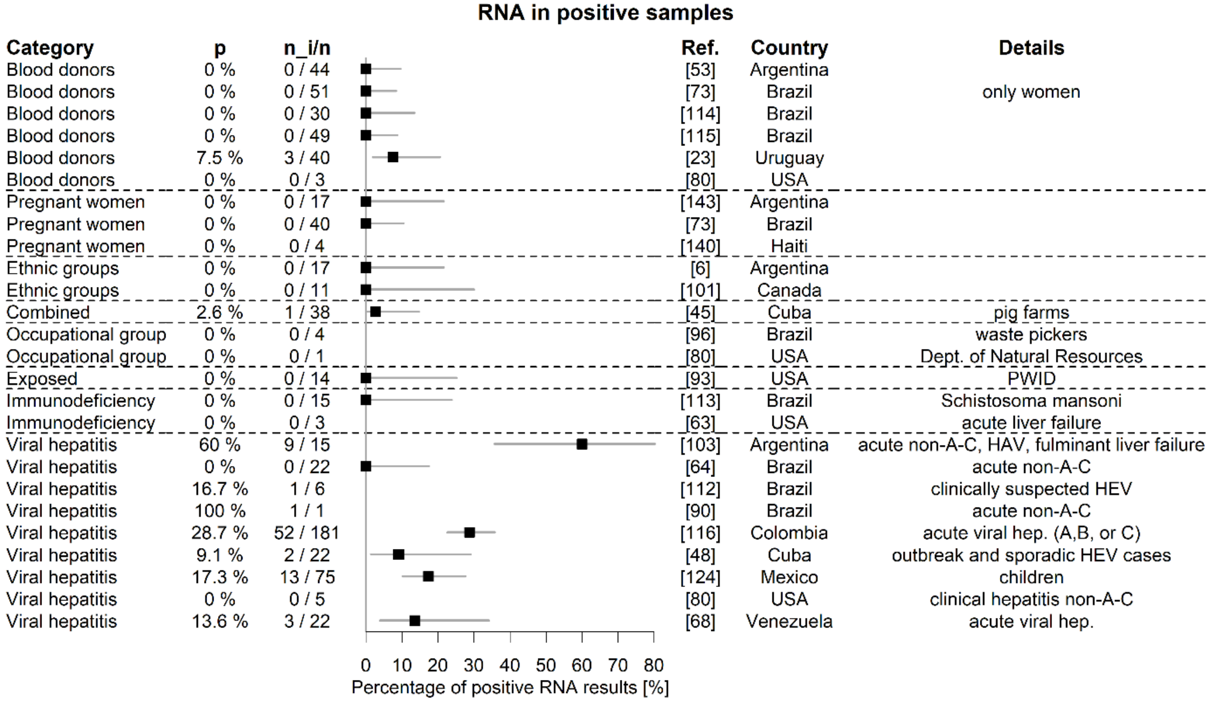

Fig 21. Percentages of RNA positive results among IgG and/or IgM positive samples. Depicted are also the 95% Agresti-Coull confidence intervals for proportions truncated to the permissible range 0-100%. Note that the intervals are not reported and results not depicted if sample size was less than 10.

16. References

1 Bigna JJ, Modiyinji AF, Nansseu JR, *et al.* Burden of hepatitis e virus infection in pregnancy and maternofoetal outcomes: A systematic review and meta-analysis. *BMC Pregnancy Childbirth* 2020; **20**: 426.
2 Capai L, Falchi A, Charrel R. Meta-analysis of human IgG anti-HEV seroprevalence in industrialized countries and a review of literature. *Viruses* 2019; **11**. DOI:10.3390/v11010084.
3 Carrilho FJ, Mendes Clemente C, Da Silva LC. Epidemiology of hepatitis A and E virus infection in Brazil. *Gastroenterol Hepatol* 2005; **28**: 118–25.
4 Horvatits T, Ozga AK, Westhölter D, *et al.* Hepatitis E seroprevalence in the Americas: A systematic review and meta-analysis. *Liver Int* 2018; **38**: 1951–64.
5 Li P, Liu J, Li Y, *et al.* The global epidemiology of hepatitis E virus infection: A systematic review and meta-analysis. *Liver Int* 2020; **40**: 1516–28.
6 Remondegui C, Ceballos S, Arce LP, *et al.* Serologic evidence of the circulation of the hepatitis E virus and the prevalence of antibodies against hepatitis A in an indigenous population in northern Argentina. *Rev Argent Microbiol* 2021. DOI:10.1016/j.ram.2020.10.006.
7 Tavakoli A, Alavian SM, Moghoofei M, Mostafaei S, Abbasi S, Farahmand M. Seroepidemiology of hepatitis E virus infection in patients undergoing maintenance hemodialysis: Systematic review and meta-analysis. *Ther Apher Dial* 2021; **25**: 4–15.
8 Tengan FM, Figueiredo GM, Nunes AKS, *et al.* Seroprevalence of hepatitis e in adults in Brazil: A systematic review and meta-analysis. *Infect Dis Poverty* 2019; **8**: 3.
9 Verghese VP, Robinson JL. A systematic review of hepatitis E virus infection in children. *Clin Infect Dis* 2014; **59**: 689–97.
10 Wilhelmi B, Waddell L, Greig J, Young I. Systematic review and meta-analysis of the seroprevalence of hepatitis e virus in the general population across non-endemic countries. *PLoS One* 2019; **14**: 1–27.
11 Wilhelm B, Waddell L, Greig J, Young I. A systematic review and meta-analysis of predictors of human hepatitis E virus exposure in non-endemic countries. *Zoonoses Public Health* 2020; **67**: 391–406.
12 Alvarado-Esquivel C, Sanchez-Anguiano LF, Hernandez-Tinoco J. Seroepidemiology of hepatitis E virus infection in general population in rural Durango, Mexico. *Hepat Mon* 2014; **14**: e16876.
13 Alvarado-Esquivel C, Gutierrez-Martinez VD, Ramirez-Valles EG, Sifuentes-Alvarez A. Hepatitis E Virus Infection and Butchers: A Case-Control Seroprevalence Study. *Gastroenterol Res* 2021; **14**: 96–103.
14 Alvarado-Esquivel C, Gutierrez-Martinez VD, Ramírez-Valles EG, Sifuentes-Alvarez A. Hepatitis E virus infection and waste pickers: A case-control seroprevalence study. *J Med Virol* 2021; **93**: 3779–85.
15 Alvarado-Esquivel C, Sanchez-Anguiano LF, Hernandez-Tinoco J. Seroepidemiology of Hepatitis E Virus Infection in Mennonites in Mexico. *J Clin Med Res* 2015; **7**: 103–8.
16 Alvarado-Esquivel C, Gutierrez-Martinez VD, Ramírez-Valles EG, Sifuentes-Alvarez A. Low prevalence of anti-hepatitis E virus IgG antibodies in Tepehuanos in Mexico. *Ann Hepatol* 2020; **19**: 186–9.
17 Alvarez-Muñoz MT, Torres J, Damasio L, Gómez A, Tapia-Conyer R, Muñoz O. Seroepidemiology of hepatitis E virus infection in Mexican subjects 1 to 29 years of age. *Arch Med Res* 1999; **30**: 251–4.
18 Andonov A, Rock G, Lin L, *et al.* Serological and molecular evidence of a plausible transmission of hepatitis E virus through pooled plasma. *Vox Sang* 2014; **107**: 213–9.
19 Anugwom C, Campbell C, Debes JD. Assessment of subclinical effects of Hepatitis E virus infection in the United States. *J Viral Hepat* 2021; **28**: 1091–7.
20 Arce LP, Müller MF, Martinez A, *et al.* A Novel In-House Enzyme-Linked Immunosorbent Assay for Genotype 3 Hepatitis E Virus Reveals High Seroprevalence in Blood Donors in Northern Argentina. *Front Microbiol* 2019; **10**: 2481.
21 Assis SB, Souto FJD, Fontes CJF, Gaspar AMC. Prevalence of hepatitis A and E virus infection in school children of an Amazonian municipality in Mato Grosso State. *Rev Soc Bras Med Trop* 2002; **35**: 155–8.
22 Atiq M, Shire NJ, Barrett A, Rouster SD, Sherman KE, Shata MT. Hepatitis E virus antibodies in patients with chronic liver disease. *Emerg Infect Dis* 2009; **15**: 479–81.
23 Bangueses F, Abin-Carriquiry JA, Cancela F, Curbelo J, Mirazo S. Serological and molecular prevalence of hepatitis E virus among blood donors from Uruguay. *J Med Virol* 2021; **93**: 4010–4.
24 Baptista-González H, Trueba-Gómez R, Rosenfeld-Mann F, Roque-Álvarez E, Méndez-Sánchez N. Low prevalence of IgG antibodies against antigens of HEV genotypes 1 and 3 in women with a high-risk pregnancy. *J Med Virol* 2017; **89**: 2051–4.
25 Bartoloni A, Bartalesi F, Roselli M, *et al.* Prevalence of antibodies against hepatitis A and E viruses among

rural populations of the Chaco region, south-eastern Bolivia. *Trop Med Int Heal* 1999; **4**: 596–601.
26 Bernal Reyes R, Licona Solís JE. Seroepidemiology of hepatitis E in the State of Hidalgo. *Rev Gastroenterol México* 1996; **61**: 233–8.
27 Betancur C, Mejía M, Portillo S. Seroprevalence of hepatitis E in swine farms workers in Aburrá Valley 2011-2012. *Acta Medica Colomb* 2013; **38**: 68–70.
28 Bezerra LA, de Oliveira-Filho EF, Silva JVJ, *et al.* Risk analysis and seroprevalence of HEV in people living with HIV/AIDS in Brazil. *Acta Trop* 2019; **189**: 65–8.
29 Blitz-Dorfman L, Monsalve F, Atencio R, *et al.* Serological survey of markers of infection with viral hepatitis among the Yukpa Amerindians from western Venezuela. *Ann Trop Med Parasitol* 1996; **90**: 655–7.
30 Bortoliero AL, Bonametti AM, Morimoto HK, Matsuo T, Reiche EMV. Seroprevalence for hepatitis E virus (HEV) infection among volunteer blood donors of the Regional Blood Bank of Londrina, State of Paraná, Brazil. *Rev Inst Med Trop Sao Paulo* 2006; **48**: 87–92.
31 Brahm B J, Hurtado H C, Moraga V M, *et al.* Infección con el virus de la hepatitis E en Chile. Comunicación preliminar. *Rev Med Chil* 1996; **124**: 947–9.
32 Bricks G, Senise JF, Pott-Jr H, *et al.* Previous hepatitis E virus infection, cirrhosis and insulin resistance in patients with chronic hepatitis C. *Brazilian J Infect Dis* 2019; **23**: 45–52.
33 Bricks G, Senise JF, Pott Junior H, *et al.* Seroprevalence of hepatitis E virus in chronic hepatitis C in Brazil. *Brazilian J Infect Dis* 2018; **22**: 85–91.
34 Cabezas C, Cabezas C, Trujillo O, *et al.* Seroepidemiology of hepatitis A, B, C, D and e virus infections in the general population of Peru: A cross-sectional study. *PLoS One* 2020; **15**: e0234273.
35 Caetano KAA, Bergamaschi FPR, Carneiro MAS, *et al.* Hepatotropic viruses (hepatitis A, B, C, D and E) in a rural Brazilian population: Prevalence, genotypes, risk factors and vaccination. *Trans R Soc Trop Med Hyg* 2020; **114**: 91–8.
36 Campbell C. Seroprevalence and Characteristics of Hepatitis E in the United States. 2019. http://search.ebscohost.com/login.aspx?direct=true&db=ddu&AN=965ED458B4B02414&site=ehost-live.
37 Campolmi I, Spinicci M, Mayaregua DR, *et al.* Seroprevalence of hepatitis a virus, hepatitis e virus, and helicobacter pylori in rural communities of the Bolivian Chaco, 2013. *Am J Trop Med Hyg* 2018; **98**: 1275–80.
38 Cangin C, Focht B, Harris R, Strunk JA. Hepatitis E seroprevalence in the United States: Results for immunoglobulins IGG and IGM. *J Med Virol* 2019; **91**: 124–31.
39 Cordova CMM, Blatt SL, Botelho TKR, Dalmarco EM. Sorologia para o vírus da Hepatite E em gestantes: clinicamente importante ou desnecessário? *Rev bras anal clin* 2007; **39**: 269–73.
40 Cossaboom CM, Heffron CL, Cao D, *et al.* Risk factors and sources of foodborne hepatitis E virus infection in the United States. *J Med Virol* 2016; **88**: 1641–5.
41 Covarrubias Ruz N, Naveas P, Miranda J, *et al.* Hepatitis E virus seroprevalence in blood donors in a university hospital in Chile. *Rev Chil Infectol* 2018; **35**: 455–7.
42 Covarrubias N, Hurtado C, Díaz A, Mezzano G, Brahm J, Venegas M. Re-evaluación de seroprevalencia de virus hepatitis E. *Rev Chil Infectol* 2015; **32**: 482–4.
43 Cruells MR, Mescia G, Gaibisso R, *et al.* Epidemiological study of hepatitis A and E viruses in different populations in Uruguay. *Gastroenterol Hepatol* 1997; **20**: 295–8.
44 de Almeida e Araújo DC, de Oliveira JM, Haddad SK, *et al.* Declining prevalence of hepatitis A and silent circulation of hepatitis E virus infection in southeastern Brazil. *Int J Infect Dis* 2020; **101**: 17–23.
45 De La Caridad Montalvo Villalba M, Owot JC, Corrreia B, *et al.* Hepatitis E virus genotype 3 in humans and swine, Cuba. *Infect Genet Evol* 2013; **14**: 335–9.
46 Montalvo-Villalba MC, Castellano-Girones Y, Bello-Corredor M, Rodríguez-Lay L de los Á. Detección del genotipo 1d del virus de la hepatitis E en pacientes con sospecha de hepatitis viral aguda, Cuba 2013. *Rev bioméd* 2016; **27**: 75–83.
47 Villalba MCM, Guan M, Pérez A, *et al.* Seroprevalence of antibodies to hepatitis E virus in two large communities in Havana, Cuba. *Trans R Soc Trop Med Hyg* 2010; **104**: 772–6.
48 Villalba MDLCM, Lay LDLAR, Chandra V, *et al.* Hepatitis E virus genotype 1, Cuba. *Emerg Infect Dis* 2008; **14**: 1320–2.
49 de Oliveira JMNS, Freitas NR de, Teles SA, *et al.* Prevalence of hepatitis E virus RNA and antibodies in a cohort of kidney transplant recipients in Central Brazil. *Int J Infect Dis* 2018; **69**: 41–3.
50 De Paula VS, Arruda ME, Vitral CL, Gaspar AMC. Seroprevalence of Viral Hepatitis in Riverine Communities from the Western Region of the Brazilian Amazon Basin. *Mem Inst Oswaldo Cruz* 2001; **96**: 1123–8.

51 Debes JD, Martínez Wassaf M, Pisano MB, *et al.* Increased hepatitis E virus seroprevalence correlates with lower CD4+ cell counts in HIV-infected persons in Argentina. *PLoS One* 2016; **11**: e0160082.

52 Dell'Amico MC, Cavallo A, Gonzales JL, *et al.* Hepatitis E virus genotype 3 in humans and swine, Bolivia. *Emerg Infect Dis* 2011; **17**: 1488–90.

53 Di Lello FA, Blejer J, Alter A, *et al.* Seroprevalence of hepatitis E virus in Argentinean blood donors. *Eur J Gastroenterol Hepatol* 2020; **Publish Ah**. DOI:10.1097/meg.0000000000001853.

54 Diehl TM, Adams DJ, Nylund CM. Ingesting self-grown produce and seropositivity for hepatitis e in the United States. *Gastroenterol Res Pract* 2018; **2018**: 7980413.

55 Ditah I, Ditah F, Devaki P, Ditah C, Kamath PS, Charlton M. Current epidemiology of hepatitis E virus infection in the United States: Low seroprevalence in the National Health and Nutrition Evaluation Survey. *Hepatology* 2014; **60**: 815–22.

56 Eick A, Ticehurst J, Tobler S, *et al.* Hepatitis E seroprevalence and seroconversion among US military service members deployed to Afghanistan. *J Infect Dis* 2010; **202**: 1302–8.

57 Engle RE, Yu C, Emerson SU, Meng XJ, Purcell RH. Hepatitis E virus (HEV) capsid antigens derived from viruses of human and swine origin are equally efficient for detecting anti-HEV by enzyme immunoassay. *J Clin Microbiol* 2002; **40**: 4576–80.

58 Fainboim H, González J, Fassio E, *et al.* Prevalence of hepatitis viruses in an anti-human immunodeficiency virus-positive population from Argentina. A multicentre study. *J Viral Hepat* 1999; **6**: 53–7.

59 Fantilli AC, Trinks J, Marciano S, *et al.* Unexpected high seroprevalence of hepatitis e virus in patients with alcohol-related cirrhosis. *PLoS One* 2019; **14**: e0224404.

60 Fearon MA, O'Brien SF, Delage G, *et al.* Hepatitis E in Canadian blood donors. *Transfusion* 2017; **57**: 1420–5.

61 Ferreira AC, Gomes-Gouvêa MS, Lisboa-Neto G, *et al.* Serological and molecular markers of hepatitis E virus infection in HIV-infected patients in Brazil. *Arch Virol* 2018; **163**: 43–9.

62 Focaccia, da Conceição OJ, Sette, *et al.* Estimated Prevalence of Viral Hepatitis in the General Population of the Municipality of São Paulo, Measured by a Serologic Survey of a Stratified, Randomized and Residence-Based Population. *Braz J Infect Dis* 1998; **2**: 269–84.

63 Fontana RJ, Engle RE, Scaglione S, *et al.* The role of hepatitis E virus infection in adult Americans with acute liver failure. *Hepatology* 2016; **64**: 1870–80.

64 de Freitas NR, de Santana EBR, da Costa e Silva ÁM, *et al.* Hepatitis E virus infection in patients with acute non-A, non-B, non-C hepatitis in Central Brazil. *Mem Inst Oswaldo Cruz* 2016; **111**: 692–6.

65 De Freitas NR, Teles SA, Caetano KAA, *et al.* Hepatitis E seroprevalence and associated factors in rural settlers in central Brazil. *Rev Soc Bras Med Trop* 2017; **50**: 675–9.

66 Gambel JM, Drabick JJ, Seriwatana J, Innis BL. Seroprevalence of hepatitis E virus among United Nations Mission In Haiti (UNMIH) Peacekeepers, 1995. *Am J Trop Med Hyg* 1998; **58**: 731–6.

67 Gandolfo GM, Ferri GM, Conti L, *et al.* Prevalence of infections by hepatitis A, B, C and E viruses in two different socioeconomic groups of children from Santa Cruz, Bolivia. *Med Clin (Barc)* 2003; **120**: 725–7.

68 García CG, Sánchez D, Villalba MCM, *et al.* Molecular characterization of hepatitis E virus in patients with acute hepatitis in Venezuela. *J Med Virol* 2012; **84**: 1025–9.

69 Goncales NSL, Pinho JRR, Moreira RC, *et al.* Hepatitis E virus immunoglobulin G antibodies in different populations in Campinas, Brazil. *Clin Diagn Lab Immunol* 2000; **7**: 813–6.

70 Gutiérrez-Vergara CC, Rodríguez B, Parra-Suescún J, *et al.* Determinación de anticuerpos totales (IgG/IgM) y específicos (IgM) para el virus de la hepatitis E y detección molecular del virus en heces de humanos con o sin exposición ocupacional a porcinos en 10 municipios de Antioquia TT - Determination of total (. *Iatreia* 2015; **28(3)**: 248–58.

71 Guzmán Rojas P, Gallegos López RC, Ciliotta Chehade A, Bravo Paredes EA, Huayanay Falconi L, Tagle Arróspide M. Seroprevalencia y factores asociados a la infección por el virus de hepatitis E en manipuladores de cerdos en Lima. *Diagnóstico (Perú)* 2013; **52**: 187–93.

72 Halac U, Béland K, Lapierre P, *et al.* Chronic hepatitis E infection in children with liver transplantation. *Gut* 2012; **61**: 597–603.

73 Hardtke S, Rocco R, Ogata J, *et al.* Risk factors and seroprevalence of hepatitis E evaluated in frozen-serum samples (2002-2003) of pregnant women compared with female blood donors in a Southern region of Brazil. *J Med Virol* 2018; **90**: 1856–62.

74 Hering T, Passos AM, Perez RM, *et al.* Past and current hepatitis E virus infection in renal transplant patients. *J Med Virol* 2014; **86**: 948–53.

75 Hurtado H C, Muñoz G G, Brahm B J. Anti-VHEIgM en casos de infección por el virus hepatitis E. *Rev*

Med Chil 2005; **133**: 645–7.
76 Hyams KC, Yarbough PO, Gray S, *et al.* Hepatitis E virus infection in Peru. *Clin Infect Dis* 1996; **22**: 719–20.
77 Humberto Ibarra V, Stella Riedemann G, Claudio Toledo A. Seguimiento de anticuerpos contra hepatitis A y E en una cohorte de niños de bajo nivel socioeconómico. *Rev Med Chil* 2006; **134**: 139–44.
78 Ibarra V H, Riedemann G S, Reinhardt V G, *et al.* Prevalencia de anticuerpos del virus Hepatitis E en donantes de bancos de sangre y otros grupos de población, en la X Región, Chile. *Rev Med Chil* 1997; **125**: 275–8.
79 Ibarra V. H, Riedemann G. S, Siegel G. F, Toledo A. C, Reinhardt V. G. Hepatitis aguda por virus A, E y no A-E en adultos chilenos a fines de los años 90. *Rev Med Chil* 2001; **129**: 523–30.
80 Karetnyi Y V., Gilchrist MJR, Naides SJ. Hepatitis E virus infection prevalence among selected populations in Iowa. *J Clin Virol* 1999; **14**: 51–5.
81 Kiesslich D, Rocha JE, Crispim MA. Prevalence of hepatitis E virus antibodies among different groups in the Amazonian basin. *Trans R Soc Trop Med Hyg* 2002; **96**: 215.
82 Koning L, Charlton MR, Pas SD, *et al.* Prevalence and clinical consequences of Hepatitis E in patients who underwent liver transplantation for chronic Hepatitis C in the United States. *BMC Infect Dis* 2015; **15**: 371.
83 Konomi N, Miyoshi C, La Fuente Zerain C, Li TC, Arakawa Y, Abe K. Epidemiology of hepatitis B, C, E, and G virus infections and molecular analysis of hepatitis G virus isolates in Bolivia. *J Clin Microbiol* 1999; **37**: 3291–5.
84 Kuniholm MH, Ong E, Hogema BM, *et al.* Acute and Chronic Hepatitis E Virus Infection in Human Immunodeficiency Virus-Infected U.S. Women. *Hepatology* 2016; **63**: 712–20.
85 Kuniholm MH, Pureell RH, McQuillan GM, Engle RE, Wasley A, Nelson KE. Epidemiology of hepatitis E virus in the United States: Results from the third national health and nutrition examination survey, 1988-1994. *J Infect Dis* 2009; **200**: 48–56.
86 Kyvernitakis A, Taremi M, Blechacz B, *et al.* Impact of hepatitis E virus seropositivity on chronic liver disease in cancer patients with hepatitis C virus infection. *Hepatol Res* 2015; **45**: 1146–51.
87 Langer BCA, Frösner GG, Von Brunn A. Epidemiological study of viral hepatitis types A, B, C, D and E among Inuits in West Greenland. *J Viral Hepat* 1997; **4**: 339–49.
88 Lemos G, Jameel S, Panda S, Rivera L, Rodríguez L, Gavilondo J V. Hepatitis E virus in Cuba. *J Clin Virol* 2000; **16**: 71–5.
89 León P, Venegas E, Bengoechea L, *et al.* Prevalencia de las infecciones por virus de las hepatitis B, C, D y E en Bolivia. *Rev Panam Salud Publica* 1999; **5**. http://www.scielosp.org/scielo.php?script=sci_arttext&pid=S1020-49891999000300002&lng=pt&nrm=iso.
90 Lopes dos Santos DR, Lewis-Ximenez LL, da Silva MFM, de Sousa PSF, Gaspar AMC, Pinto MA. First report of a human autochthonous hepatitis E virus infection in Brazil. *J Clin Virol* 2010; **47**: 276–9.
91 López-Santaella T, Álvarez y Muñoz T, Medeiros-Domingo M, *et al.* Serological and molecular study of Hepatitis E virus in pediatric patients in Mexico. *Ann Hepatol* 2020; **19**: 295–301.
92 Lyra AC, Pinho JRR, Silva LK, *et al.* HEV, TTV and GBV-C/HGV markers in patients with acute viral hepatitis. *Brazilian J Med Biol Res* 2005; **38**: 767–75.
93 Mahajan R, Collier MG, Kamili S, *et al.* Hepatitis E virus among persons who inject drugs, San Diego, California, USA, 2009-2010. *Emerg Infect Dis* 2013; **19**: 1664–6.
94 Martínez Wassaf MG, Pisano MB, Barril PA, *et al.* First detection of hepatitis E virus in Central Argentina: Environmental and serological survey. *J Clin Virol* 2014; **61**: 334–9.
95 Martínez AP, Pereson MJ, Pérez PS, *et al.* Prevalence of hepatitis E virus in children from Northeast of Argentina. *J Med Virol* 2021; **93**: 4015–7.
96 Martins RMB, Freitas NR, Kozlowski A, *et al.* Seroprevalence of hepatitis E antibodies in a population of recyclable waste pickers in Brazil. *J Clin Virol* 2014; **59**: 188–91.
97 Mast EE, Ken Kuramoto I, Favorov MO, *et al.* Prevalence of and risk factors for antibody to hepatitis E virus seroreactivity among blood donors in Northern California. *J Infect Dis* 1997; **176**: 34–40.
98 McGivern DR, Lin HHS, Wang J, *et al.* Prevalence and impact of hepatitis E virus infection among persons with chronic hepatitis B living in the US and Canada. *Open Forum Infect Dis* 2019; **6**: ofz175.
99 Meng XJ, Wiseman B, Elvinger F, *et al.* Prevalence of antibodies to hepatitis E virus in veterinarians working with swine and in normal blood donors in the United States and other countries. *J Clin Microbiol* 2002; **40**: 117–22.
100 Miernyk KM, Bruden D, Parkinson AJ, *et al.* Human Seroprevalence to 11 Zoonotic Pathogens in the U.S. Arctic, Alaska. *Vector-Borne Zoonotic Dis* 2019; **19**: 563–75.

101 Minuk GY, Sun A, Sun DF, *et al.* Serological evidence of hepatitis E virus infection in an indigenous North American population. *Can J Gastroenterol* 2007; **21**: 439–42.
102 Moraes Dos Santos DC, Souto FJD, Lopes Dos Santos DR, Vitral CL, Gaspar AMC. Seroepidemiological markers of enterically transmitted viral hepatitis A and E in individuals living in a community located in the north area of Rio de Janeiro, RJ, Brazil. *Mem Inst Oswaldo Cruz* 2002; **97**: 637–40.
103 Munné MS, Altabert NR, Vladimirsky SN, *et al.* Identifications of polyphyletic variants in acute hepatitis suggest an underdiagnosed circulation of hepatitis E virus in Argentina. *J Clin Virol* 2011; **52**: 138–41.
104 Munné MS, Altabert NR, Otegui M LO, *et al.* Updating the knowledge of hepatitis E: new variants and higher prevalence of anti-HEV in Argentina. *Ann Hepatol* 2014; **13**: 496–502.
105 Ooi WW, Gawoski JM, Yarbough PO, Pankey GA. Hepatitis E seroconversion in United States travelers abroad. *Am J Trop Med Hyg* 1999; **61**: 822–4.
106 Pandolfi R, De Almeida DR, Pinto MA, Kreutz LC, Frandoloso R. In house ELISA based on recombinant ORF2 protein underline high prevalence of IgG antihepatitis e virus amongst blood donors in south Brazil. *PLoS One* 2017; **12**: e0176409.
107 Panduro A, Meléndez GE, Fierro NA, Madrigal BR, Zepeda-Carrillo EA, Román S. Epidemiología de las hepatitis virales en México. *Salud Publica Mex* 2011; **53**: S37-45.
108 Pang L, Alencar FEC, Cerutti C, *et al.* Short report: Hepatitis E infection in the Brazilian Amazon. *Am J Trop Med Hyg* 1995; **52**: 347–8.
109 Parana R, Cotrim HP, Cortey-Boennec ML, Trepo C, Lyra L. Prevalence of hepatitis E virus IgG antibodies in patients from a referral unit of liver diseases in Salvador, Bahia, Brazil. *Am J Trop Med Hyg* 1997; **57**: 60–1.
110 Paraná R, Vitvitski L, Andrade Z, *et al.* Acute sporadic non-A, non-B hepatitis in northeastern Brazil: Etiology and natural history. *Hepatology* 1999; **30**: 289–93.
111 Passos AM, Heringer TP, Medina-Pestana JO, Ferraz MLG, Granato CFH. First report and molecular characterization of hepatitis E virus infection in renal transplant recipients in Brazil. *J Med Virol* 2013; **85**: 615–9.
112 Passos-Castilho AM, de Sena A, Reinaldo MR, Granato CFH. Hepatitis E virus infection in Brazil: Results of laboratory-based surveillance from 1998 to 2013. *Rev Soc Bras Med Trop* 2015; **48**: 468–70.
113 Passos-Castilho AM, de Sena A, Domingues ALC, *et al.* Hepatitis E virus seroprevalence among schistosomiasis patients in Northeastern Brazil. *Brazilian J Infect Dis* 2016; **20**: 262–6.
114 Passos-Castilho AM, de Sena A, Geraldo A, Spada C, Granato CFH. High prevalence of hepatitis E virus antibodies among blood donors in Southern Brazil. *J Med Virol* 2016; **88**: 361–4.
115 Passos-Castilho AM, Reinaldo MR, Sena A de, Granato CFH. High prevalence of hepatitis E virus antibodies in Sao Paulo, Southeastern Brazil: analysis of a group of blood donors representative of the general population. *Brazilian J Infect Dis* 2017; **21**: 535–9.
116 Peláez-Carvajal D, Martínez-Vargas D, Escalante-Mora M, Palacios-Vivero M, Contreras-Gómez, Lady. Coinfection of hepatitis E virus and other hepatitis virus in Colombia and its genotypic characterization. *Biomedica* 2016; **36**: 69–78.
117 Peláez D, Hoyos MC, Rendón JC, *et al.* Infección por el virus de la hepatitis E en pacientes con diagnóstico clínico de hepatitis viral en Colombia. *Biomedica* 2014; **34**: 354–65.
118 Perez OM, Morales W, Paniagua M, Strannegard O. Prevalence of antibodies to hepatitis A, B, C, and E viruses in a healthy population in Leon, Nicaragua. *Am J Trop Med Hyg* 1996; **55**: 17–21.
119 Pisano MB, Lugo BC, Poma R, *et al.* Environmental hepatitis E virus detection supported by serological evidence in the northwest of Argentina. *Trans R Soc Trop Med Hyg* 2018; **112**: 181–7.
120 Pisano MB, Balderramo D, Wassaf MM, *et al.* Hepatitis E virus infection in patients on dialysis and in solid organ transplant recipients in Argentina: exploring associated risk factors. *Arch Virol* 2017; **162**: 787–92.
121 Pujol FH, Favorov MO, Marcano T, *et al.* Prevalence of antibodies against hepatitis e virus among urban and rural populations in Venezuela. *J Med Virol* 1994; **42**: 234–6.
122 Pujol FH, Rodríguez I, Martínez N, *et al.* Viral hepatitis serological markers among pregnant women in Caracas, Venezuela: implication for perinatal transmission of hepatitis B and C. *G E N* 1994; **48**: 25–8.
123 Quintana A, Sanchez L, Larralde O, Anderson D. Prevalence of antibodies to hepatitis E virus in residents of a district in Havana, Cuba. *J Med Virol* 2005; **76**: 69–70.
124 Realpe-Quintero M, Mirazo S, Viera-Segura O, *et al.* Hepatitis e Virus Genotype 1 and Hepatitis A Virus Dual Infection in Pediatric Patients with a Low Socioeconomic Status from Mexico. *Intervirology* 2018; **61**: 105–10.
125 Redlinger T, O'Rourke K, Nickey L, Martinez G. Elevated hepatitis A and E seroprevalence rates in a

Texas/Mexico border community. *Tex Med* 1998; **94**: 68–71.
126 Rendon J, Hoyos MC, Di Filippo D, *et al.* Hepatitis E virus genotype 3 in Colombia: Survey in patients with clinical diagnosis of viral hepatitis. *PLoS One* 2016; **11**: 1–12.
127 Rey JA, Findor JA, Daruich JR, *et al.* Prevalence of IgG anti-HEV in Buenos Aires, a nonendemic area for hepatitis E. *J Travel Med* 1997; **4**: 100–1.
128 Rodríguez Lay L de los A, Quintana A, Montalvo Villalba MC, *et al.* Dual infection with hepatitis A and E viruses in outbreaks and in sporadic clinical cases: Cuba 1998-2003. *J Med Virol* 2008; **80**: 798–802.
129 SARACENI CP. Vigilância Das Hepatites Virais: a Experiência De Vargem Grande Paulista, 1997-1999. Fac. Saúde Pública da Univ. São Paulo. 2001; : 11.
130 Sherman KE, Kottilil S, Rouster SD, *et al.* Hepatitis e Infection in a Longitudinal Cohort of Hepatitis C Virus and HCV/HIV Coinfected Persons. *AIDS Res Hum Retroviruses* 2021; **37**: 534–41.
131 Sherman KE, Terrault N, Barin B, Rouster SD, Shata MT. Hepatitis e infection in HIV-infected liver and kidney transplant candidates. *J Viral Hepat* 2014; **21**: e74-7.
132 da Silva SMT, de Oliveira JM, Vitral CL, de Almeida Vieira K, Pinto MA, Dutra Souto FJ. Prevalence of hepatitis E virus antibodies in individuals exposed to swine in Mato Grosso, Brazil. *Mem Inst Oswaldo Cruz* 2012; **107**: 338–41.
133 Smalligan RD, Lange WR, Frame JD, Yarbough PO, Frankenfield DL, Hyams KC. The risk of viral hepatitis A, B, C, and E among North American missionaries. *Am J Trop Med Hyg* 1995; **53**: 233–6.
134 Smith HM, Reporter R, Rood MP, *et al.* Prevalence study of antibody to ratborne pathogens and other agents among patients using a free clinic in downtown Los Angeles. *J Infect Dis* 2002; **186**: 1673–6.
135 Souto FJD, Fontes CJF, Parana R, Lyra LGC. Short report: Further evidence for hepatitis E in the Brazilian Amazon. *Am J Trop Med Hyg* 1997; **57**: 149–50.
136 Souto FJD, Fontes CJF. Prevalence of IgG-class antibodies against hepatitis E virus in a community of the southern Amazon: A randomized survey. *Ann Trop Med Parasitol* 1998; **92**: 623–5.
137 Stramer SL, Moritz ED, Foster GA, *et al.* Hepatitis e virus: Seroprevalence and frequency of viral RNA detection among US blood donors. *Transfusion* 2016; **56**: 481–8.
138 Sue PK, Pisanic N, Heaney CD, *et al.* Hepatitis E virus infection among solid organ transplant recipients at a North American transplant center. *Open Forum Infect Dis* 2016; **3**: ofw006.
139 Talarmin A, Kazanji M, Cardoso T, Pouliquen JF, Sankale-Suzanon J, Sarthou JL. Prevalence of antibodies to hepatitis A, C, and E viruses in different ethnic groups in French Guiana. *J Med Virol* 1997; **52**: 430–5.
140 Tejada-Strop A, Tohme RA, Andre-Alboth J, *et al.* Seroprevalence of Hepatitis A and Hepatitis e viruses among pregnant women in Haiti. *Am J Trop Med Hyg* 2019; **101**: 230–2.
141 Teshale EH, Denniston MM, Drobeniuc J, Kamili S, Chong-Gee T, Holmberg SD. Decline in hepatitis e virus antibody prevalence in the United States from 1988-1994 to 2009-2010. *J Infect Dis* 2015; **211**: 366–73.
142 Thomas DL, Yarbough PO, Vlahov D, *et al.* Seroreactivity to hepatitis E virus in areas where the disease is not endemic. *J Clin Microbiol* 1997; **35**: 1244–7.
143 Tissera G, Lardizabal MC, Torres SB, *et al.* Hepatitis e virus infection in pregnant women, Argentina. *BMC Infect Dis* 2020; **20**: 368.
144 Trinta KS, Liberto MIM, De Paula VS, Yoshida CFT, Gaspar AMC. Hepatitis E Virus Infection in Selected Brazilian Populations. *Mem Inst Oswaldo Cruz* 2001; **96**: 25–9.
145 Unzueta A, Valdez R, Chang YHH, *et al.* Hepatitis E virus serum antibodies and RNA prevalence in patients evaluated for heart and kidney transplantation. *Ann Hepatol* 2016; **15**: 33–40.
146 Vildosola H, Colichón A, Barreda M, Piscoya J, Palacios O. [HEPATITIS E IgG ANTIBODIES SEROPREVALENCE IN A PERUVIAN RISK GROUP]. *Rev Gastroenterol Peru* 2000; **20**: 111–6.
147 Vitral CL, da Silva-Nunes M, Pinto MA, *et al.* Hepatitis A and E seroprevalence and associated risk factors: A community-based cross-sectional survey in rural Amazonia. *BMC Infect Dis* 2014; **14**: 458.
148 Vitral CL, Pinto MA, Lewis-Ximenez LL, Khudyakov YE, Dos Santos DR, Gaspar AMC. Serological evidence of hepatitis E virus infection in different animal species from the Southeast of Brazil. *Mem Inst Oswaldo Cruz* 2005; **100**: 117–22.
149 Withers MR, Correa MT, Morrow M, *et al.* Antibody levels to hepatitis E virus in North Carolina swine workers, non-swine workers, swine, and murids. *Am J Trop Med Hyg* 2002; **66**: 384–8.
150 Xu C, Wang RY, Schechterly CA, *et al.* An assessment of hepatitis e virus (HEV) in US blood donors and recipients: No detectable HEV RNA in 1939 donors tested and no evidence for HEV transmission to 362 prospectively followed recipients. *Transfusion* 2013; **53**: 2505–11.
151 Zafrullah M, Zhang X, Tran C, *et al.* Disparities in detection of antibodies against hepatitis E virus in US

blood donor samples using commercial assays. *Transfusion* 2018; **58**: 1254–63.
152 Zhang L, Yesupriya A, Chang MH, Teshale E, Teo CG. Apolipoprotein E and protection against hepatitis E viral infection in American non-Hispanic blacks. *Hepatology* 2015; **62**: 1346–52.
153 Huerta Lorenzo B, Tarradas-Iglesias C, González M, *et al.* Seroprevalencia y factores asociados a la infección por el virus de la hepatitis E (Genotipo 3) en perros del sur de España. *An la Real Acad Ciencias Vet Andalucía* 2011; **24**: 135–44.
